# Design and Pictet–Spengler enabled synthesis of carboxamide-substituted imidazo[1,2-*a*]quinoxalines as dual EGFR and tubulin targeting anticancer agents

**DOI:** 10.1080/14756366.2026.2673744

**Published:** 2026-05-25

**Authors:** Joydeep Chatterjee, Gaurav Joshi, Vibhu Jha, Shivkanya M. Bhujbal, Shivani Rathour, Sanjana Majumdar, Muhammad Wahajuddin, Prasad V. Bharatam, Raj Kumar

**Affiliations:** a Laboratory for Drug Design and Synthesis, Department of Pharmaceutical Sciences and Natural Products, School of Health Sciences, Central University of Punjab, Bathinda, India; b Department of Pharmaceutical Science, Hemvati Nandan Bahuguna Garhwal (A Central) University, Srinagar, India; c Institute of Cancer Therapeutics, School of Pharmacy, Optometry and Medical Sciences, University of Bradford, Bradford, UK; d Department of Medicinal Chemistry, National Institute of Pharmaceutical Education and Research (NIPER), Punjab, India

**Keywords:** EGFR kinase inhibition, tubulin polymerisation, dual-target anticancer agents, ROS-mediated apoptosis, molecular docking and dynamics

## Abstract

In this study, we report the Pictet–Spengler enabled synthesis of a series of eighteen carboxamide-substituted imidazo[1,2-*a*]quinoxaline derivatives (**JRC-1-JRC-18**) targeting epidermal growth factor receptor (EGFR) and tubulin. Compounds **JRC-2** and **JRC-6** exhibited potent antiproliferative effects against MCF-7 breast cancer cells, with IC_50_ values of 4.59 ± 0.23 µM and 4.01 ± 0.14 µM, respectively, outperforming erlotinib (IC_50_ = 9.39 ± 0.16 µM). In enzymatic assays, **JRC-2** and **JRC-6** inhibited wild-type EGFR with IC_50_ values of 294.45 nM and 383.90 nM, respectively. Notably, **JRC-6** displayed microtubule-stabilising activity comparable to that of paclitaxel and induced ROS generation, mitochondrial membrane depolarisation, and G2/M phase cell cycle arrest. Molecular docking and molecular dynamics simulations confirmed stable binding of compounds at the EGFR ATP-binding site and the tubulin taxol-binding site.

## GRAPHICAL ABSTRACT

**Figure:**
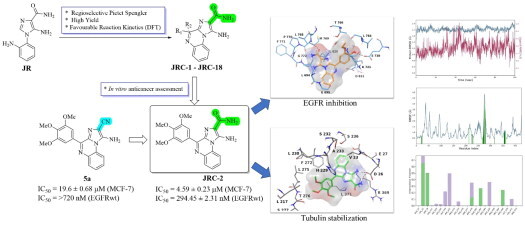

## Introduction

**Figure 1.**
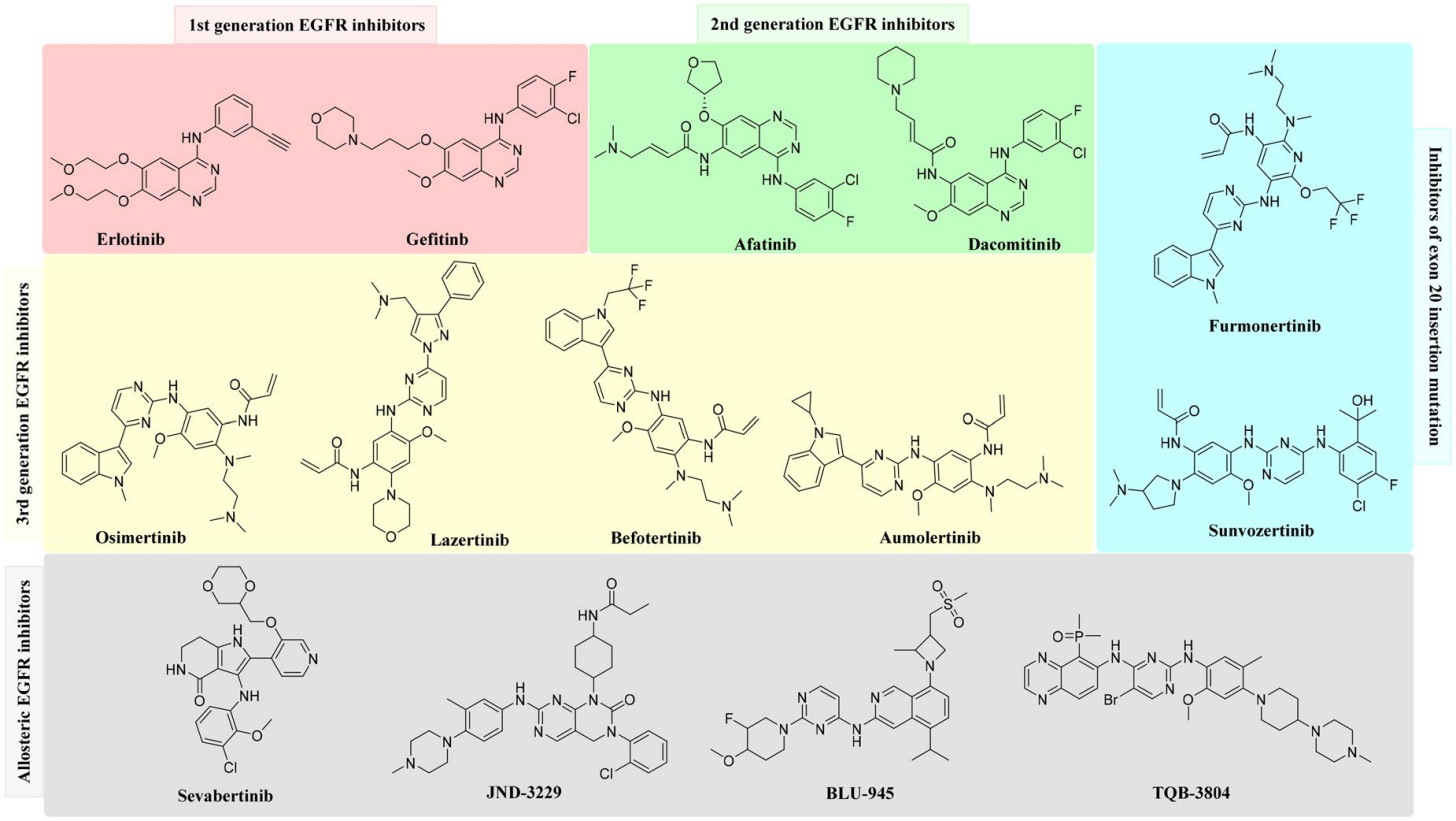
EGFR inhibitors of different generations to overcome resistance and mutations.

According to the International Agency for Research on Cancer (IARC), approximately 20 million new cases of cancer were estimated to have occurred in 2022. Among all cancer types, lung cancer (12.4% of all cancer types) is the most frequently diagnosed cancer type, followed by breast cancer (11.6% of all cancer types), where one in every nine men and one out of twelve women die due to cancer^1^. Currently, there are 14 hallmarks of cancer, of which the Epidermal Growth Factor Receptor (EGFR) kinase and tubulin are regulators of proliferative signalling^2^^,^^3^. Along with protein phosphorylation, EGFR has been found to play a diagnostic, predictive, and prognostic biomarker of various cancer types, including non-small cell lung cancer (NSCLC), breast, colorectal, and pancreatic cancer. Over several decades, various EGFR inhibitors have been approved by the US FDA, while a few are currently in clinical trials. The first generation of EGFR inhibitors (Figure 1), such as erlotinib and gefitinib, were reversible ATP-competitive inhibitors primarily used to treat activating mutations. Drugs like afatinib and dacomitinib are second-generation irreversible EGFR inhibitors known for their covalent interactions. Third-generation EGFR inhibitors like osimertinib, lazertinib, and aumolertinib are reversible inhibitors primarily designed to target the T790M mutation, also known as the gatekeeper mutation. However, all these generations of drugs suffer from one or the other adverse effects, such as gastrointestinal, cutaneous, and cardiac toxicity, along with a major challenge of acquired resistance, which emphasises the need to develop novel EGFR inhibitors. Currently, befortinib is another drug approved in China, but it is under clinical trial as a second-line treatment for patients with the T790M mutation^4^. Moreover, furmonertinib and sunvozertinib, approved for exon 20 insertion mutations, have paved the way for a new direction in anti-EGFR drug design^5–8^. Several clinical trials are underway with drugs such as sevabertinib, JND-3229, BLU-945, and TQB-3804 to overcome the C797S mutation and develop novel allosteric EGFR inhibitors^8^.

Signalling through EGFR can occur either in the cytoplasm (also known as canonical) or in the nucleus (non-canonical)^9^. In a cytoplasmic signalling pathway, the activated EGFR undergoes phosphorylation, initiating a series of downstream signalling pathways that eventually lead the signal to the nucleus, thereby triggering gene expression. In the non-canonical pathway, the entire receptor can shuttle into the nucleus, leading to gene expression. The latter process primarily involves understanding cases of drug resistance to anticancer regimens^10^.

One of the fundamental elements of the eukaryotic cytoskeleton is the microtubules, which are responsible for diverse cellular functions, including cellular trafficking, mitotic spindle formation, and maintenance of cellular morphology. Structurally, they are long, cylindrical polymers composed of 13 parallel protofilaments that collectively form a hollow tube, known as the microtubule. Each protofilament is composed of α and β-heterodimers that are assembled in a head-to-tail arrangement. Both contain a guanosine triphosphate (GTP) binding domain that is primarily responsible for microtubule assembly and disassembly^11^. In the case of α-tubulin, GTP binds at the non-exchangeable site (N-site). In contrast, for β-tubulin, GTP binds at the exchangeable site (E-site), where it is hydrolysed to guanosine diphosphate (GDP). Both these states of β-tubulin determine the microtubule dynamics, where GDP-bound β-tubulin induces depolymerisation, and GTP-bound β-tubulin induces microtubule stabilisation and elongation^12^^,^^13^. This dynamic instability enables microtubules to respond to various cellular signals, playing a crucial role in maintaining the spatial and temporal regulation of cytoskeletal architecture^14^.

Microtubule-targeting agents (MTAs) are a privileged class of anticancer molecules that have established a significant role in cancer therapeutics^11^^,^^14^^,^^15^ by either inhibiting (e.g. combretastatin A-4, cryptophycin) or promoting tubulin polymerisation (e.g. paclitaxel). Despite a wide range of activities against various cancer types, they face significant resistance issues, for instance, primarily due to the activation of signal transduction pathways, such as EGFR overexpression^16–19^ and adverse drug reactions like neurotoxicity and myelosuppression. Various combination therapies have been developed to overcome such issues, and some clinical trials have targeted both EGFR and tubulin simultaneously. Clinical trials of erlotinib in combination with tubulin inhibitors (NCT00360360, NCT00059787), gefitinib (NCT01196234), or afatinib (NCT02511847) have shown efficacy; however, combination therapy itself faces challenges, including drug-drug interactions, unbalanced dose regimens, and poor multidrug pharmacokinetic profiles. To overcome these challenges, a multi-targeted approach targeting both EGFR and tubulin could be an alternative medicinal chemistry strategy^20^^,^^21^. Moreover, dual-targeting inhibitors offer several benefits over conventional single-targeting agents, including increased patient tolerance, reduced risk of feedback activation of secondary pathways, reduced risk of dose-dependent toxicity and drug resistance, and increased chance of cancer remission^22–26^.

**Figure 2.**
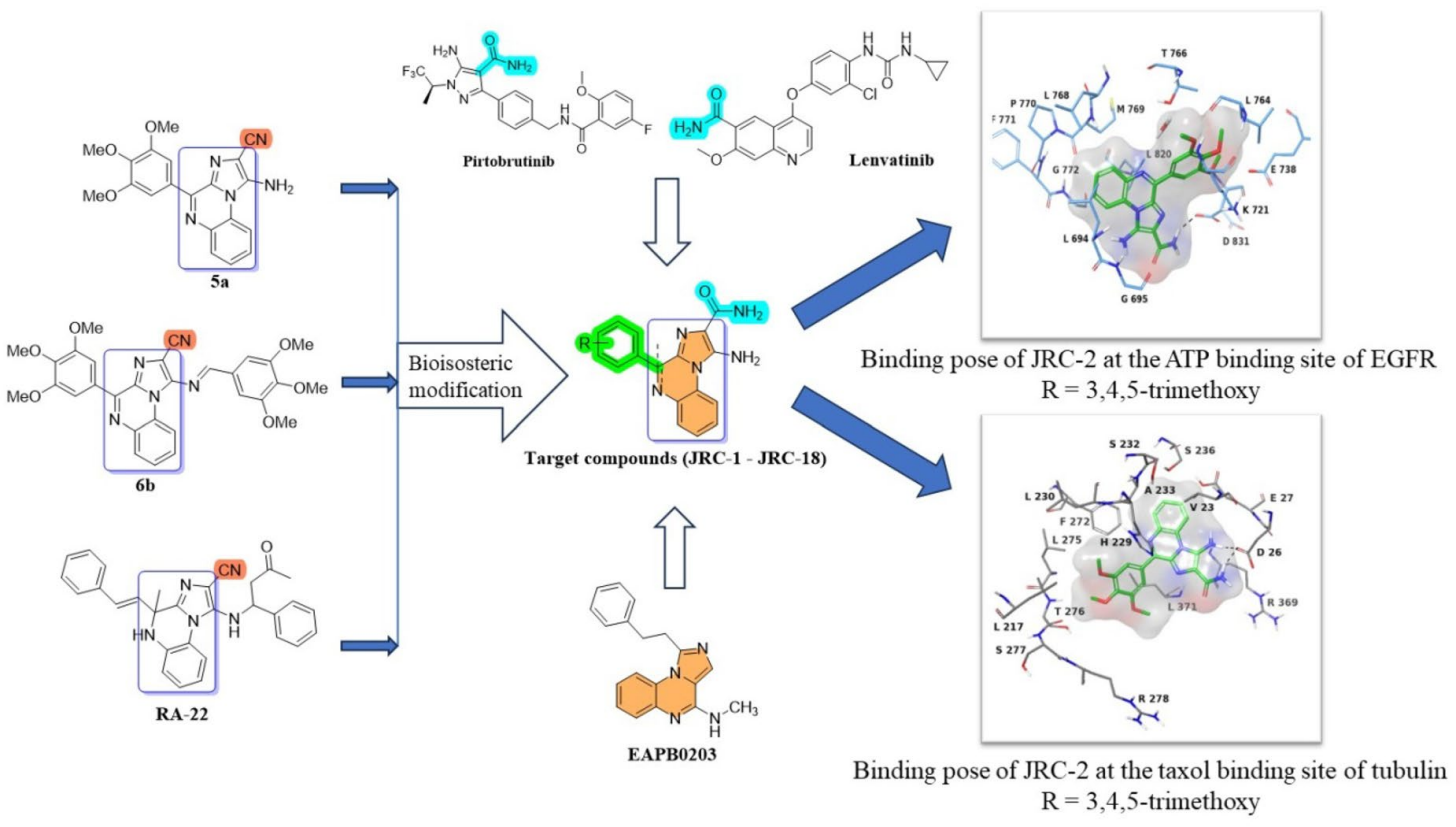
Rationale for the design of target compounds (**JRC-1–18**).

Earlier, our research group demonstrated the use of novel imidazo[1,2-*a*]quinoxaline derivatives (**6b** and **RA-22**; Figure 2) as EGFR inhibitors, serving as an alternative scaffold to the conventional quinazoline framework^27–31^. **Compound 6b** (Figure 2) showed promising results, with cellular IC_50_ values of 3.65 µM (H1975) and 2.7 µM (A549), and an enzymatic IC_50_ of 211.22 nM against wild-type EGFR. Moreover, the compound inhibited tumour growth in a human lung cancer xenograft model in nude mice^27^^,^^28^. Similarly, another molecule (**Compound RA-22,** Figure 2) exhibited EGFR inhibitory activity with an IC_50_ of 266.38 nM^30^. Furthermore, the quinoxaline scaffold is known to exhibit tubulin-binding properties, e.g. as seen with EAPB0203^32^. The results of our study indicated a few key features: (a) imidazo[1,2-*a*]quinoxaline can serve as an alternative scaffold to quinazoline and exhibits promising EGFR inhibitory potential; (b) iminic substitution at 5-NH_2_ (as in the case of compound **6b**) showed improved activity in comparison to compound **5a** (no iminic substitution)^28^; and (c) the nitrile group interacted with a few amino acid residues in the ATP binding site of EGFR.

**Figure 3.**
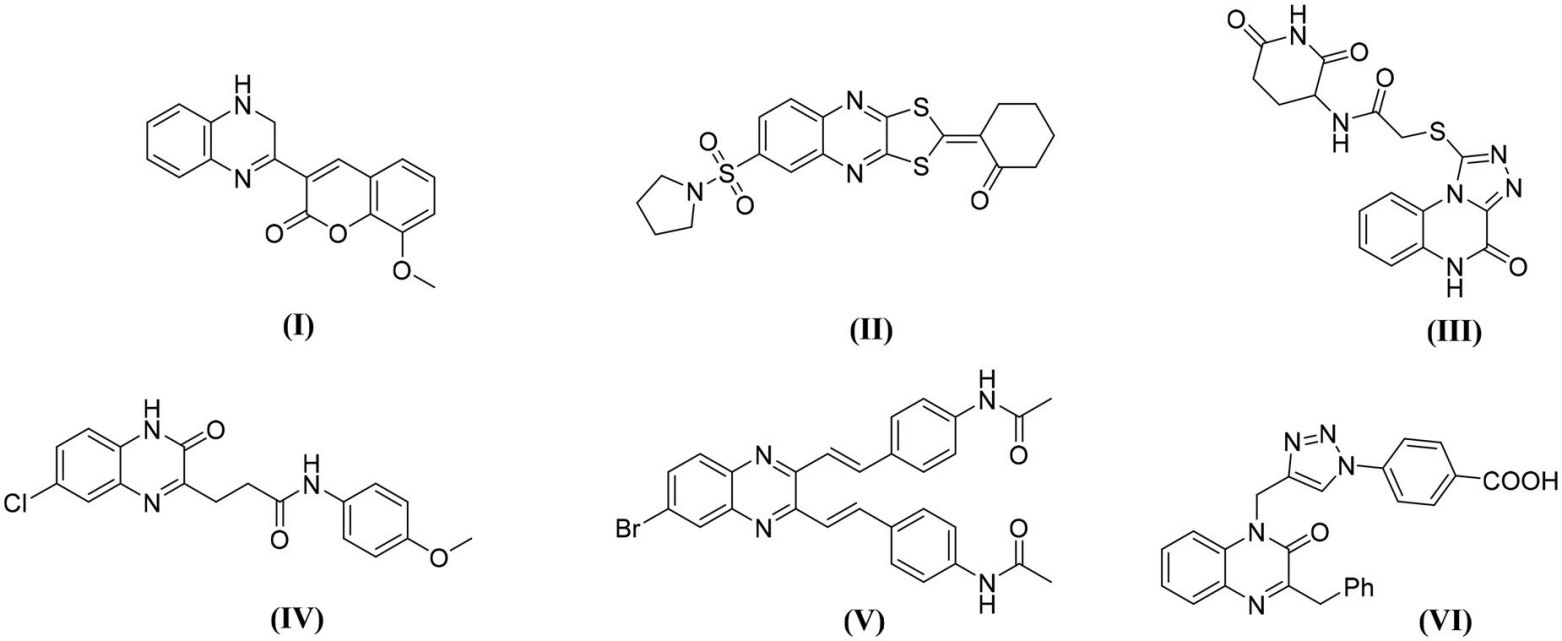
Reported quinoxaline-based EGFR inhibitors.

Furthermore, several other research groups have also contributed to the development of quinoxaline or quinoxaline fused derivatives targeting different types of cancer via EGFR (**Compound I-VI,** Figure 3)^33–38^.

In the present work, to develop a Structure-Activity-Relationship (SAR) for discovering promising EGFR inhibitors, we thought of retaining the imidazo[1,2-*a*]quinoxaline pharmacophore and replacing the nitrile group (**5a**, **6b**, and **RA-22**) with a carboxamide group, leading to the design of target EGFR inhibitors (**JRC-1 – JRC-18,** Figure 2), anticipating that bio-isosteric modification would help improve the pharmacokinetic and pharmacodynamic properties in terms of metabolic stability, improved bioavailability, hydrogen bond donor-acceptor properties, and better hydrophilic-lipophilic balance^39^^,^^40^. Moreover, FDA-approved carboxamide-containing anticancer drugs targeting BTK (zanubrutinib)^41^, (pirtobrutinib)^38^, FMS-like tyrosine kinase 3 (gilteritinib)^37^, VEGFR (lenvatinib)^30^, and PARP-1 & PARP-2 (niraparib)^31^ also serve as key supporting examples and explain the role of carboxamide in designing kinase inhibitors. Interestingly, preliminary docking results at the ATP-binding site of EGFR (PDB: 1M17) indicated that **JRC-2** (a representative compound) successfully occupies the erlotinib binding site, which is flanked by key amino acid residues, including Leu764, Thr766, Met769, and Leu768. This sequence of amino acids (760–770) is known as the hinge region of wild-type EGFR, which is crucial for inhibitory activity. Moreover, a hydrogen-bonding interaction was also observed with Asp831, which resides in the kinase-specific pocket. Comparative binding interactions were also conducted for the nitrile-containing compound **5a**, but no promising engagement was observed, which justifies its EGFR inactivity. Similarly, molecular docking was conducted at the taxol-binding site of tubulin, where we observed a similar binding interaction between our test compound, **JRC-2**, and paclitaxel (positive control). Both compounds bind to the key amino acid sequence comprising Asp26, Val23, Ala233, and His229. These observations encouraged us to develop **JRC** compounds targeting both EGFR and tubulin.

**Scheme 1.**
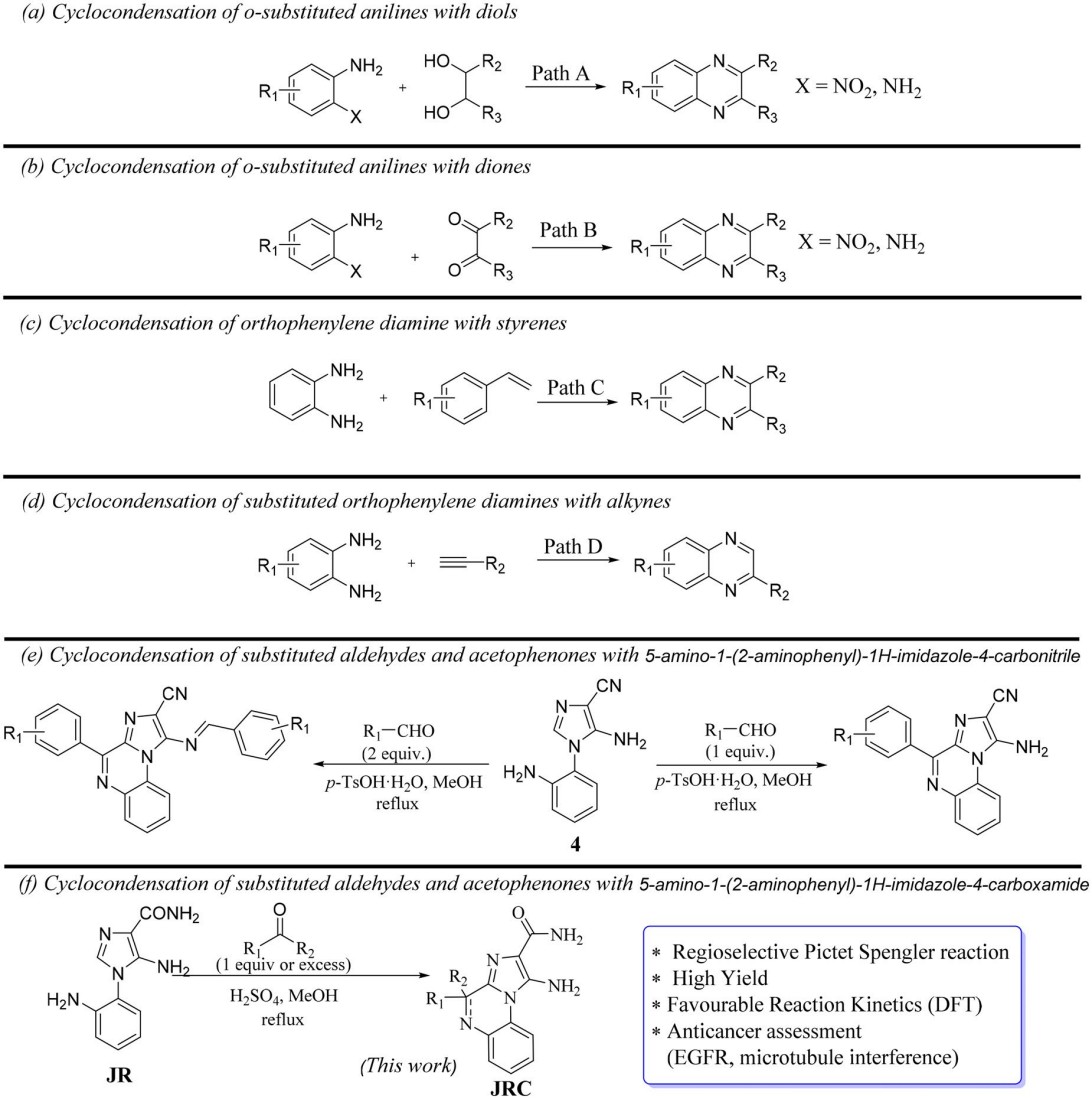
Reported methodologies (paths a–e) and proposed work (f) for the synthesis of various quinoxaline analogues

For the construction of the quinoxaline scaffold, cyclocondensation of *o*-substituted anilines with (a) diols (Scheme 1, Path A) in the presence of the iron-sulfur catalyst system^42^ or in the presence of a Mn-complex^43^, (b) diones (Scheme 1, Path B) under various non-catalytic systems consisting of Fe^44^, Zr^45^, and Ni^46^, and (c) with various styrenes, alkenes, and alkynes in the presence of catalytic conditions, like NBS^47^, TsNBr_2_^48^_,_ and Cu(OAc)_2_^49^ (Scheme 1, Path C and D) are reported. Our research group reported the synthesis of imidazo[1,2-*a*]quinoxaline derivatives by acid-catalyzed modified Pictet-Spengler (P-S) condensation of the 5-amino-1–(2-aminophenyl)-1*H*-imidazole-4-carbonitrile (**4**) with diverse aldehydes or ketones (Scheme 1e)^31^. In the present work, we applied the acid-catalyzed regio- and chemoselective P-S cyclo-condensation of a novel 5-amino-1–(2-aminophenyl)-1*H*-imidazole-4-carboxamide substrate (**JR**) with diverse aldehydes and ketones, affording the target compounds (**JRC**) in high yields (Scheme 1f), followed by their characterisation spectra, and DFT studies. Furthermore, their *in vitro* cell- and enzyme-based anticancer assessments, followed by molecular docking and simulations, are presented to demonstrate their anticancer potential.

## Results and discussion

### Chemistry

**Scheme 2.**
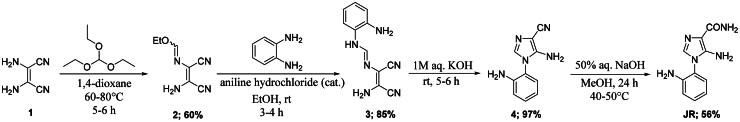
Synthetic scheme for the intermediate 5-amino-1–(2-aminophenyl)-1*H*-imidazole-4-carboxamide (**JR)**

Key intermediate 5-amino-1–(2-aminophenyl)-1*H*-imidazole-4-carboxamide (**JR**) was synthesised through a pre-established synthetic methodology^31^. Briefly, diaminomaleonitrile (**1**) undergoes condensation with triethyl orthoformate in the presence of 1,4-dioxane at 60–80 °C to afford the formation of an imidate ester (**2**). This was further reacted with o-phenylenediamine in the presence of a catalytic amount of aniline hydrochloride in ethanol, which led to the formation of (*E*)-*N’*-((*Z*)-2-amino-1,2-dicyanovinyl)-*N*-(2-aminophenyl)formimidamide (**3**). This undergoes base-catalyzed cyclisation in the presence of 1 M aq. KOH to form the key intermediate 5-amino-1–(2-aminophenyl)-1*H*-imidazole-4-carbonitrile (**4**) in moderate yield (65–75%). Purified intermediate **4** was further subjected to base-catalyzed hydrolysis in the presence of 50% aq. NaOH in MeOH to afford the formation of our key intermediate 5-amino-1–(2-aminophenyl)-1*H*-imidazole-4-carboxamide (**JR**) (yield 50–60%) (Scheme 2). This step was also performed with other base catalysts, such as TEA, DBU, DIPEA, and KOH; however, in all cases, a low yield (10–20%) of **JR** was observed.

**Scheme 3.**
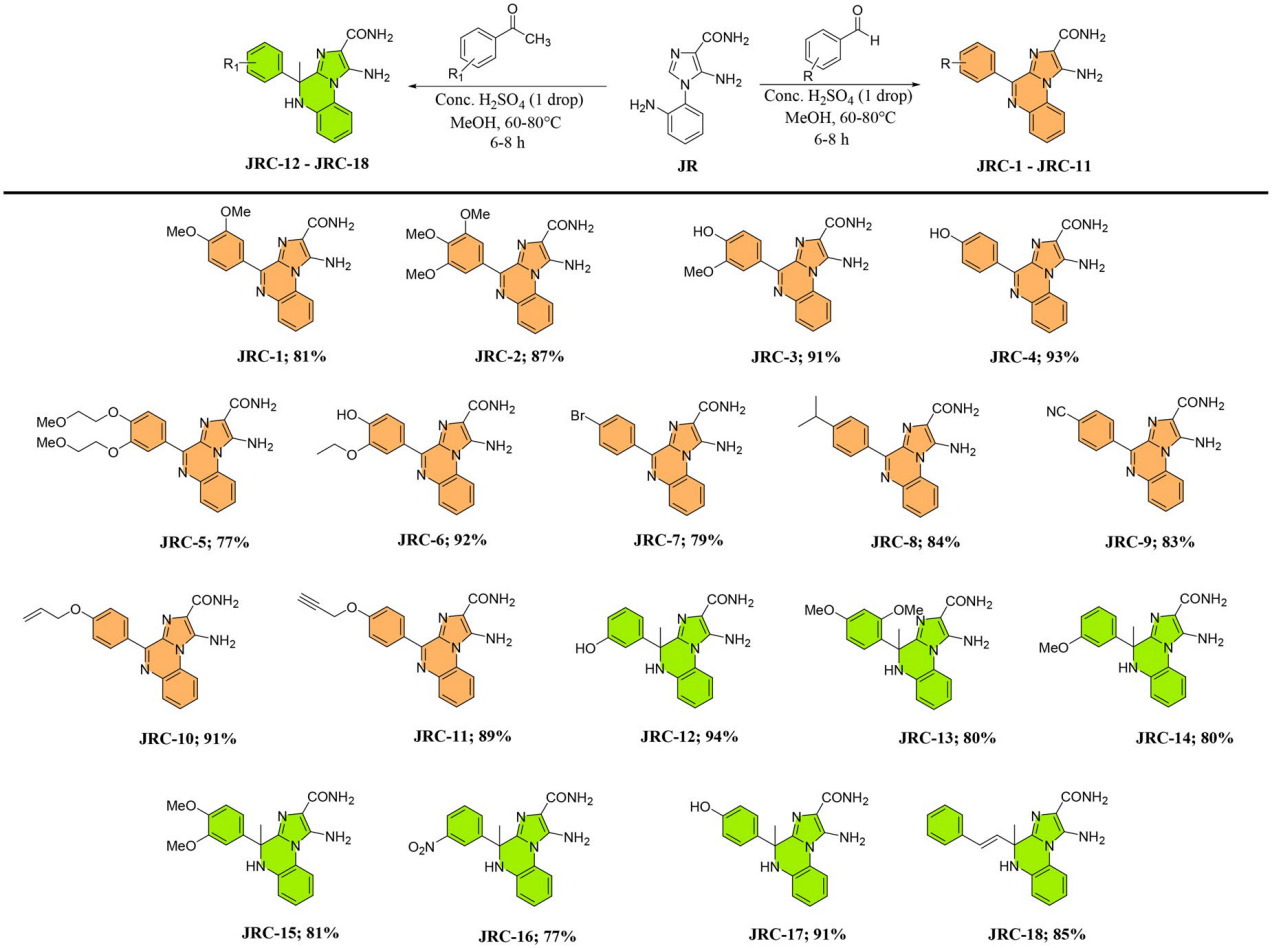
Synthesis of target compounds (**JRC-1 – JRC-18**)

Compound **JR** was further reacted with an equimolar amount of diverse substituted benzaldehydes or acetophenones to undergo modified P-S reaction, yielding various imidazo[1,2-*a*]quinoxaline-2-carboxamide (**JRC-1**–**JRC-11**) and 4,5-dihydroimidazo[1,2-a]quinoxaline-2-carboxamide (**JRC-12**–**JRC-18**) derivatives (Scheme 3), respectively, in the presence of H_2_SO_4_ (cat.) in MeOH under reflux conditions in 2–8 h. The reaction was compatible with electron-donating or withdrawing substituents. All the compounds were fully characterised by spectroscopic techniques, including IR, NMR, and HRMS. The IR spectrum showed a sharp absorption band for the nitrile group at 2210 cm-1 for the intermediate compound **4**, which was absent in the **JR** spectrum (Supplementary Material). The ^13^C NMR indicated the characteristic carbonyl of the carboxamide in the range of δ 165.7 to δ 167.1, whereas the ^1^H NMR showed two different singlet peaks for the two amide D_2_O exchangeable protons, e.g. at δ 7.4 and δ 7.6 (compound **JRC-1**). Moreover, for compounds **JRC-12** to **JRC-18**, the presence of a methyl was observed in the ^1^H NMR at around δ 1.8. The ^13^C NMR indicated the presence of a methyl at around δ 28.5 and a saturated carbon of the 4,5-dihydroimidazo[1,2-*a*]quinoxaline-2-carboxamide derivatives at δ 57.1.

**Scheme 4.**
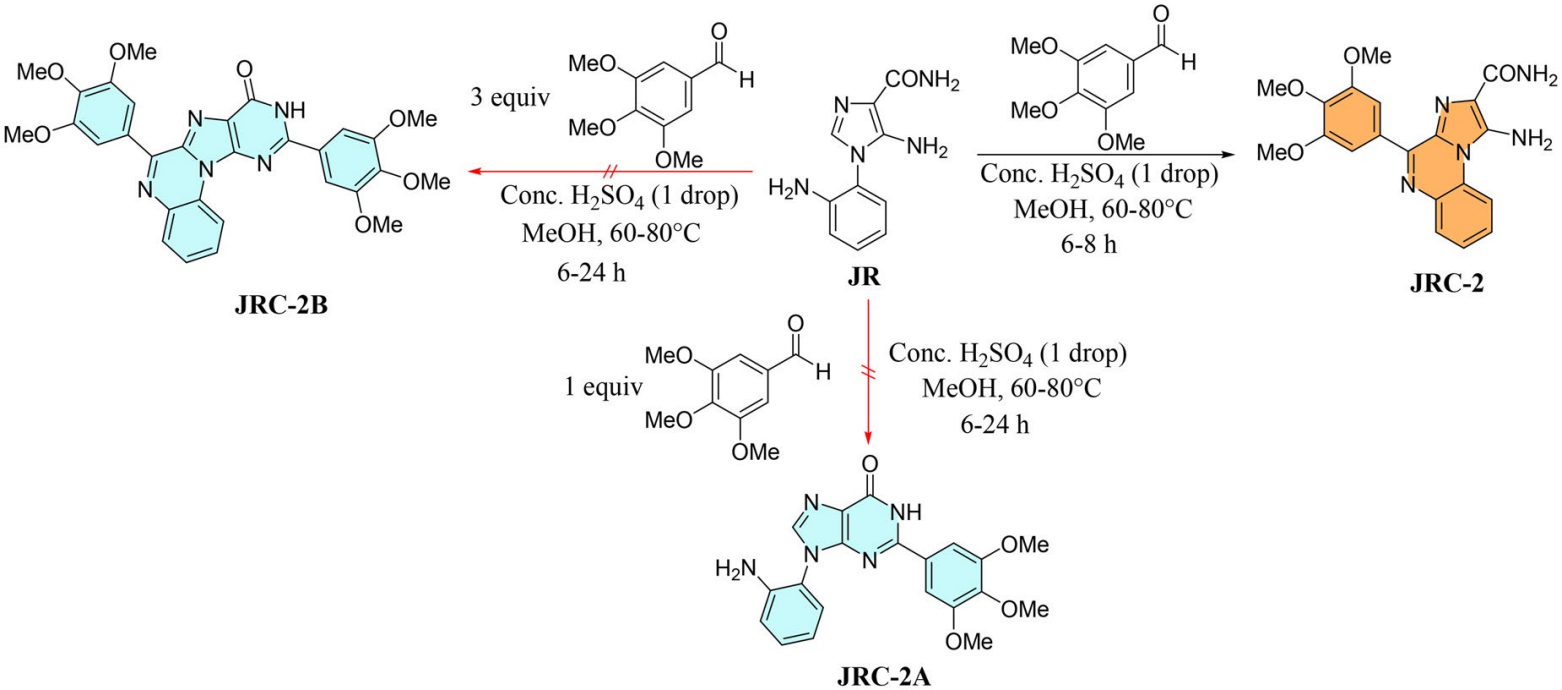
Possible reaction conditions for the formation of purine or oxopurine compounds

Interestingly, **JR**, when reacted with an excess of 3,4,5-trimethoxy benzaldehyde (2–3 equiv) in the presence of an acid catalyst, either in protic (MeOH, EtOH), aprotic polar (DMSO), or nonpolar solvents (acetonitrile, diethyl ether, toluene), did not yield either oxopurine product (**JRC**-**2A** and **JRC**-**2B**) or a fully cyclo-condensed product (**JRC**-**2B**) formed via P-S reaction, followed by purine ring formation, proving that reaction was regio- and chemoselective (Scheme 4).

**Scheme 5.**
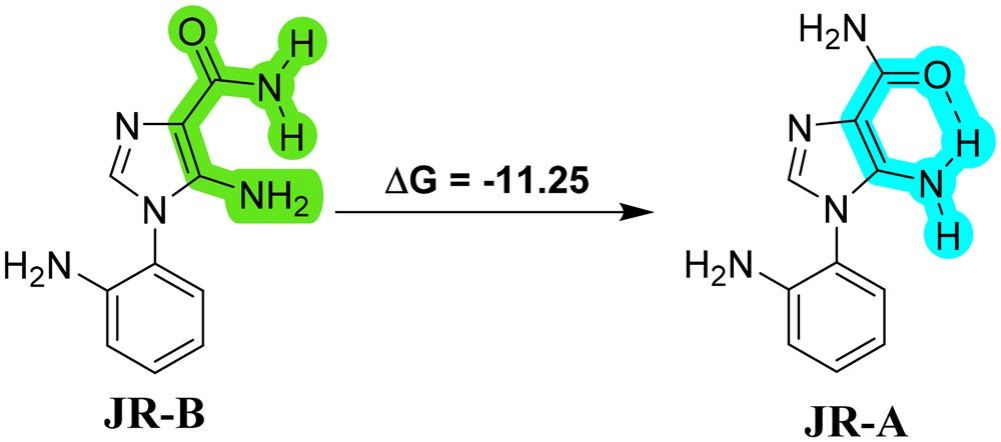
Intramolecular H-bonding in **JR-A** and **B** forms.

To justify the regioselective P-S product formation, Density Functional Theory (DFT) analysis was performed using the B3LYP/6–311 + G(d,p) level, in gas-phase geometries of all reactants. The quantum chemical calculations were carried out using the Gaussian 16 program. For the starting material, 5-amino-1–(2-aminophenyl)-1*H*-imidazole-4-carboxamide (**JR**), intramolecular hydrogen bonding was observed, and it exists in two conformers, **JR**-**A** and **JR**-**B**. The **A** form is more stable than **B** by −11.25 kcal/mol, due to the intramolecular hydrogen bonding between the carbonyl of carboxamide and 5-NH_2_ groups (Scheme 5). This could affect the final product formation; it may be why the oxopurine ring formation or the P-S reaction has not occurred at the carboxamide end. Moreover, earlier reports in the literature describe intramolecular hydrogen bonding in the presence of carboxamide^50–52^.

**Scheme 6.**
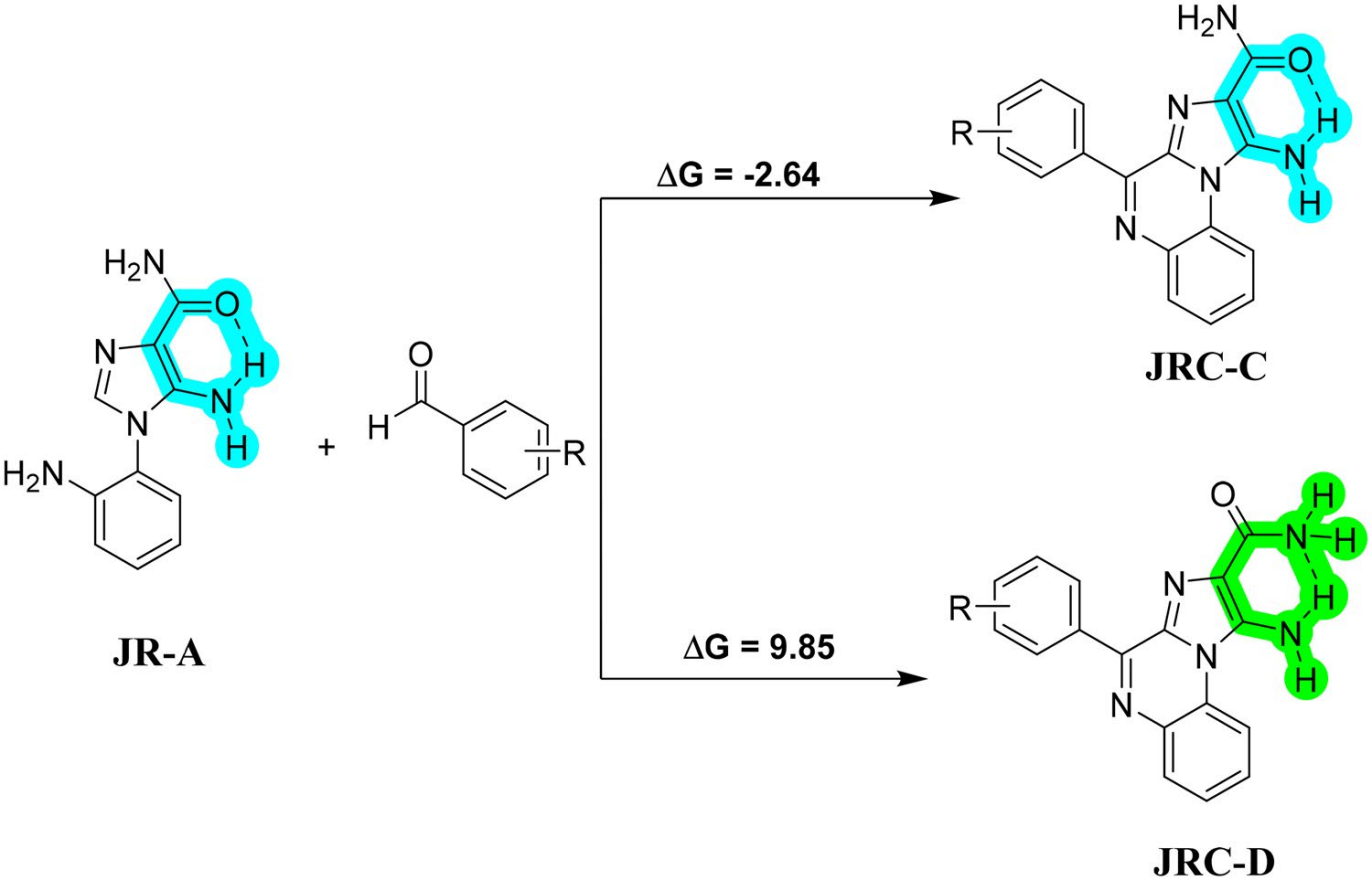
Role of intra-molecular hydrogen bonding indicating the favourable formation of **JRC-C**. The free energy values are in kcal/mol, calculated in gas phase at B3LYP/6–311 + G(d,p) level.

Intramolecular hydrogen bonding was found to influence the formation of the final product (**JRC**-**C** or **D**) (Scheme 6). The calculated overall energy for the formation of **JRC**-**C** in the presence of hydrogen bonding is −2.64 kcal/mol, indicating that the process is exergonic. In contrast, the formation of **JRC-B** is endergonic (ΔG +9.03 kcal/mol).

### Biology

#### Cell viability assay of synthetics

**Figure 4.**
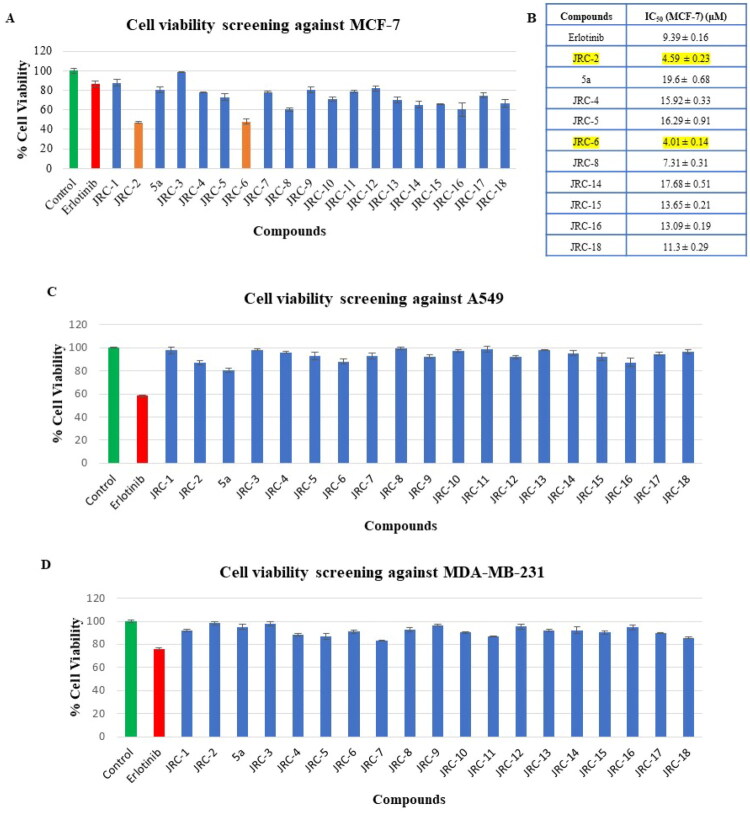
(A) Cell viability data of all the synthetics against MCF-7 cells, treated at 10 µM for 24h; (B) Calculated IC_50_ (µM ± SD) values of the compounds against MCF-7 cells; (C) Cell viability data of all the synthetics against A549 cells, treated at 10 µM for 24h; (D) Cell viability data of all the synthetics against MDA-MB-231 cells, treated at 10 µM for 24h. Data are expressed as mean values ± SD of triplicate experiments.

The antiproliferative potential of all the synthetics (**JRC-1–18**) and **5a** (for comparative study) was evaluated using the MTT assay (3–(4,5-dimethylthiazol-2-yl)-2,5-diphenyltetrazolium bromide). Initially, all target compounds were screened at 10 µM for 24 h against three different cancer cell lines (A549, MCF-7, and MDA-MB-231; Figure 4A,C,D). Erlotinib was used as a positive control. Interestingly, most of the compounds showed increased sensitivity against MCF-7 cells; however, all the compounds were practically inactive against A549 and MDA-MB-231 cells. A few of the compounds (**JRC-2, JRC-4–6, JRC-8, JRC-14–16, and JRC-18**) showed potent cytotoxicity against MCF-7; therefore, we further determined their IC_50_ values at three concentrations (1, 5, and 25 µM) using erlotinib as a positive control, and treated them for 24h. Two compounds, **JRC-2** and **JRC-6**, showed the most promising IC_50_ values of 4.59 ± 0.23 µM and 4.01 ± 0.14 µM, respectively, against MCF-7 (Figure 4B and Figure S1), values that are better than erlotinib (IC_50_ of 9.39 ± 0.16 µM). Furthermore, **5a** (nitrile-substituted) showed an IC_50_ of 19.6 ± 0.68 µM, which was fourfold higher than that of its corresponding carboxamide-substituted analogue **JRC-2,** indicating the importance of a carboxamide and supporting our hypothesis.

All the potent molecules were further evaluated against HEK-293 (human embryonic kidney cell line) to assess their potential toxicity profile, and no noticeable toxicity was observed up to 25 µM concentration of the test compounds (Figure S2).

#### EGFR and tubulin enzymatic assay of investigational compounds

**Figure 5.**
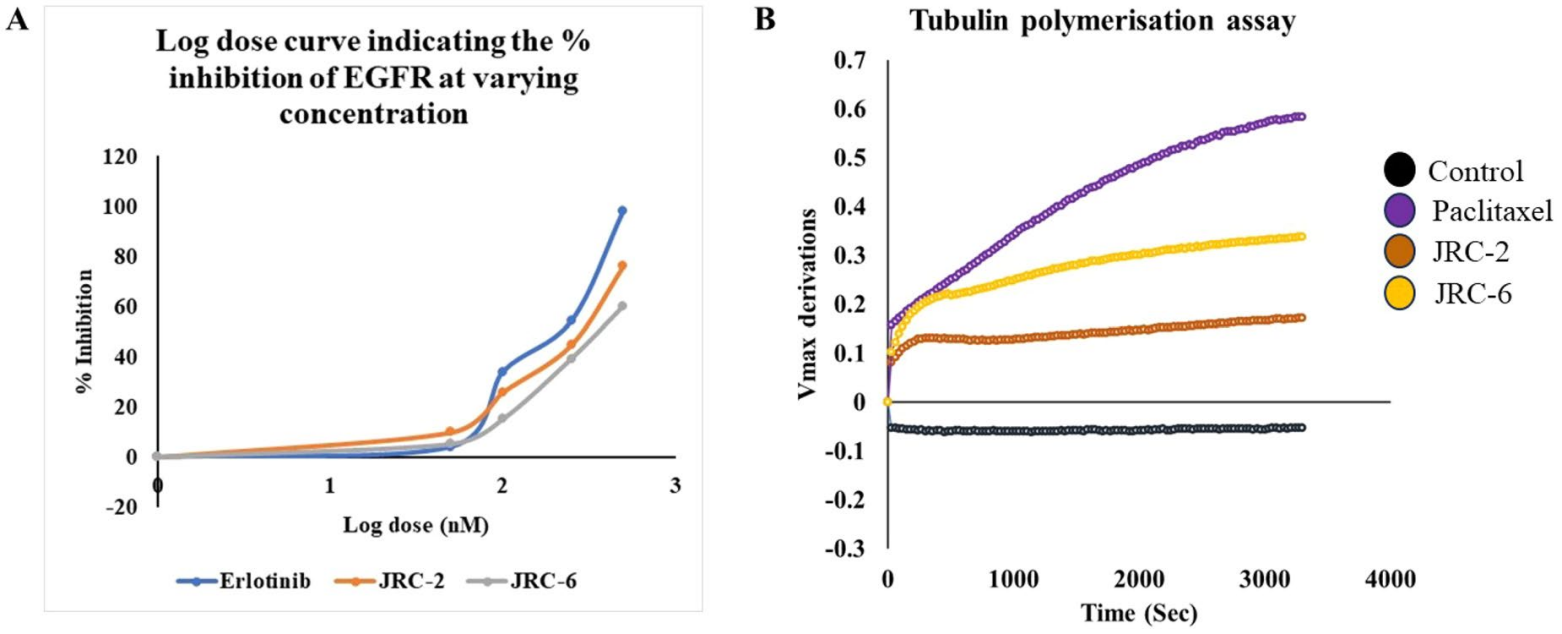
(A) Enzymatic log dose-response curve of the potent compounds **JRC-2, JRC-6,** and erlotinib against wild-type EGFR; (B) Rate of tubulin polymerisation over time for the investigational compounds **JRC-2** and **JRC-6** with respect to positive control paclitaxel, where the y-axis indicates rate of polymerisation over time (x-axis).

Potent compounds (**JRC-2** and **JRC-6**) were further tested for their enzymatic inhibitory potential using the ATP-dependent EGFR phosphorylation assay (Figure 5A) and the tubulin polymerisation assay (Figure 5B). For the EGFR enzymatic assay, IC_50_ values were determined using varying concentrations of the test compounds and the positive control, ranging from 50, 100, 250, and 500 nM, respectively. Both compounds, **JRC-2** and **JRC-6**, showed mild to comparable inhibitory potential compared to the positive control, erlotinib. Compound **JRC-2** showed an IC_50_ of 294.45 ± 2.31 nM, and compound **JRC-6** showed an IC_50_ of 383.9 ± 4.67 nM, whereas the IC_50_ of erlotinib was found to be 219.4 ± 2.19 nM against wild-type EGFR. However, the tubulin polymerisation assay was performed at 5 µM to investigate the role of the investigational molecules in maintaining tubulin dynamics. The assay was performed, using paclitaxel as a positive control, and microtubule formation was measured at an optical density (OD) of 340 nm. Both compounds (**JRC-2** and **JRC-6**) initially increased the Vmax of tubulin polymerisation; however, **JRC-6** achieved a higher Vmax than **JRC-2**. Furthermore, we observed that **JRC-6** achieved a more rapid stabilisation of tubulin dynamics. Thus, it indicates that compound **JRC-6** is a potential hit, serving as both a dual inhibitor of microtubule-interacting agents and an EGFR inhibitor.

#### Effect of investigational compounds on ROS generation, mitochondrial membrane potential, and cell cycle arrest

The ROS assay indicates alterations in reactive oxygen species, a characteristic feature of cancer cells. In the H_2_DCFDA assay, the dye readily crosses the cellular membrane, and it is converted to H_2_DCF in the presence of various cellular esterases. This H_2_DCF anion undergoes further oxidation to DCF, which exhibits characteristic fluorescence. The extent of ROS-mediated apoptosis can be estimated using this assay, which is then correlated with the JC-1 assay to determine whether apoptosis is mitochondria-dependent or independent. Some compounds tend to lower ROS levels, while others increase ROS generation, thereby causing cytotoxicity by elevating mitochondrial oxidative stress^53^^,^^54^.

**Figure 6.**
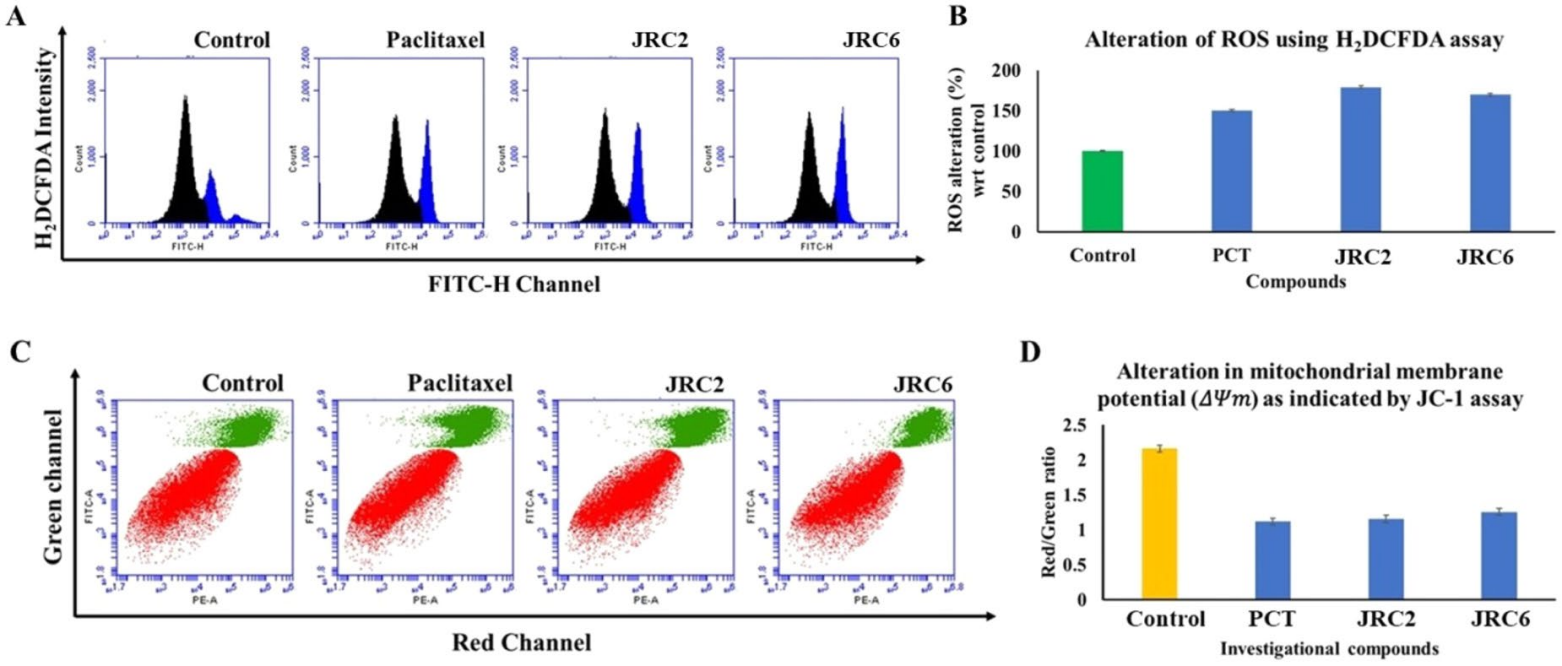
(A) ROS-dependent alteration of cellular population carried out via flow cytometry; (B) Relative ratio of change in the ROS levels; (C) JC-1 assay of **JRC-2** and **JRC-6** showing alteration of membrane polarisation at sub-IC_50_ concentration against MCF-7; (D) Relative ratio between J-aggregates and monomers induced by **JRC-2, JRC-6,** and paclitaxel at their respective sub-IC_50_ concentration against MCF-7.

**Figure 7.**
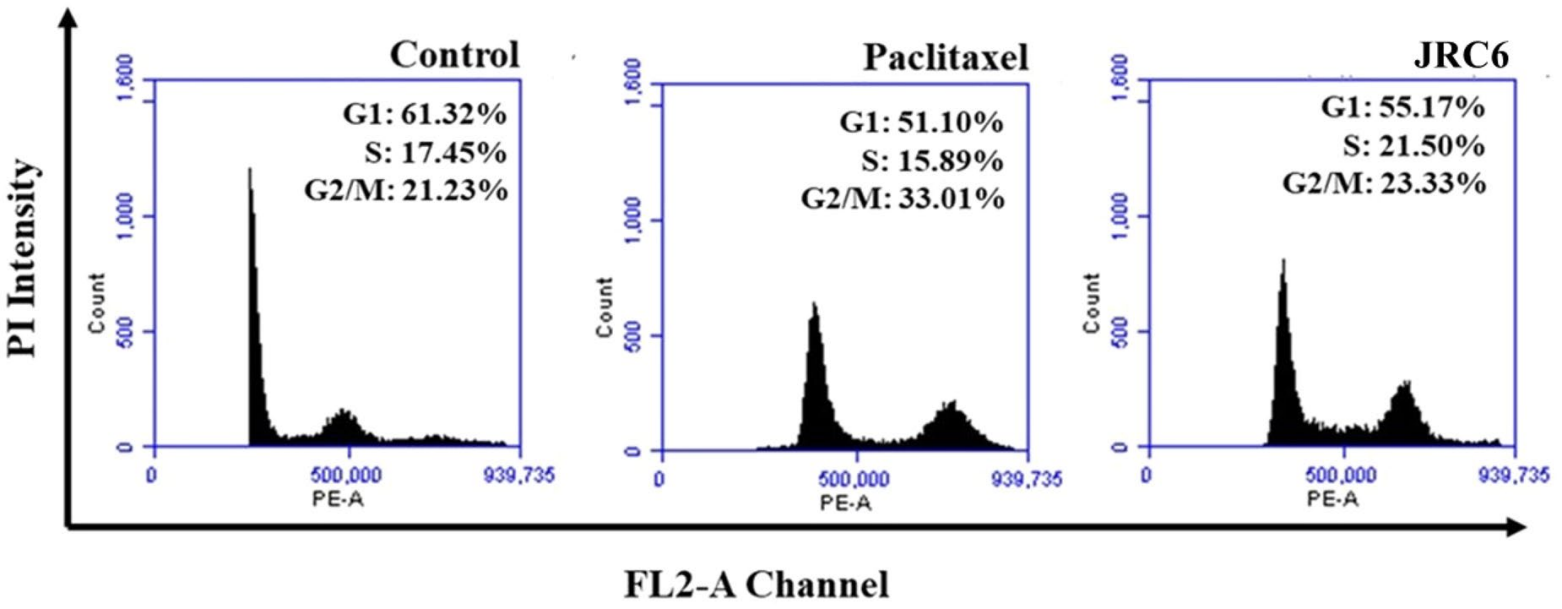
Propidium iodide assay of investigational compound **JRC-6** at sub-IC_50_ concentration against MCF-7 cells.

Both experimental compounds, **JRC-2** and **JRC-6**, strongly induced intracellular ROS accumulation in MCF-7 cells after 24h of treatment at their specific sub-IC_50_ concentrations, similar to the positive control, paclitaxel (Figure 6A,B). Interestingly, both compounds induced greater ROS upregulation than paclitaxel. These observations further prompted us to investigate mitochondrial oxidative stress using JC-1 staining. In this regard, MMP or Δψm values have been measured to assess membrane depolarisation induced by these experimentally designed compounds, a clear indicator of apoptosis. In normal cells, JC-1 accumulates in mitochondria and forms J-aggregates, emitting red fluorescence. Conversely, when mitochondria are depolarised during apoptosis, JC-1 staining remains in the cytosol as monomers, resulting in green fluorescence. The findings revealed a strong ability of both experimental compounds, JRC-2 and JRC-6, at their respective sub-IC_50_ concentrations to induce mitochondrial membrane depolarisation in MCF-7 cells after 24h of treatment, similar to that observed with paclitaxel treatment (Figure 6C,D). Next, a propidium iodide staining assay was performed in MCF-7 cells at sub-IC_50_ concentrations of **JRC-6** and paclitaxel to analyse the mechanism of cell cycle arrest. The findings indicated that **JRC-6** induced G2/M phase arrest, comparable to paclitaxel. This showed that, whereas 21% of cells remained in the G2/M phase in the control, more than 23% remained in the G2/M phase with **JRC-6** treatment, and a more pronounced G2/M arrest of 33% was observed with paclitaxel treatment (Figure 7).

### In silico studies

#### Molecular docking studies of investigational compounds against EGFR and tubulin

**Figure 8.**
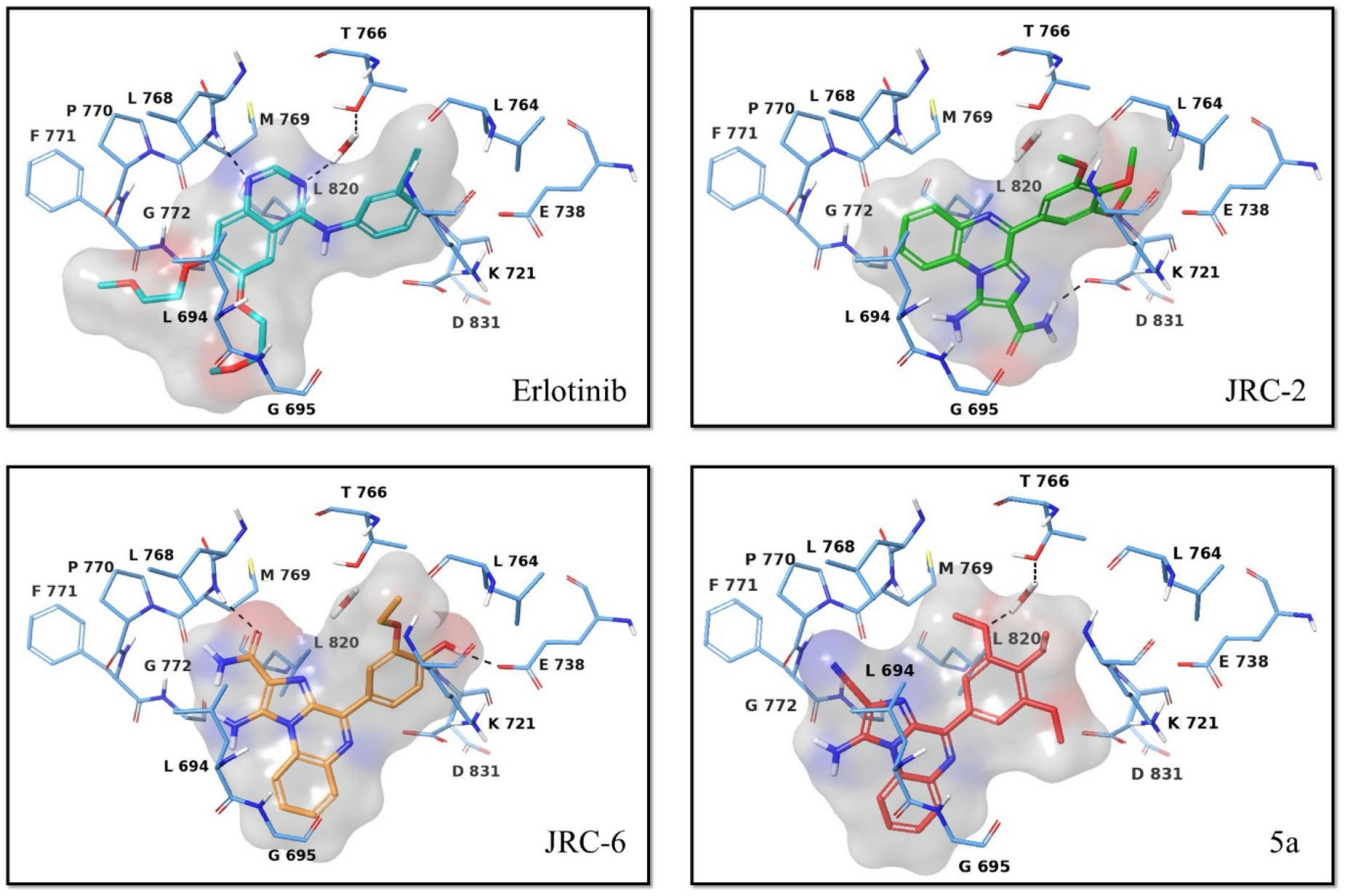
Docking pose of investigational compounds **JRC-2**, **JRC-6,** and **5a**, relative to erlotinib, at the ATP-binding site of EGFR (**PDB ID: 1M17**).

Molecular docking studies were further performed at the EGFR binding site (**PDB ID: 1M17**) to investigate the binding mode and protein-ligand interactions of **JRC-2** and **JRC-6** in comparison to the positive control erlotinib. Both compounds **JRC-2** and **JRC-6,** along with **5a** and erlotinib, successfully occupied the ATP-binding site of wild-type EGFR. The docking score of erlotinib was found to be −9.2 kcal/mol, while investigational compounds showed a docking score of −6.2 kcal/mol, −6.8 kcal/mol, and −6.6 kcal/mol, respectively, for **5a, JRC-2,** and **JRC-6** (Figure 8). The carboxamide unit of compound **JRC-2** H-bonds to the D831, while the same unit of compounds **JRC-6** interacts with M769, at the ATP-binding site of EGFR. Because of the nitrile substitution in compound **5a**, no such interactions were detected. Despite showing docking scores in the reasonable range, the investigational compounds **JRC-2** and **JRC-6** do not form a key interaction, i.e. a water-mediated H-bond with T766 at the EGFR’s ATP-binding site, which is shown by the reference drug erlotinib, and is believed to contribute to overall binding affinity. This further reflects on significantly higher docking scores and potent biological activity of erlotinib against EGFR, relative to the investigational compounds **JRC-2** and **JRC-6**.

**Figure 9.**
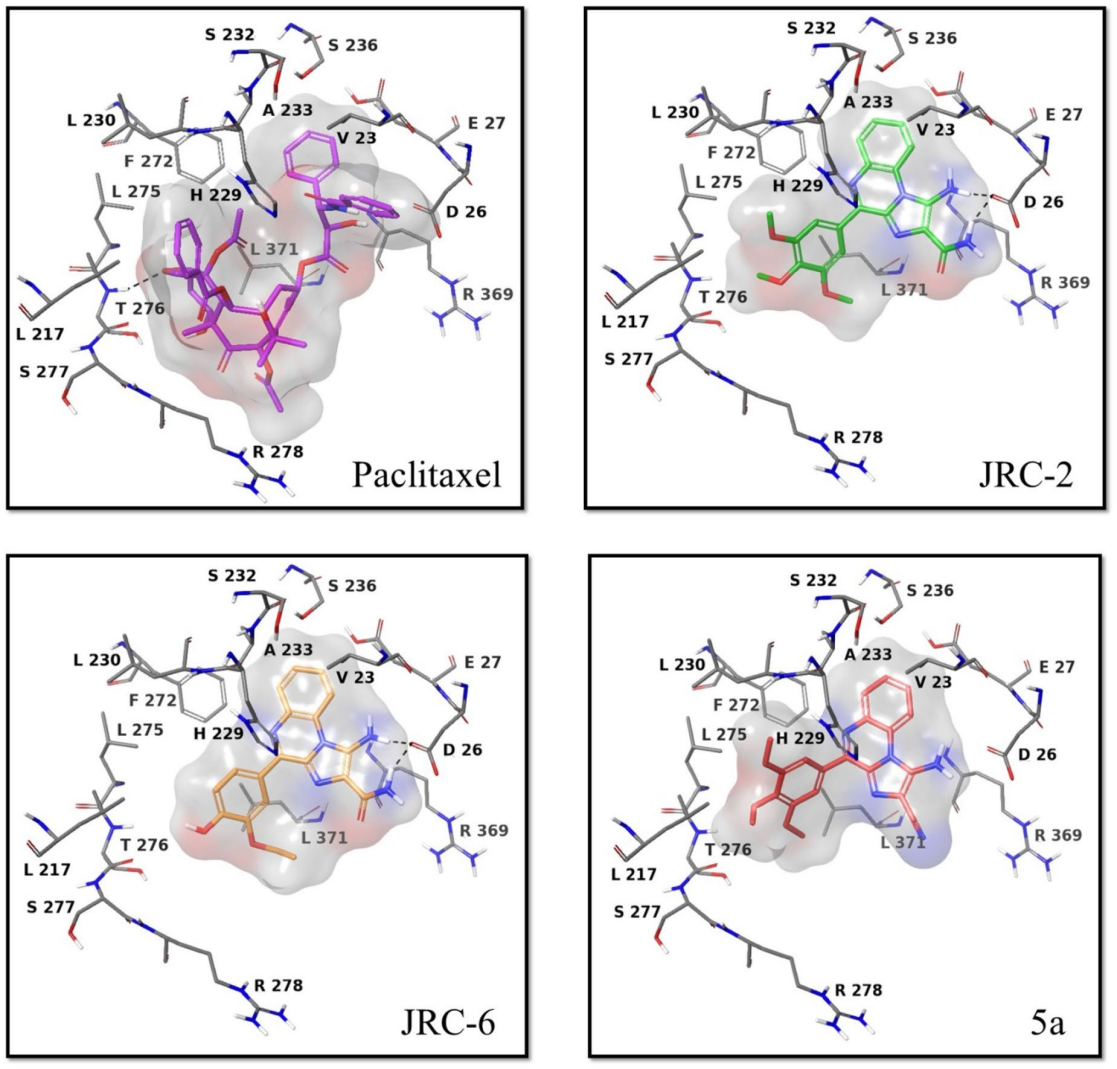
Docking pose of investigational compounds **JRC-2**, **JRC-6,** and **5a**, relative to paclitaxel, at the taxol-binding site of tubulin (**PDB ID: 1JFF**).

We furthermore predicted the binding mode of the investigational compounds **JRC-2**, **JRC-6,** and **5a** at the taxol-binding site of tubulin (**PDB: 1JFF**), using paclitaxel as a reference drug. The investigational compounds **JRC-2**, **JRC-6,** and **5a** bind to the taxol-binding site on tubulin, similar to paclitaxel. The standard inhibitor, paclitaxel, has a docking energy of −8.3 kcal/mol. At the same time, investigational compounds **5a, JRC-3**, and **JRC-7** showed docking scores of −5.8 kcal/mol, −6.7 kcal/mol, and −6.9 kcal/mol, respectively, confirming a reasonable binding at the taxol-binding site of tubulin (Figure 9). The amino-group and carboxamide unit of both compounds **JRC-2** and **JRC-6** are involved in H-bond contact with D26, which is believed to contribute to the binding affinity for the target. No promising interaction was observed for compound **5a**, which aligns with its cytotoxicity data and decreased cytotoxicity.

As the investigational compound **JRC-6** was the most potent inhibitor in the series, as observed from cell viability results, and it also stabilised tubulin dynamics, we further investigated the tubulin–**JRC-6** interaction by conducting a 100 ns molecular dynamics (MD) simulation. The RMSD (root mean square deviation) of the ligand atoms and the α-carbons of the protein was calculated throughout the simulation as a measure of ligand and protein mobility, respectively. Both tubulin and compound **JRC-6** showed decent stability over 100 ns simulations, with average RMSDs of 1.95 Å and 4.30 Å, respectively, as shown in the RMSD plot. As seen in the docking pose, the H-bond between compound JRC-6 and D26 in the taxol-binding site is maintained for ∼80% of the simulation time, confirming a strong interaction. Interestingly, the hydroxy group of compound **JRC-6** H-bonds to T276 of tubulin, which was not observed with the docking pose. The said interaction is also observed between the standard drug paclitaxel and the taxol-binding site of tubulin; it can thus be considered as a key interaction. The H-bond interaction between compound **JRC-6** and tubulin accounts for ∼80% of the simulation trajectory, confirming its contribution towards the binding affinity and investigated biological activity. Furthermore, the RMSD plot shows fluctuations in compound **JRC-6** during the first 20 ns, corresponding to conformational changes/rotation of the 2-ethoxyphenol ring of **JRC-6**, which interacts with T276. Finally, the imidazole ring of the tricyclic ring system of compound JRC-6 forms pi-pi stacking with the H229 of tubulin for ∼75% of the simulation time, again indicating stronger binding. The MD analysis thus confirms the stable binding and persistent interactions of the investigational compound **JRC-6** at the taxol-binding site of tubulin, which is in agreement with the observed biological activity.

**Figure 10.**
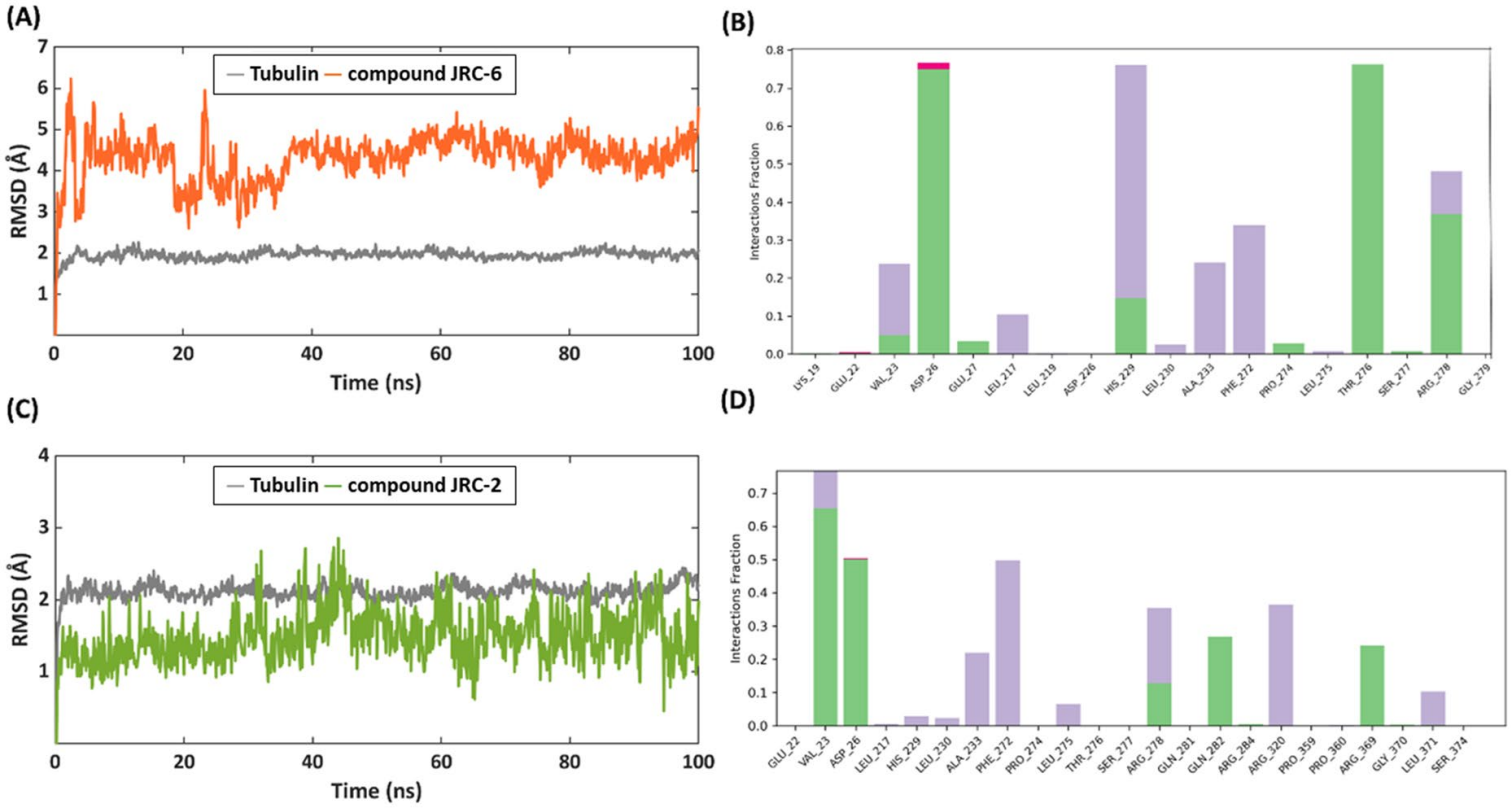
RMSD plot from the 100 ns MD simulations of (A) **Tubulin –** compound **JRC-6** complex, (C) **Tubulin –** compound **JRC-2** complex. Tubulin α-carbons, **JRC-6** and **JRC-2,** are coloured grey and orange, respectively. Protein–ligand histogram from the MD simulation analysis of (B) compound **JRC-6**, (D) compound **JRC-2**, at the taxol-binding site of tubulin. H-bond interactions, lipophilic contacts, and salt-bridge interactions are coloured in green, grey, and pink, respectively.

To perform a comparative analysis, we also carried out 100 ns MD simulations of the investigational compound **JRC-2** bound to tubulin, which was less effective than compound **JRC-6** at stabilising tubulin dynamics. Both the protein and ligand atoms of the tubulin–**JRC-6** complex demonstrated remarkable stability over the 100 ns simulation, with average RMSD values of 2.11 Å and 1.48 Å, respectively, as shown in the RMSD plot (Figure 10). Despite its high stability, compound **JRC-2** fails to exhibit the essential protein–ligand interactions at the taxol-binding site of tubulin, as demonstrated by the standard drug paclitaxel and compound **JRC-6,** as discussed above. The carboxamide unit of compound **JRC-2** forms H-bonds with D26 for 50% of the simulation time, while compound **JRC-6** engages for 80% of the simulation trajectory. No H-bond or lipophilic contact with the other key residues, such as H229 and T276 of tubulin’s taxol binding site, was formed. It can thus be hypothesised that compound **JRC-2** lacks persistence of interactions with the key residues (H229 and T276) at the taxol-binding site of tubulin, which can be correlated with its observed, relatively poor tubulin stabilisation from the experiments, where compound **JRC-6** and paclitaxel consistently maintain the interactions with the key residues of tubulin, leading to enhanced biochemical activity.

## Conclusion

In conclusion, we demonstrated the potential of bioisosteric replacement of a nitrile with a carboxamide, yielding promising lead compounds comprising a series of novel carboxamide-fused imidazo[1,2-a]quinoxalines as dual-targeting agents for EGFR and tubulin. Furthermore, DFT calculations revealed intramolecular hydrogen bonding in the carboxamide intermediate, rationalising the regioselectivity of the Pictet–Spengler reaction and the favourable reaction energetics. Comparative *in vitro* cytotoxicity results indicated that **JRC-2** exhibits superior activity (IC_50_ = 4.59 µM against MCF-7) compared with its corresponding nitrile analogue, compound **5a** (IC_50_ = 19.6 µM against MCF-7). Compounds **JRC-2** and **JRC-6** inhibited wild-type EGFR with nanomolar potency (IC_50_ = 294.45 nM and 383.90 nM, respectively), while **JRC-6** additionally demonstrated superior microtubule-stabilising activity, approaching that of paclitaxel, and induced ROS-mediated mitochondrial dysfunction and G2/M phase cell cycle arrest. Molecular docking and 100 ns molecular dynamics simulations corroborated the experimental findings, revealing stable, persistent interactions of **JRC-6** at the EGFR ATP-binding site and the taxol-binding site of tubulin, whereas weaker interaction persistence accounted for the lower tubulin activity of **JRC-2**. Collectively, these results establish **JRC-2** and **JRC-6** as promising anticancer leads and warrant further *in vitro* and in vivo investigations of their mechanism of action, in addition to EGFR/tubulin-targeting, and underscore the value of carboxamide substitution combined with Pictet–Spengler chemistry for the development of multi-targeted anticancer agents.

## Experimental section

### General information

All the reactions were performed using standard laboratory glassware, equipment, and chemicals. A few of the regularly used equipment include a weighing balance (Mettler Toledo), a rotary evaporator (IKA), a heating mantle cum stirrer (IKA), a digital hot plate (IKA), and a vacuum oven. Commercially available reagents and solvents were obtained from various suppliers, including TCI Chemicals, Sigma-Aldrich, GLR Innovations, and Rankem. Reaction progress was monitored using analytical-grade, commercially available, precoated TLC plates (Merck silica gel 60 F_254_; ethyl acetate/hexane) and visualised under UV or in an iodine chamber. For analytical purposes, HRMS was acquired on an Agilent QTOF-6530 LC/MS, and NMR was analysed on a JEOL NMR spectrometer at the Central University of Punjab.

### General procedure for the synthesis of target compounds

The initial step involves the reaction of diaminomaleonitrile (**1**) (3 g, 18.50 mmol) with triethyl orthoformate (3.0 ml) in 1,4-dioxane (25 ml). The reaction mixture was heated to 60–80 °C for 6h, and upon completion of the reaction, the volatiles were removed under reduced pressure using a rotary evaporator. The concentrated mixture was further extracted with 100 ml of diethyl ether, and after crystallisation, crystals of ethyl N-((*Z*)-2-amino-1,2-dicyanovinyl)formimidate (**2**) were obtained. These crystals were then reacted with orthophenylene diamine in ethanol, in the presence of a catalytic amount of aniline hydrochloride. The following step was conducted at room temperature, and after removing the volatiles under reduced pressure, N’-((*Z*)-2-amino-1,2-dicyanovinyl)-N-formimidamide (**3**) was obtained as a solid powder. This was further treated with 1 M aqueous KOH at room temperature to afford 5-amino-1–(2-aminophenyl)-1*H*-imidazole-4-carbonitrile (**4**). The crude reaction mixture was filtered through a Buchner funnel, and excess base was removed through washing with sufficient water. This stable intermediate **4** was further subjected to base-catalyzed hydrolysis to afford the formation of 5-amino-1–(2-aminophenyl)-1*H*-imidazole-4-carboxamide (**JR**). This step was performed under controlled reaction conditions using 50% aqueous NaOH, with the reaction temperature maintained at 40–50 °C and the reaction progress monitored by TLC to ensure complete consumption of **4**.

*5-Amino-1–(2-aminophenyl)-1H-imidazole-4-carboxamide* **(JR):** Yield 58%, Appearance: yellow solid, (EA/Hexane = 80%, R*_f_* = 0.1).**^1^H-NMR (600 MHz, DMSO-*d*_6_) δ** 7.19 (t, J = 8.4 Hz, 1H), 7.13 (s, 1H), 7.03 (d, J = 6.4 Hz, 1H), 6.89 (d, J = 7.0 Hz, 2H), 6.71–6.66 (m, 2H), 5.45 (s, 2H), 5.06 (s, 2H).**^13^C-NMR (151 MHz, DMSO-*d*_6_) δ** 166.7, 144.3, 143.1, 130.0, 129.8, 128.1, 119.1, 116.7, 116.4, 112.5. **MS (EI) m/z**: 217.05.

#### Synthesis of compound JRC-1 to JRC-18

Compound **JR** (0.46 mmol) was further reacted with different substituted benzaldehydes (0.46 mmol) or acetophenones (0.46 mmol) at an equimolar amount in 1 ml of methanol at 60–80 °C in the presence of conc. H_2_SO_4_ (1 drop) and after the completion of the reaction (TLC monitored), the volatiles were evaporated. The crude reaction mixture was further washed with a saturated bicarbonate solution and then concentrated under reduced pressure to yield the desired product in high yield.

*1-Amino-4–(3,4-dimethoxyphenyl)imidazo[1,2-a]quinoxaline-2-carboxamide* (**JRC-1**): Yield 81%, Appearance: yellow solid, (EA/Hexane = 70%, R*_f_* = 0.3). **^1^H-NMR (600 MHz, DMSO-*d*_6_) δ** 8.67 (dd, J = 8.5, 1.8 Hz, 1H), 8.56–8.54 (m, 1H), 8.32 (d, J = 1.8 Hz, 1H), 8.01–8.00 (m, 1H), 7.63–7.59 (m, 3H) (-NH), 7.46 (s, 1H) (-NH), 7.10 (d, J = 8.6 Hz, 1H), 6.72 (s, 2H) (-NH_2_), 3.91 (s, 3H), 3.87 (s, 3H). **^13^C-NMR (151 MHz, DMSO-*d*_6_) δ** 166.8, 151.1, 148.4, 148.2, 140.8, 135.8, 129.6, 129.3, 128.0, 127.5, 126.9, 126.4, 124.1, 118.5, 115.7, 112.1, 110.9, 55.5, 55.5. **HRMS (ESI-TOF) m/z [M + H]^+^** Calcd for Chemical Formula: C_19_H_18_N_5_O_3_^+^ 364.1409; observed 364.1398.

*1-Amino-4–(3,4,5-trimethoxyphenyl)imidazo[1,2-a]quinoxaline-2-carboxamide* (**JRC-2**): Yield 87%, Appearance: yellow solid, (EA/Hexane = 70%, R*_f_* = 0.3). **^1^H-NMR (600 MHz, DMSO-*d*_6_) δ** 8.56 (d, J = 8.0 Hz, 1H), 8.05–8.01 (m, 3H), 7.65–7.60 (m, 2H), 7.45 (d, J = 10.7 Hz, 2H), 6.75 (s, 2H), 3.93 (s, 6H), 3.77 (s, 3H). **^13^C-NMR (151 MHz, DMSO-*d*_6_) δ** 166.7, 152.4, 148.8, 141.0, 139.7, 135.7, 130.8, 129.6, 129.4, 127.6, 127.3, 126.5, 118.6, 115.7, 107.2, 60.2, 56.0. **HRMS (ESI-TOF) m/z [M + H]^+^** Calcd for Chemical Formula: C_20_H_20_N_5_O_4_^+^ 394.1515; observed 394.1507.

*1-Amino-4–(4-hydroxy-3-methoxyphenyl)imidazo[1,2-a]quinoxaline-2-carboxamide* (**JRC-3**): Yield 91%, Appearance: yellow solid, (EA/Hexane = 70%, R*_f_* = 0.2). **^1^H-NMR (600 MHz, DMSO-*d*_6_) δ** 9.64 (s, 1H), 8.58 (dd, J = 8.4, 1.9 Hz, 1H), 8.54 (t, J = 4.8 Hz, 1H), 8.34 (d, J = 1.8 Hz, 1H), 8.00–7.98 (m, 1H), 7.63 (s, 1H), 7.61–7.58 (m, 2H), 7.45 (s, 1H), 6.95 (d, J = 8.6 Hz, 1H), 6.70 (s, 2H), 3.92 (s, 3H). **^13^C-NMR (151 MHz, DMSO-*d*_6_) δ** 166.8, 149.4, 148.5, 147.1, 140.8, 135.9, 129.6, 129.1, 127.4, 126.7, 126.3, 124.5, 118.4, 115.6, 115.1, 112.9, 55.6. **HRMS (ESI-TOF) m/z [M + H]^+^** Calcd for Chemical Formula: C_18_H_16_N_5_O_3_^+^ 350.1247; observed 350.1254.

*1-Amino-4–(4-hydroxyphenyl)imidazo[1,2-a]quinoxaline-2-carboxamide* (**JRC-4**): Yield 93%, Appearance: orange solid, (EA/Hexane = 80%, R*_f_* = 0.2). **^1^H-NMR (600 MHz, DMSO-*d*_6_) δ** 10.00 (s, 1H), 8.82 (d, J = 7.2 Hz, 2H), 8.54–8.53 (m, 1H), 7.97–7.96 (m, 1H), 7.75 (s, 1H), 7.60–7.58 (m, 2H), 7.45 (s, 1H), 6.93 (d, J = 7.2 Hz, 2H), 6.69 (s, 2H). **^13^C-NMR (151 MHz, DMSO-*d*_6_) δ** 166.8, 159.9, 148.5, 140.8, 136.0, 131.8, 129.5, 129.1, 127.4, 126.7, 126.4, 126.4, 118.4, 115.6, 115.0. **HRMS (ESI-TOF) m/z [M + H]^+^** Calcd for Chemical Formula: C_17_H_14_N_5_O_2_^+^ 320.1141; observed 320.1142.

*1-Amino-4–(3,4-bis(2-methoxyethoxy)phenyl)imidazo[1,2-a]quinoxaline-2-carboxamide* (**JRC-5):** Yield 77%, Appearance: orange solid, (EA/Hexane = 80%, R*_f_* = 0.1). **^1^H-NMR (600 MHz, DMSO-*d*_6_) δ** 8.64 (dd, J = 8.7, 2.1 Hz, 1H), 8.56–8.54 (m, 1H), 8.35 (d, J = 2.2 Hz, 1H), 8.01–8.00 (m, 1H), 7.64–7.59 (m, 3H), 7.48 (s, 1H), 7.13 (d, J = 8.7 Hz, 1H), 6.72 (s, 2H), 4.23 (dt, J = 21.6, 4.6 Hz, 4H), 3.73 (dd, J = 9.2, 5.7 Hz, 4H), 3.35 (d, J = 3.9 Hz, 6H). **^13^C-NMR (151 MHz, DMSO-*d*_6_) δ** 166.8, 150.7, 148.3, 147.6, 140.9, 135.9, 129.6, 129.3, 128.3, 127.5, 127.0, 126.5, 124.5, 118.5, 115.7, 114.6, 112.8, 70.5, 70.4, 68.1, 67.9, 58.4. **HRMS (ESI-TOF) m/z [M + H]^+^** Calcd for Chemical Formula: C_23_H_26_N_5_O_5_^+^ 452.1934; observed 452.1939.

*1-Amino-4–(3-ethoxy-4-hydroxyphenyl)imidazo[1,2-a]quinoxaline-2-carboxamide* (**JRC-6**): Yield 92%, Appearance: orange solid, (EA/Hexane = 80%, R*_f_* = 0.2). **^1^H-NMR (600 MHz, DMSO-*d*_6_) δ** 9.57 (s, 1H), 8.57–8.53 (m, 2H), 8.30 (s, 1H), 7.98 (t, J = 3.2 Hz, 1H), 7.59 (d, J = 3.7 Hz, 3H), 7.45 (s, 1H), 6.96 (d, J = 9.1 Hz, 1H), 6.70 (s, 2H), 4.18 (dd, J = 12.7, 6.1 Hz, 2H), 1.41 (t, J = 6.5 Hz, 3H). **^13^C-NMR (151 MHz, DMSO-*d*_6_) δ** 166.8, 149.7, 148.6, 146.3, 140.8, 135.9, 129.6, 129.2, 127.4, 126.7, 126.4, 124.5, 118.4, 115.6, 115.1, 114.2, 63.9, 14.9. **HRMS (ESI-TOF) m/z [M + H]^+^** Calcd for Chemical Formula: C_19_H_18_N_5_O_3_^+^ 364.1409; observed 364.1417.

*1-Amino-4–(4-bromophenyl)imidazo[1,2-a]quinoxaline-2-carboxamide* (**JRC-7**): Yield 79%, Appearance: orange solid, (EA/Hexane = 70%, R*_f_* = 0.3). **^1^H-NMR (600 MHz, DMSO-*d_6_*) δ** 8.84 (d, J = 8.7 Hz, 2H), 8.57 (d, J = 8.3 Hz, 1H), 8.04 (dd, J = 7.8, 1.7 Hz, 1H), 7.81 (s, 1H), 7.75 (d, J = 8.7 Hz, 2H), 7.69–7.63 (m, 2H), 7.51 (s, 1H), 6.78 (s, 2H). **^13^C-NMR (151 MHz, DMSO-*d_6_*) δ** 166.7, 147.8, 141.0, 135.7, 134.6, 131.9, 131.2, 129.6, 129.5, 127.8, 127.7, 126.6, 124.4, 118.8, 115.8. **HRMS (ESI-TOF) m/z [M + H]^+^** Calcd for Chemical Formula: C_17_H_13_BrN_5_O^+^ 382.0303; observed 382.0306.

*1-Amino-4–(4-isopropylphenyl)imidazo[1,2-a]quinoxaline-2-carboxamide* (**JRC-8**): Yield 84%, Appearance: yellow solid, (EA/Hexane = 70%, R*_f_* = 0.3). **^1^H-NMR (600 MHz, DMSO-*d_6_*) δ** 8.74 (d, J = 8.3 Hz, 2H), 8.57 (dd, J = 8.2, 1.3 Hz, 1H), 8.03 (dd, J = 7.7, 1.8 Hz, 1H), 7.73 (s, 1H), 7.67–7.62 (m, 2H), 7.47 (s, 1H), 7.44 (d, J = 8.5 Hz, 2H), 6.75 (s, 2H), 3.05–2.98 (m, 1H), 1.29 (s, 3H), 1.28 (s, 3H). **^13^C-NMR (151 MHz, DMSO-*d_6_*) δ** 166.8, 151.0, 149.2, 140.9, 136.0, 133.3, 130.0, 129.7, 129.5, 127.7, 127.3, 126.5, 126.2, 118.7, 115.8, 33.5, 23.8. **HRMS (ESI-TOF) m/z [M + H]^+^** Calcd for Chemical Formula: C_20_H_20_N_5_O^+^ 346.1668; observed 346.1674.

*1-Amino-4–(4-cyanophenyl)imidazo[1,2-a]quinoxaline-2-carboxamide* (**JRC-9**): Yield 83%, Appearance: yellow solid, (EA/Hexane = 70%, R*_f_* = 0.3). **^1^H-NMR (600 MHz, DMSO-*d_6_*) δ** 9.02 (d, J = 8.5 Hz, 2H), 8.56 (dd, J = 8.3, 1.0 Hz, 1H), 8.04 (dd, J = 7.9, 1.5 Hz, 1H), 7.98 (d, J = 8.7 Hz, 2H), 7.83 (s, 1H), 7.70–7.67 (m, 1H), 7.65–7.62 (m, 1H), 7.50 (s, 1H), 6.79 (s, 2H). **^13^C-NMR (151 MHz, DMSO-*d_6_*) δ** 166.7, 147.1, 141.0, 139.6, 135.7, 132.1, 130.5, 129.8, 129.5, 128.2, 127.9, 126.7, 118.9, 115.9, 112.6. **HRMS (ESI-TOF) m/z [M + H]^+^** Calcd for Chemical Formula: C_18_H_13_N_6_O^+^ 329.1151; observed 329.1151.

*4–(4-(Allyloxy)phenyl)-1-aminoimidazo[1,2-a]quinoxaline-2-carboxamide* (**JRC-10**): Yield 91%, Appearance: yellow solid, (EA/Hexane = 70%, R*_f_* = 0.3). **^1^H-NMR (600 MHz, CDCl_3_) δ** 8.62 (d, J = 8.9 Hz, 2H), 8.36 (d, J = 8.1 Hz, 1H), 8.07 (dd, J = 7.7, 1.5 Hz, 1H), 7.56–7.51 (m, 2H), 7.07–7.04 (m, 3H), 6.10 (dq, J = 22.5, 5.3 Hz, 1H), 5.63 (s, 2H), 5.49–5.45 (m, 2H), 5.33 (dd, J = 10.5, 1.4 Hz, 1H), 4.64 (d, J = 5.2 Hz, 2H). **^13^C-NMR (151 MHz, CDCl_3_) δ** 167.2, 160.9, 150.4, 140.4, 136.9, 133.1, 131.6, 131.1, 130.5, 128.7, 128.1, 127.3, 126.7, 119.4, 118.1, 114.6, 69.0. **HRMS (ESI-TOF) m/z [M + H]^+^** Calcd for Chemical Formula: C_20_H_18_N_5_O_2_^+^ 360.1460; observed 360.1462.

*1-Amino-4–(4-(prop-2-yn-1-yloxy)phenyl)imidazo[1,2-a]quinoxaline-2-carboxamide* (**JRC-11**): Yield 89%, Appearance: orange solid, (EA/Hexane = 70%, R*_f_* = 0.3). **^1^H-NMR (600 MHz, DMSO-*d_6_*) δ** 8.90 (d, J = 8.7 Hz, 2H), 8.54 (d, J = 8.5 Hz, 1H), 7.99 (d, J = 8.9 Hz, 1H), 7.78 (s, 1H), 7.63–7.59 (m, 2H), 7.44 (s, 1H), 7.15 (d, J = 8.7 Hz, 2H), 6.71 (s, 2H), 4.92 (s, 2H), 3.63 (s, 1H). **^13^C-NMR (151 MHz, DMSO-*d_6_*) δ** 166.8, 159.2, 148.3, 140.9, 135.9, 131.6, 129.6, 129.3, 128.7, 127.6, 127.1, 126.5, 118.6, 115.7, 114.4, 79.1, 78.6, 55.6. **HRMS (ESI-TOF) m/z [M + H]^+^** Calcd for Chemical Formula: C_20_H_16_N_5_O_2_^+^ 358.1304; observed 358.1303.

*1-Amino-4–(3-hydroxyphenyl)-4-methyl-4,5-dihydroimidazo[1,2-a]quinoxaline-2-carboxamide* (**JRC-12**): Yield 94%, Appearance: orange solid, (EA/Hexane = 90%, R*_f_* = 0.2). **^1^H-NMR (600 MHz, DMSO-*d*_6_) δ** 9.32 (s, 1H), 7.65 (d, J = 8.1 Hz, 1H), 7.25 (s, 1H), 7.06–6.99 (m, 3H), 6.95 (d, J = 20.6 Hz, 2H), 6.75–6.72 (m, 2H), 6.58 (s, 1H), 6.51 (dd, J = 8.1, 2.3 Hz, 1H), 6.05 (s, 2H), 1.82 (s, 3H). **^13^C-NMR (151 MHz, DMSO-*d*_6_) δ** 165.7, 157.2, 146.1, 141.9, 139.3, 137.3, 129.3, 126.9, 122.6, 118.4, 117.1, 116.1, 115.9, 114.0, 112.5, 57.0, 28.5. **HRMS (ESI-TOF) m/z [M + H]^+^** Calcd for Chemical Formula: C_18_H_18_N_5_O_2_^+^ 336.1460; observed 336.1465.

*1-Amino-4–(2,4-dimethoxyphenyl)-4-methyl-4,5-dihydroimidazo[1,2-a]quinoxaline-2-carboxamide* **(JRC-13):** Yield 80%, Appearance: orange solid, (EA/Hexane = 90%, R*_f_* = 0.2). **^1^H-NMR (600 MHz, DMSO-*d*_6_) δ** 7.62 (d, J = 8.1 Hz, 1H), 7.23 (s, 1H), 7.04–6.99 (m, 4H), 6.90 (s, 1H), 6.72 (d, J = 8.3 Hz, 2H), 6.51 (dd, J = 8.5, 1.9 Hz, 1H), 6.04 (s, 2H), 3.64 (s, 3H), 3.62 (s, 3H), 1.82 (s, 3H). **^13^C-NMR (151 MHz, DMSO-*d*_6_) δ** 167.1, 148.5, 147.7, 141.8, 139.9, 137.7, 137.4, 126.2, 123.4, 118.3, 117.7, 116.6, 116.0, 113.2, 111.2, 109.5, 56.9, 55.5, 55.4, 28.7. **HRMS (ESI-TOF) m/z [M + H]^+^** Calcd for Chemical Formula: C_20_H_22_N_5_O_3_^+^ 380.1722; observed 380.1724.

*1-Amino-4–(3-methoxyphenyl)-4-methyl-4,5-dihydroimidazo[1,2-a]quinoxaline-2-carboxamide* **(JRC-14):** Yield 80%, Appearance: orange solid, (EA/Hexane = 90%, R*_f_* = 0.2). **^1^H-NMR (600 MHz, DMSO-*d*_6_) δ** 7.64 (d, J = 8.1 Hz, 1H), 7.31 (s, 1H), 7.13 (t, J = 7.9 Hz, 1H), 7.07–7.03 (m, 2H), 7.00 (s, 1H), 6.94 (s, 1H), 6.86 (s, 1H), 6.76–6.73 (m, 2H), 6.71 (dd, J = 8.2, 2.4 Hz, 1H), 6.05 (s, 2H), 3.66 (s, 3H), 1.84 (s, 3H). **^13^C-NMR (151 MHz, DMSO-*d*_6_) δ** 166.7, 159.2, 146.9, 141.8, 139.5, 137.2, 129.4, 126.5, 123.1, 118.4, 117.7, 116.7, 116.0, 112.8, 111.9, 111.6, 57.1, 55.0, 28.6. **HRMS (ESI-TOF) m/z [M + H]^+^** Calcd for Chemical Formula: C_19_H_20_N_5_O_2_^+^ 350.1617; observed 350.1614.

*1-Amino-4–(3,4-dimethoxyphenyl)-4-methyl-4,5-dihydroimidazo[1,2-a]quinoxaline-2-carboxamide* **(JRC-15):** Yield 81%, Appearance: orange solid, (EA/Hexane = 90%, R*_f_* = 0.2). **^1^H-NMR (600 MHz, DMSO-*d*_6_) δ** 7.64 (d, J = 8.1 Hz, 1H), 7.26 (s, 1H), 7.06–7.03 (m, 3H), 7.01 (s, 1H), 6.93 (s, 1H), 6.76–6.73 (m, 2H), 6.53 (dd, J = 8.5, 2.1 Hz, 1H), 6.06 (s, 2H), 3.66 (s, 3H), 3.64 (s, 3H), 1.84 (s, 3H). **^13^C-NMR (151 MHz, DMSO-*d*_6_) δ** 167.1, 148.5, 147.6, 141.7, 139.9, 137.7, 137.4, 126.2, 123.4, 118.3, 117.7, 116.6, 116.0, 113.2, 111.1, 109.4, 57.0, 55.5, 55.4, 28.7 **HRMS (ESI-TOF) m/z [M + H]^+^** Calcd for Chemical Formula: C_20_H_22_N_5_O_3_^+^ 380.1722; observed 380.1739.

*1-Amino-4-methyl-4–(3-nitrophenyl)-4,5-dihydroimidazo[1,2-a]quinoxaline-2-carboxamide* **(JRC-16):** Yield 77%, Appearance: orange solid, (EA/Hexane = 90%, R*_f_* = 0.2). **^1^H-NMR (600 MHz, DMSO-*d*_6_) δ** 8.16 (s, 1H), 8.02–8.00 (m, 1H), 7.65 (d, J = 7.8 Hz, 2H), 7.56–7.54 (m, 2H), 7.10–7.05 (m, 2H), 7.03 (s, 1H), 6.97 (s, 1H), 6.76 (t, J = 7.5 Hz, 1H), 6.09 (s, 2H), 1.88 (s, 3H). **^13^C-NMR (151 MHz, DMSO-*d*_6_) δ** 166.9, 147.9, 147.7, 141.9, 138.6, 136.5, 132.2, 130.0, 126.5, 123.1, 122.2, 120.1, 118.8, 116.7, 116.0, 113.5, 57.1, 28.3. **HRMS (ESI-TOF) m/z [M + H]^+^** Calcd for Chemical Formula: C_18_H_17_N_6_O_3_^+^ 365.1362; observed 365.1370.

*1-Amino-4–(4-hydroxyphenyl)-4-methyl-4,5-dihydroimidazo[1,2-a]quinoxaline-2-carboxamide* (**JRC-17**): Yield 91%, Appearance: orange solid, (EA/Hexane = 90%, R*_f_* = 0.2). **^1^H-NMR (600 MHz, DMSO-*d_6_*) δ** 9.30 (s, 1H), 7.62 (d, J = 8.1 Hz, 1H), 7.16 (s, 1H), 7.03–6.99 (m, 4H), 6.95 (s, 1H), 6.90 (s, 1H), 6.73–6.70 (m, 1H), 6.73–6.70 (m, 1H), 6.56 (d, J = 8.7 Hz, 2H), 6.02 (s, 2H), 1.78 (s, 3H). **^13^C-NMR (151 MHz, DMSO-*d_6_*) δ** 167.1, 156.2, 141.7, 140.0, 137.4, 135.5, 126.5, 126.2, 123.3, 118.1, 116.5, 115.9, 114.8, 113.2, 56.7, 28.9. **HRMS (ESI-TOF) m/z [M + H]^+^** Calcd for Chemical Formula: C_18_H_18_N_5_O_2_^+^ 336.1460; observed 336.1458.

*(E)-1-amino-4-methyl-4-styryl-4,5-dihydroimidazo[1,2-a]quinoxaline-2-carboxamide* (**JRC-18**): Yield 85%, Appearance: orange solid, (EA/Hexane = 90%, R*_f_* = 0.2). **^1^H-NMR (600 MHz, DMSO-*d_6_*) δ** 7.75 (d, J = 8.1 Hz, 1H), 7.24 (s, 4H), 7.18 (s, 1H), 7.06 (t, J = 7.5 Hz, 1H), 7.01 (d, J = 7.8 Hz, 1H), 6.89 (s, 2H), 6.79 (t, J = 7.6 Hz, 1H), 6.75 (s, 1H), 6.27 (d, J = 16.1 Hz, 1H), 6.13 (d, J = 15.9 Hz, 1H), 6.07 (s, 2H), 1.72 (s, 3H). **^13^C-NMR (151 MHz, DMSO-*d_6_*) δ** 166.9, 141.7, 139.0, 137.0, 135.8, 133.5, 128.7, 127.7, 126.3, 126.2, 122.9, 118.3, 116.5, 115.8, 113.5, 55.3, 25.6. **HRMS (ESI-TOF) m/z [M + H]^+^** Calcd for Chemical Formula: C_20_H_20_N_5_O^+^ 346.1668; observed 346.1668.

### In vitro anticancer evaluation

All investigational cancer cell lines, including A549, MCF-7, MDA-MB-231, and the non-cancerous cell line HEK-293, were purchased from the NCCS, Pune, India. The cells were cultured in a sterile, precoated T25 flask using freshly prepared culture media. The culture media was composed of 1% penicillin–streptomycin and 10% FBS in commercially purchased DMEM. An ambient cellular growth condition was maintained in a CO2 incubator at 37 °C with a constant 5% CO_2_ supply. When the cellular population in T25 flasks reached 70–80% confluency, cells were subcultured into 96-well plates, 100 mm culture dishes, or 6-well plates for various anticancer assays.

#### Cell viability assay

All the cells under investigation were seeded in a precoated 96-well tissue culture plate using culture media and allowed to adhere overnight at 37 °C in a humidified 5% CO_2_ incubator until 70% confluency was achieved. For initial screening, cells were treated with all the synthesised compounds, and erlotinib was used as a positive control at a single concentration of 10 µM for 24h. After 24 h of treatment, cells were washed with 1X PBS (pH 7.4) and cell viability was assessed using an MTT-based assay. In each well, 100 µL of 0.5 mg/mL MTT in PBS was added, and the plates were incubated for an additional 4 h at 37 °C. Excess MTT was removed, and formazan was dissolved in 100–200 µL of biological-grade DMSO. Gentle shaking was performed on an orbital shaker for 10–15 min, protected from light. Absorbance was recorded at 570 nm using a microplate reader, and IC_50_ was calculated by non-linear regression analysis using GraphPad Prism software.

#### EGFR enzymatic assay

The enzymatic inhibitory potential of the potent compounds against EGFR was evaluated using a commercially available enzymatic assay kit (Invitrogen, Thermo Fisher Scientific, Cat. Nos. PV3193 and PV3872). The assay was performed strictly according to the manufacturer’s protocol to ensure reproducibility. All components of the assay procedure were prepared freshly at the respective working concentrations. 1X kinase buffer was prepared from the supplied 5X kinase buffer using distilled water and used to prepare the 2X kinase solution; however, vortexing should be avoided at this stage. In the next step, a Tyr 4 peptide-ATP mixture was prepared containing 2X ATP relative to the desired final concentration, using Tyr 4 peptide, ATP, and 1X kinase buffer. Tyr 4 phosphopeptide was further prepared by mixing it with 1X kinase buffer. Similarly, the development solution was prepared by mixing development reagent B with development buffer B at the ratio specified in the protocol. All reagent volumes were calculated based on a low-volume 384-well non-adherent opaque microplate with a 20 µL reaction capacity in each well. The reaction wells were grouped accordingly, as required, into 0% phosphorylation control, 100% phosphorylation control, 0% inhibition control, and test groups. In the 0% phosphorylation control, 1X kinase buffer and the peptide-ATP mixture were added, and after gentle shaking, the plate was incubated for 1 h at 20–25 °C. Similarly, in the 100% phosphorylation control, 1X kinase buffer and phosphopeptide mixture were added, and after gentle shaking, the plate was incubated for 1 h at 20–25 °C. After incubation, the development solution was added to each well on the plate and incubated for an additional hour. Finally, the reaction was stopped in each well, and after gentle mixing, fluorescence emission was recorded in a microplate reader. Kinase activity was quantified using a microplate reader capable of excitation at 400 nm and emission at 445 nm (coumarin) and 520 nm (fluorescein). Furthermore, the %phosphorylation (%P) was calculated using the following formula:

$$\text{\%P }=1-\frac{(\text{Emission ratio ∗ }F100\%)-C100\%}{\left(C0\%-C100\%)+[\text{Emission ratio ∗ }(F100\%-F0\%)\right]}$$

Where, Emission ratio = Coumarin emission (445 nm)/Fluorescein emission (520 nm); C0% = Average Coumarin emission signal of the 0% Phos. Control; C100% = Average Coumarin emission signal of the 100% Phos. Control: F0% = Average Fluorescein emission signal of the 0% Phos. Control: F100% = Average Fluorescein emission signal of the 100% Phos. Control.

#### Tubulin assay

The tubulin polymerisation assay was performed using the BK004P HTS kit (Cytoskeleton Inc.) for high-throughput screening. It is carried out to evaluate the effects of investigational compounds on microtubule dynamics using an optical density (OD)-based method. It utilises highly purified tubulin (>97%) isolated from porcine brain, which undergoes GTP-dependent polymerisation under appropriate buffer conditions and gets converted into microtubules. An increase in solution turbidity indicates the formation of microtubules, and polymerisation is recorded using a microplate reader maintained at 340 nm.

#### Reactive oxygen species (ROS) and mitochondrial membrane potential assay

The cellular alterations in response to the potent molecules JRC-2 and JRC-6, involving reactive oxygen species (ROS) and mitochondrial membrane potential (Δψm), were investigated using H_2_DCFDA and JC-1 assays, with paclitaxel serving as a positive control. MCF-7 cells were seeded in a precoated sterile 6-well plate, and investigational compounds were treated at their respective IC_50_ values for 24h. For ROS analysis, cells were washed with 1X PBS and incubated with 10 µM H_2_DCFDA in PBS for 30 min at 37 °C in the dark. Excess dye was washed twice with PBS, and fluorescence was measured using a microplate reader at excitation/emission wavelengths of 485 nm and 535 nm, respectively. Similarly, the JC-1 assay was performed using a 20 µM working concentration of JC-1 dye and incubated for 30 min at 37 °C in the dark. Excess dye was washed twice with 1X PBS, and after sample preparation, flow cytometry was performed to analyse the red-to-green fluorescence ratio, which indicates the extent of mitochondrial depolarisation.

### Computational studies

#### Protein preparation

The crystal structures of wild-type EGFR bound to erlotinib (PDB ID: 1M17) and tubulin bound to taxol (PDB ID: 1JFF) were retrieved from the Protein Data Bank^55^. Structural preparation was performed using the Protein Preparation Workflow in Maestro (Schrödinger)^56^. During pre-processing, hydrogen atoms were added, and potential metal coordination states were assigned. Missing residues, side chains, or loops (where applicable) were completed using the Prime module of Schrödinger’s software, and appropriate protonation and tautomeric states for residues such as Asp, Glu, Arg, Lys, and His were generated assuming a physiological pH of 7.0 ± 2.0. Hydrogen-bond optimisation was performed using PROPKA at pH 7.0^57^. Water molecules forming fewer than two hydrogen-bond interactions with the protein environment were discarded. Final structure refinement was performed by restrained energy minimisation using OPLS4 force field^58^ to correct steric clashes and relieve strain. The minimisation was terminated upon achieving convergence at an average protein-heavy-atom RMSD of 0.3 Å.

#### Ligand preparation

Investigational compounds **JRC-2**, **JRC-6**, and **5a**, together with the reference drugs erlotinib and paclitaxel, were processed using the LigPrep module^59^ within the Schrödinger software package^56^. Ionisation and tautomeric states appropriate for pH 7.0 ± 2.0 were generated using Epik for pKa estimation. The final ligand structures were then subjected to energy minimisation using the OPLS4 force field^58^.

#### Molecular docking

Molecular docking of investigational compounds **JRC-2**, **JRC-6**, and **5a**, along with the reference ligands erlotinib and paclitaxel, was performed using the Glide module^60^^,^^61^ in Maestro (Schrödinger Release 2024–2)^56^. The previously prepared EGFR–erlotinib (PDB ID: 1M17) and tubulin–taxol (PDB ID: 1JFF) structures served as the basis for generating individual receptor grids. For each target, a cubic docking grid (20 Å side length) was centred on the centroid of the co-crystallized ligand. No positional or interaction constraints were defined during grid setup. Docking experiments were executed under default Glide settings in standard precision (SP) mode, with full ligand flexibility enabled, including ring sampling and nitrogen inversion. The standard van der Waals scaling parameters were applied (0.8 for nonpolar ligand atoms with a 0.15 partial charge cutoff). Following docking, post-minimisation of poses was performed, and up to five poses per ligand were retained. The top-scoring pose for each compound was identified using the default Glide scoring function. All calculations used the OPLS4 force field. Docking protocol validation was performed by redocking the native crystallographic ligands into their respective receptor structures^60^^,^^61^. In both targets, the Glide SP protocol successfully reproduced the experimentally observed binding modes, showing only minor RMSD deviations from the original X-ray poses.

#### Molecular dynamics (MD) simulations

Classical molecular dynamics (MD) simulations were conducted in Desmond^62^ (Schrödinger Release 2024–2)^56^ using the Glide-derived binding poses of compounds **JRC-2** and **JRC-6** within tubulin as starting structures. Each protein–ligand system was embedded in an orthorhombic TIP3P water box^63^ with periodic boundary conditions, allowing a minimum 10 Å buffer between any solute atom and the box boundaries. Counterions (Na^+^/Cl^-^) were added to neutralise the system charge, and physiological ionic strength was reproduced by introducing 0.15 M NaCl. System preparation included minimisation and equilibration using the default Desmond workflow under NPT conditions^62^. All simulations employed the OPLS4 force field^58^. Production MD runs were carried out for 200 ns per system, with trajectory frames saved every 200 ps. Temperature and pressure were maintained at 300 K and 1.01325 bar, respectively, using a Nosé-Hoover thermostat combined with a Martyna-Tobias-Klein barostat with isotropic pressure control^64–66^. Trajectory analysis was performed using the Simulation Interaction Diagram (SID) tool in Schrödinger^56^, focusing primarily on root-mean-square deviation (RMSD) profiles of both the protein and the ligand throughout the simulation.

## Supplementary Material

Supplemantary information_carboxamide_080426.docx

## Data Availability

The authors confirm that the data supporting the findings of this study are available within the article and its supplementary materials. The docking and MD simulation files are available from the corresponding authors (RK, VJ) on request.

## Supplementary material

The Supplementary data include ^1^H and ^13^C NMR spectra, as well as HRMS spectra of the final compounds. It also includes the IR spectra of intermediate compounds **4** and **JR**. Cellular data demonstrating non-toxicity against HEK-293 cells and co-ordinates of optimised geometries of reactant, intermediates, and product are also included.

## References

[1] Bray F, Laversanne M, Sung H, Ferlay J, Siegel RL, Soerjomataram I, Jemal A. Global cancer statistics 2022: GLOBOCAN estimates of incidence and mortality worldwide for 36 cancers in 185 countries. CA Cancer J Clin. 2024;74(3):229–263. 38572751 10.3322/caac.21834

[2] Hanahan D. Hallmarks of cancer: new dimensions. Cancer Discov. 2022;12(1):31–46. 35022204 10.1158/2159-8290.CD-21-1059

[3] Dupont CA, Riegel K, Pompaiah M, Juhl H, Rajalingam K. Druggable genome and precision medicine in cancer: current challenges. Febs J. 2021;288(21):6142–6158. 33626231 10.1111/febs.15788

[4] Blair HA. Befotertinib: first approval. Drugs. 2023;83(15):1433–1437. 37751131 10.1007/s40265-023-01946-w

[5] Gao C, Wang W, Liu T, Li X, Yu Y, Wu J. Annual review of EGFR inhibitors in 2024. Eur J Med Chem. 2025;292:117677. 40328037 10.1016/j.ejmech.2025.117677

[6] Dhillon S. Sunvozertinib: first approval. Drugs. 2023;83(17):1629–1634. 37962831 10.1007/s40265-023-01959-5

[7] Ding J, Ding X, Zeng J, Liu X. Furmonertinib for EGFR-mutant advanced non-small cell lung cancer: a glittering diamond in the rough of EGFR-TKI. Front Pharmacol. 2024;15:1357913. 38440180 10.3389/fphar.2024.1357913 PMC10910349

[8] Corvaja C, Passaro A, Attili I, Aliaga PT, Spitaleri G, Signore ED, de Marinis F. Advancements in fourth-generation EGFR TKIs in EGFR-mutant NSCLC: bridging biological insights and therapeutic development. Cancer Treat Rev. 2024;130:102824. 39366135 10.1016/j.ctrv.2024.102824

[9] Kumar M, Joshi G, Chatterjee J, Kumar R. Epidermal growth factor receptor and its trafficking regulation by acetylation: implication in resistance and exploring the newer therapeutic avenues in cancer. Curr Top Med Chem. 2020;20(12):1105–1123. 32031073 10.2174/1568026620666200207100227

[10] Normanno N, De Luca A, Bianco C, Strizzi L, Mancino M, Maiello MR, Carotenuto A, De Feo G, Caponigro F, Salomon DS, et al. Epidermal growth factor receptor (EGFR) signaling in cancer. Gene. 2006;366(1):2–16. 16377102 10.1016/j.gene.2005.10.018

[11] Jordan MA, Wilson L. Microtubules as a target for anticancer drugs. Nat Rev Cancer. 2004;4(4):253–265. 15057285 10.1038/nrc1317

[12] Brouhard GJ, Rice LM. The contribution of αβ-tubulin curvature to microtubule dynamics. J Cell Biol. 2014;207(3):323–334. 25385183 10.1083/jcb.201407095 PMC4226729

[13] Horio T, Murata T, Murata T. The role of dynamic instability in microtubule organization. Front Plant Sci. 2014;5:511. 25339962 10.3389/fpls.2014.00511 PMC4188131

[14] Shuai W, Wang G, Zhang Y, Bu F, Zhang S, Miller DD, Li W, Ouyang L, Wang Y. Recent progress on tubulin inhibitors with dual targeting capabilities for cancer therapy. J Med Chem. 2021;64(12):7963–7990. 34101463 10.1021/acs.jmedchem.1c00100

[15] Stanton RA, Gernert KM, Nettles JH, Aneja R. Drugs that target dynamic microtubules: a new molecular perspective. Med Res Rev. 2011;31(3):443–481. 21381049 10.1002/med.20242 PMC3155728

[16] Reszka AA, Bulinski JC, Krebs EG, Fischer EH. Mitogen-activated protein kinase/extracellular signal-regulated kinase 2 regulates cytoskeletal organization and chemotaxis via catalytic and microtubule-specific interactions. Mol Biol Cell. 1997;8(7):1219–1232. 9243503 10.1091/mbc.8.7.1219 PMC276148

[17] Reszka AA, Seger R, Diltz CD, Krebs EG, Fischer EH. Association of mitogen-activated protein kinase with the microtubule cytoskeleton. Proc Natl Acad Sci USA. 1995;92(19):8881–8885. 7568036 10.1073/pnas.92.19.8881 PMC41071

[18] Gundersen GG, Cook TA. Microtubules and signal transduction. Curr Opin Cell Biol. 1999;11(1):81–94. 10047525 10.1016/s0955-0674(99)80010-6

[19] Etienne-Manneville S. From signaling pathways to microtubule dynamics: the key players. Curr Opin Cell Biol. 2010;22(1):104–111. 20031384 10.1016/j.ceb.2009.11.008

[20] Podolak M, Holota S, Deyak Y, Dziduch K, Dudchak R, Wujec M, Bielawski K, Lesyk R, Bielawska A. Tubulin inhibitors. Selected scaffolds and main trends in the design of novel anticancer and antiparasitic agents. Bioorg Chem. 2024;143:107076. 38163424 10.1016/j.bioorg.2023.107076

[21] Wang X, Wang Y, Wang X, Wang Y, Liu Q. Discovery of the erianin derivatives as EGFR/tubulin dual-target inhibitors that suppress the proliferation and invasion of non-small cell lung cancer through autophagy-dependent ferroptosis. Bioorg Chem. 2025;166:109110. 41110239 10.1016/j.bioorg.2025.109110

[22] Liu T, Wan Y, Xiao Y, Xia C, Duan G. Dual-target inhibitors based on HDACs: novel antitumor agents for cancer therapy. J Med Chem. 2020;63(17):8977–9002. 32320239 10.1021/acs.jmedchem.0c00491

[23] Chen J-F, Guo S-J, He B, Zheng W, Jiang W-J, Yuan Z, Xiang Y, Peng C, Xiong W, Shi J-Y, et al. Advances of dual inhibitors based on ALK for the treatment of cancer. Bioorg Chem. 2025;159:108417. 40168884 10.1016/j.bioorg.2025.108417

[24] Seo YH. Dual inhibitors against topoisomerases and histone deacetylases. J Cancer Prev. 2015;20(2):85–91. 26151040 10.15430/JCP.2015.20.2.85 PMC4492363

[25] Pang X, Xu W, Liang J, Liu Y, Li H, Chen L. Research progress and perspectives of dual-target inhibitors. Eur J Med Chem. 2025;289:117453. 40024166 10.1016/j.ejmech.2025.117453

[26] Joshi G, Yadav UP, Rafiq Z, Grewal P, Kumar M, Singh T, Jha V, Sharma P, Eriksson LA, Srinivas L, et al. Design and synthesis of topoisomerases-histone deacetylase dual targeted quinoline-bridged hydroxamates as anticancer agents. J Med Chem. 2025;68(3):2849–2868. 39808731 10.1021/acs.jmedchem.4c02135

[27] Bhat ZR, Kumar M, Sharma N, Yadav UP, Singh T, Joshi G, Pujala B, Raja M, Chatterjee J, Tikoo K, et al. In vivo anticancer evaluation of 6b, a non-covalent imidazo[1,2-a]quinoxaline-based epidermal growth factor receptor inhibitor against human xenograft tumor in nude mice. Molecules. 2022;27(17):5540. 10.3390/molecules27175540 PMC9457798 36080307

[28] Kumar M, Joshi G, Arora S, Singh T, Biswas S, Sharma N, Bhat ZR, Tikoo K, Singh S, Kumar R, et al. Design and synthesis of non-covalent imidazo[1,2-a]quinoxaline-based inhibitors of EGFR and their anticancer assessment. Molecules. 2021;26(5):1490. 10.3390/molecules26051490 PMC7967119 33803355

[29] Kumar M, Patil KT, Maity P, Chatterjee J, Singh T, Joshi G, Singh S, Kumar R. Design, synthesis, and anticancer assessment of structural analogues of (E)-1-((3,4,5-trimethoxybenzylidene)amino)-4-(3,4,5-trimethoxyphenyl)imidazo[1,2-a]quinoxaline-2-carbonitrile (6b), an imidazo[1,2-a]quinoxaline-based non-covalent EGFR inhibitor. RSC Med Chem. 2024;15(7):2322–2339. 39026650 10.1039/d4md00237g PMC11253857

[30] Kumar P, Prasad Yadav U, Joshi G, Arora S, Kumar M, Chatterjee J, et al. Imidazo[1,2-a]quinoxaline-2-carbonitrile derivative (RA-22) inhibits self-renewal and growth of cancer stem and cancer cells via downregulating AKT pathway. ChemistrySelect. 2024;9:e202400223.

[31] Joshi G, Chauhan M, Kumar R, Thakur A, Sharma S, Singh R, et al. Cyclocondensation reactions of an electron deactivated 2-aminophenyl tethered imidazole with mono/1,2-biselectrophiles: synthesis and DFT studies on the rationalization of imidazo[1,2-a]quinoxaline versus benzo[f]imidazo[1,5-a][1,3,5]triazepine selectivity switches. Org Chem Front. 2018;5:3526–3533.

[32] Courbet A, Bec N, Constant C, Larroque C, Pugniere M, El Messaoudi S, Zghaib Z, Khier S, Deleuze-Masquefa C, Gattacceca F, et al. Imidazoquinoxaline anticancer derivatives and imiquimod interact with tubulin: characterization of molecular microtubule inhibiting mechanisms in correlation with cytotoxicity. PLoS One. 2017;12(8):e0182022. 28797090 10.1371/journal.pone.0182022 PMC5552358

[33] Ward MA, Abdallah AE, Zayed MF, Ayyad RR, El-Zahabi MA. Design, synthesis and biological evaluation of newly triazolo-quinoxaline based potential immunomodulatory anticancer molecules. J Mol Struct. 2024;1298:137041.

[34] Ayoup MS, Rabee AR, Abdel-Hamid H, Amer A, Abu-Serie MM, Ashraf S, Ghareeb DA, Ibrahim RS, Hawsawi MB, Negm A, et al. Design and synthesis of quinoxaline hybrids as modulators of HIF-1a, VEGF, and p21 for halting colorectal cancer. ACS Omega. 2024;9(23):24643–24653. 38882127 10.1021/acsomega.4c01075 PMC11170630

[35] Liang JH, Cho ST, Shih TL, Chen JJ. Synthesis of quinoxalines and assessment of their inhibitory effects against human non-small-cell lung cancer cells. RSC Adv. 2024;14(39):28659–28668. 39252995 10.1039/d4ra04453c PMC11382098

[36] Ismail MMF, Shawer TZ, Ibrahim RS, Abusaif MS, Kamal MM, Allam RM, Ammar YA. Novel quinoxaline-3-propanamides as VGFR-2 inhibitors and apoptosis inducers. RSC Adv. 2023;13(45):31908–31924. 37915441 10.1039/d3ra05066a PMC10616755

[37] Ismail MA, Abusaif MS, El-Gaby MSA, Ammar YA, Ragab A. A new class of antiproliferative activity and apoptotic inducer with molecular docking studies for a novel of 1,3-dithiolo[4,5-b]quinoxaline derivatives hybrid with a sulfonamide moiety. RSC Adv. 2023;13(18):12589–12608. 37101951 10.1039/d3ra01635h PMC10123497

[38] Salem MG, Abu El-Ata SA, Elsayed EH, Mali SN, Alshwyeh HA, Almaimani G, Almaimani RA, Almasmoum HA, Altwaijry N, Al-Olayan E, et al. Novel 2-substituted-quinoxaline analogs with potential antiproliferative activity against breast cancer: insights into cell cycle arrest, topoisomerase II, and EGFR activity. RSC Adv. 2023;13(47):33080–33095. 37954422 10.1039/d3ra06189b PMC10633821

[39] Lanigan RM, Sheppard TD. Recent developments in amide synthesis: direct amidation of carboxylic acids and transamidation reactions. European J Org Chem. 2013;2013:7453–7465.

[40] Meanwell NA. Synopsis of some recent tactical application of bioisosteres in drug design. J Med Chem. 2011;54(8):2529–2591. 21413808 10.1021/jm1013693

[41] Tam CS, Muñoz JL, Seymour JF, Opat S. Correction: zanubrutinib: past, present, and future. Blood Cancer J. 2023;13(1):141. 37696810 10.1038/s41408-023-00902-x PMC10495438

[42] Nguyen TB, Ermolenko L, Al-Mourabit A. Sodium sulfide: a sustainable solution for unbalanced redox condensation reaction between o-nitroanilines and alcohols catalyzed by an iron–sulfur system. Synthesis (Stuttg). 2015;47:1741–1748.

[43] Das K, Mondal A, Srimani D. Phosphine free Mn-complex catalyzed dehydrogenative C–C and C–heteroatom bond formation: a sustainable approach to synthesize quinoxaline, pyrazine, benzothiazole and quinoline derivatives. Chem Commun (Camb). 2018;54(75):10582–10585. 30167623 10.1039/c8cc05877f

[44] Mohammadi H, Hamid Shaterian R, Shaterian HR. 3-Oxo-[1,2,4]triazolidin-1-yl)bis (butane-1-sulfonic acid) functionalized magnetic γ-Fe2O3 nanoparticles: A novel and heterogeneous nanocatalyst for one-pot and efficient four-component synthesis of novel spiro[indeno[1,2-b]quinoxaline derivatives. Appl Organomet Chem. 2019;33:e4901.

[45] Jafarpour M, Rezapour E, Ghahramaninezhad M, Rezaeifard A. A novel protocol for selective synthesis of monoclinic zirconia nanoparticles as a heterogeneous catalyst for condensation of 1,2-diamines with 1,2-dicarbonyl compounds. New J Chem. 2014;38:676–682.

[46] Sharma A, Dixit R, Sharma S, Dutta S, Yadav S, Arora B, et al. Efficient and sustainable Co3O4 nanocages based nickel catalyst: a suitable platform for the synthesis of quinoxaline derivatives. Mol Catal. 2021;504:111454.

[47] Das A, Thomas KRJ. Light promoted synthesis of quinoxalines and imidazo[1,2-a]pyridines via oxybromination from alkynes and alkenes. Asian J Org Chem. 2020;9:1820–1825.

[48] Hazarika D, Phukan P. Metal free synthesis of quinoxalines from alkynes via a cascade process using TsNBr2. Tetrahedron. 2017;73:1374–1379.

[49] Motakatla VKR, Gokanapalli A, Peddiahgari VGR. Cu–N-heterocyclic carbene-catalyzed synthesis of 2-aryl-3-(arylethynyl)quinoxalines from one-pot tandem coupling of o-phenylenediamines and terminal alkynes. Appl Organomet Chem. 2019;33:e5188.

[50] Wang LX, Hu BQ, Xiang JF, Cui J, Hao X, Liang TL, et al. Naryl-substituted anthranilamides with intramolecular hydrogen bonds. Tetrahedron. 2014;70:8588–8591.

[51] Mphahlele MJ, Maluleka MM, Rhyman L, Ramasami P, Mampa RM, Mphahlele MJ, et al. Spectroscopic, DFT, and XRD studies of hydrogen bonds in n-unsubstituted 2-aminobenzamides. Molecules. 2017;22(1):83. 10.3390/molecules22010083 PMC6155760 28054998

[52] Dey R, Banerjee T, Langer V, Ray S, Roychowdhury P. 5-amino-1-[2-(diethylamino)ethyl]-1 H-imidazole-4-carboxamide. Acta Crystallogr Sect E Struct Rep Online. 2006;62:o814–o816.

[53] Villalpando-Rodriguez GE, Gibson SB. Reactive oxygen species (ROS) regulates different types of cell death by acting as a rheostat. Oxid Med Cell Longev. 2021;2021:9912436. 34426760 10.1155/2021/9912436 PMC8380163

[54] Oparka M, Walczak J, Malinska D, van Oppen LMPE, Szczepanowska J, Koopman WJH, Wieckowski MR. Quantifying ROS levels using CM-H2DCFDA and HyPer. Methods. 2016;109:3–11. 27302663 10.1016/j.ymeth.2016.06.008

[55] Berman HM, Westbrook J, Feng Z, Gilliland G, Bhat TN, Weissig H, Shindyalov IN, Bourne PE. The Protein Data Bank. Nucleic Acids Res. 2000;28(1):235–242. 10592235 10.1093/nar/28.1.235 PMC102472

[56] Schrödinger Release Notes – Release 2024-2 [Internet]. [cited 2025 Dec 13]. Available from: https://www.schrodinger.com/life-science/download/release-notes/release-2024-2/.

[57] Olsson MHM, SØndergaard CR, Rostkowski M, Jensen JH. PROPKA3: consistent treatment of internal and surface residues in empirical pKa predictions. J Chem Theory Comput. 2011;7(2):525–537. 26596171 10.1021/ct100578z

[58] Lu C, Wu C, Ghoreishi D, Chen W, Wang L, Damm W, Ross GA, Dahlgren MK, Russell E, Von Bargen CD, et al. OPLS4: improving force field accuracy on challenging regimes of chemical space. J Chem Theory Comput. 2021;17(7):4291–4300. 34096718 10.1021/acs.jctc.1c00302

[59] Schrödinger Release Notes – Release 2024-1 [Internet]. [cited 2025 Dec 13]. Available from: https://www.schrodinger.com/life-science/download/release-notes/release-2024-1/.

[60] Halgren TA, Murphy RB, Friesner RA, Beard HS, Frye LL, Pollard WT, Banks JL. Glide: a new approach for rapid, accurate docking and scoring. 2. Enrichment factors in database screening. J Med Chem. 2004;47(7):1750–1759. 15027866 10.1021/jm030644s

[61] Friesner RA, Murphy RB, Repasky MP, Frye LL, Greenwood JR, Halgren TA, Sanschagrin PC, Mainz DT. Extra precision glide: docking and scoring incorporating a model of hydrophobic enclosure for protein − ligand complexes. J Med Chem. 2006;49(21):6177–6196. 17034125 10.1021/jm051256o

[62] Bowers KJ, Chow E, Xu H, Dror RO, Eastwood MP, Gregersen BA, et al. Scalable algorithms for molecular dynamics simulations on commodity clusters. Proceedings of the 2006 ACM/IEEE Conference on Supercomputing, SC’06. 2006.

[63] Jorgensen WL, Chandrasekhar J, Madura JD, Impey RW, Klein ML. Comparison of simple potential functions for simulating liquid water. J Chem Phys. 1983;79:926–935.

[64] Nosé S. A unified formulation of the constant temperature molecular dynamics methods. J Chem Phys. 1984;81:511–519.

[65] Wentzcovitch RM. Invariant molecular-dynamics approach to structural phase transitions. Phys Rev B Condens Matter. 1991;44(5):2358–2361. 9999791 10.1103/physrevb.44.2358

[66] Martyna GJ, Klein ML, Tuckerman M. Nosé–Hoover chains: the canonical ensemble via continuous dynamics. J Chem Phys. 1992;97:2635–2643.

