# Supplementary material for: Design and Pictet–Spengler enabled synthesis of carboxamide-substituted imidazo[1,2-a]quinoxalines as dual EGFR and tubulin targeting anticancer agents
Source: J Enzyme Inhib Med Chem. 2026 May 25;41(1):2673744. doi: 10.1080/14756366.2026.2673744 (PMC13202676; doi:10.1080/14756366.2026.2673744)
Supplement: Supplemantary information_carboxamide_080426.docx [file IENZ_A_2673744_SM0232.docx]

**Design and Pictet–Spengler Enabled Synthesis of Carboxamide-Substituted Imidazo[1,2-a]quinoxalines as Dual EGFR and Tubulin Targeting Anticancer Agents**

**Table of Contents**

1. Spectral data……………………………………………………………………………………………………………………………………...S3

2. Biological study……………………………………………………………………………………………………………...………………….S63

3. Co-ordinates of optimized geometries of reactant, intermediates, and product………….…………………………...………………………...S74

^1^H NMR of **JR**:


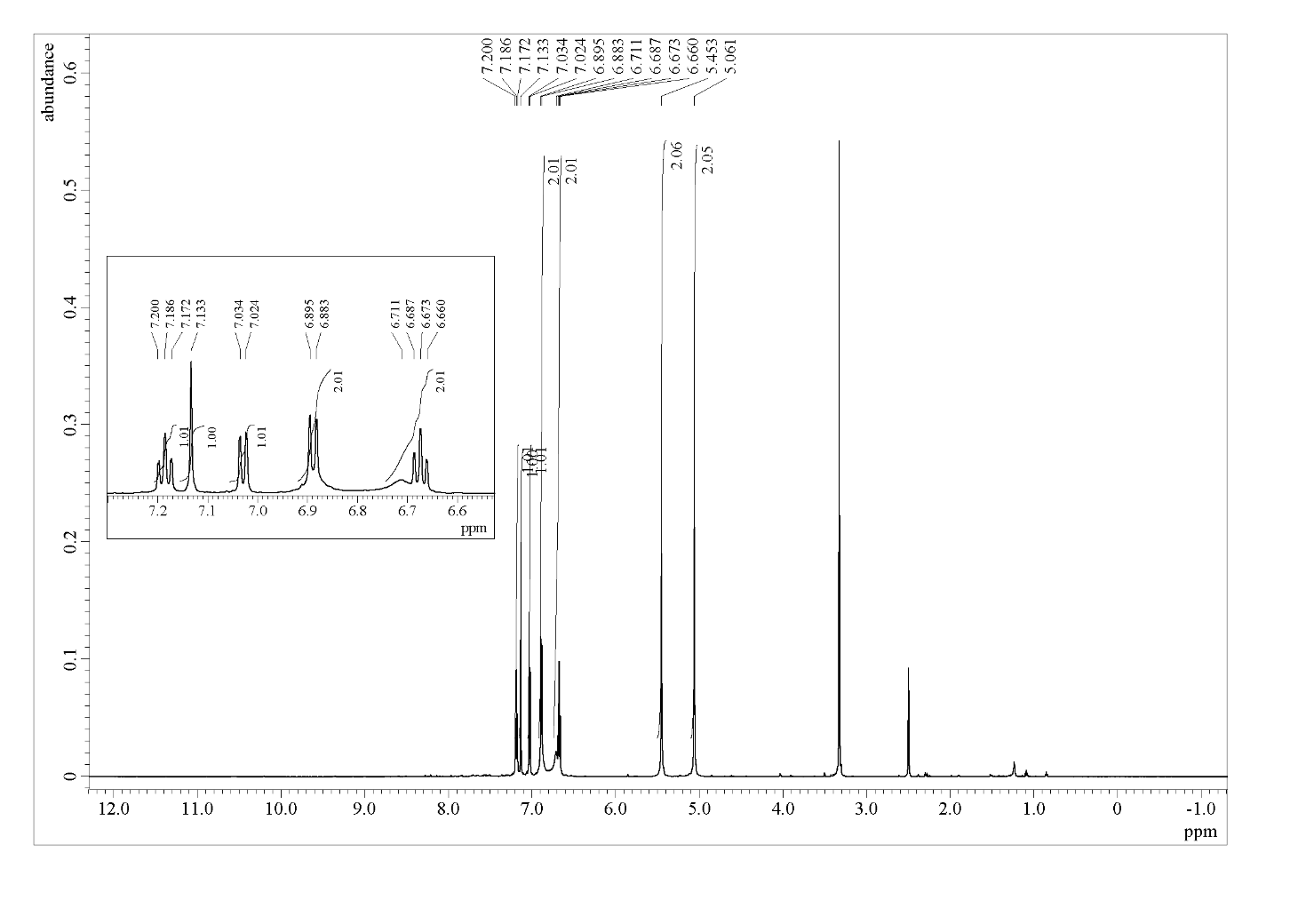


^13^C NMR of **JR**:


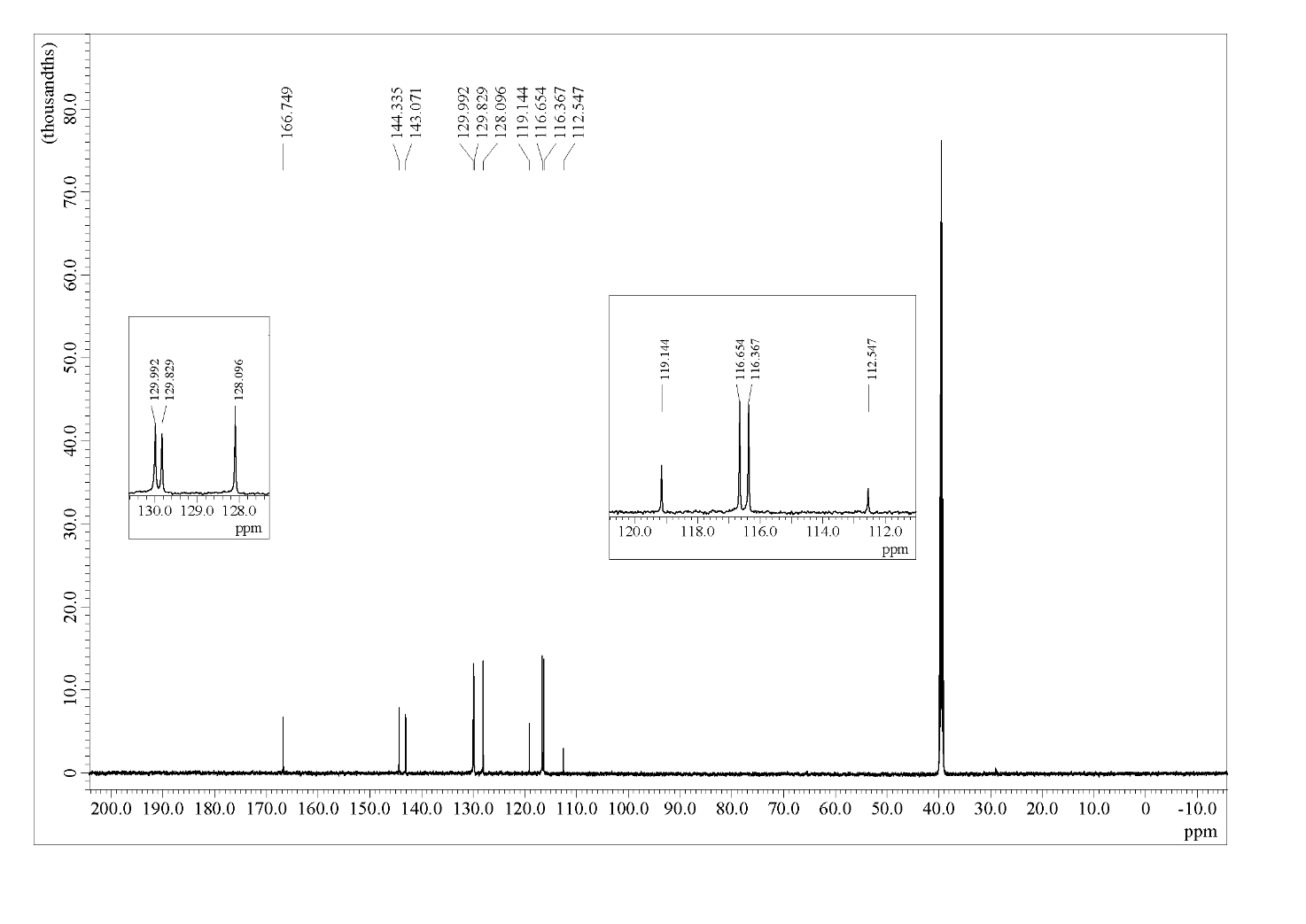


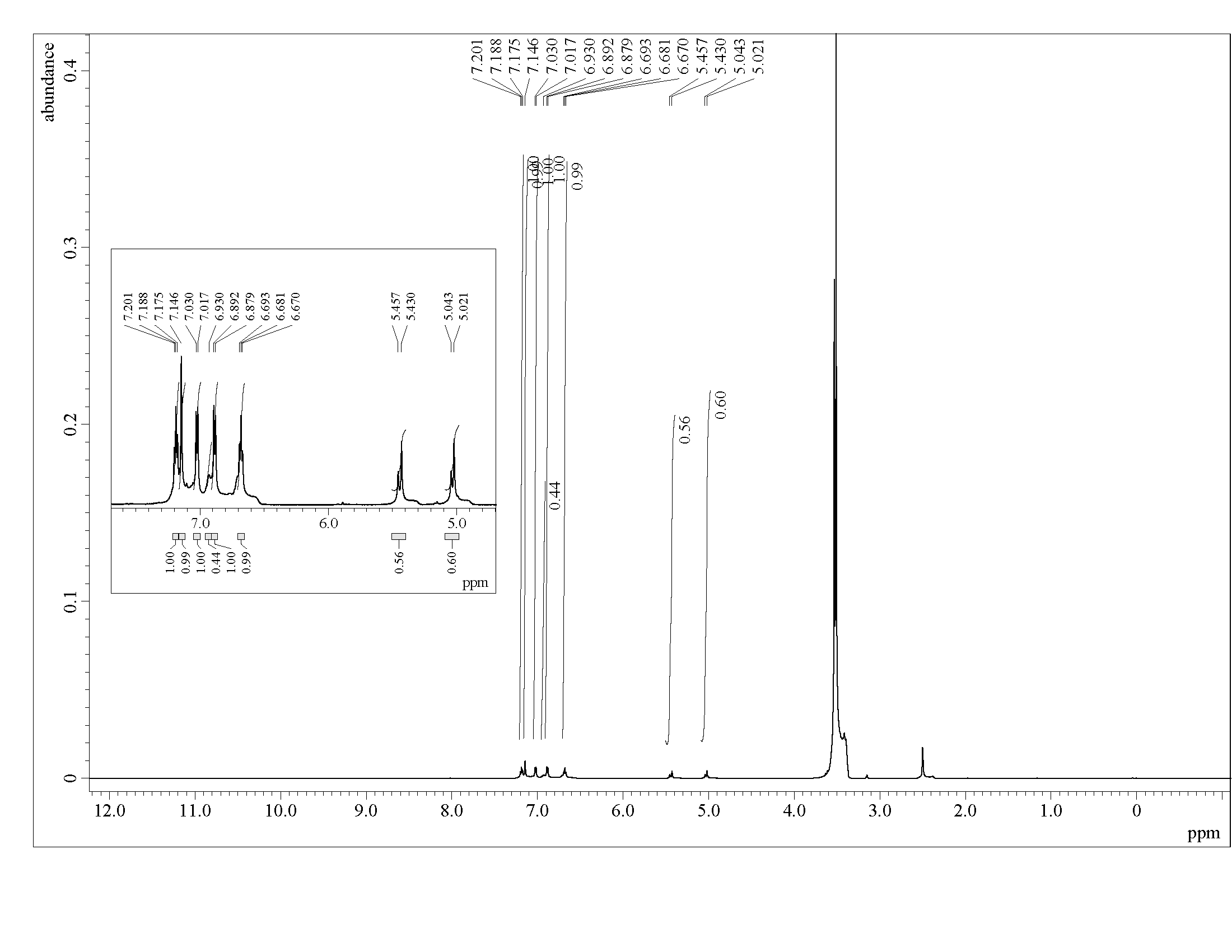


D_2_O exchange

Mass spectra of JR (EI):


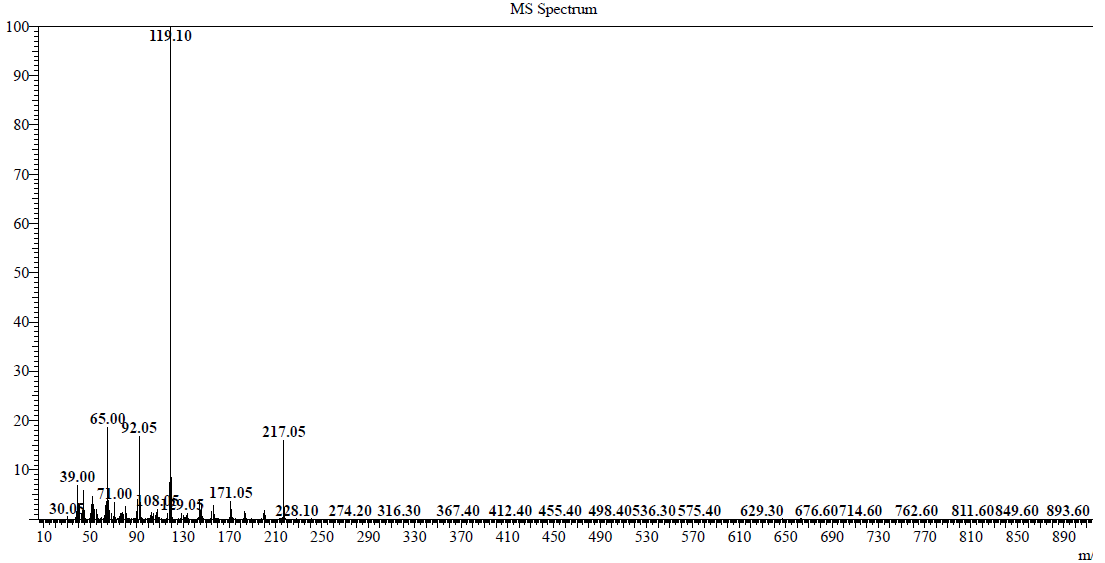


IR spectra of intermediate compound **4:**


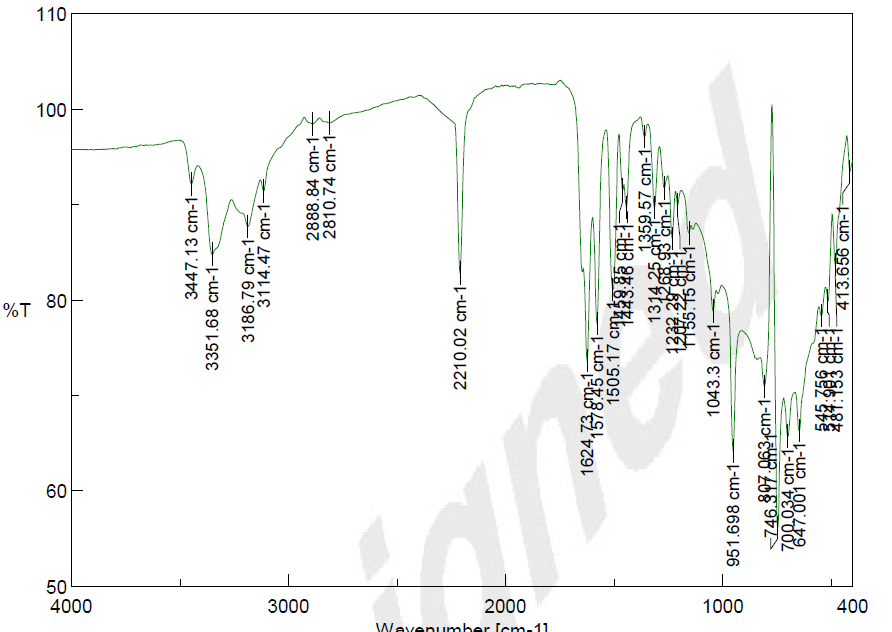


IR spectra of key intermediate **JR:**


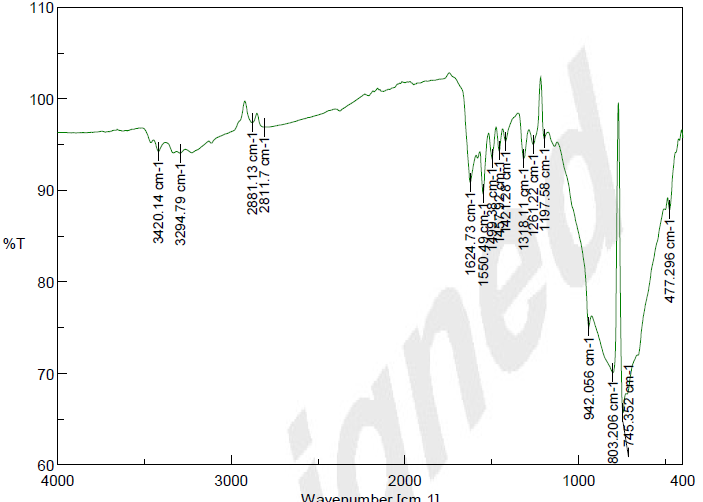


^1^H NMR of **JRC-1:**


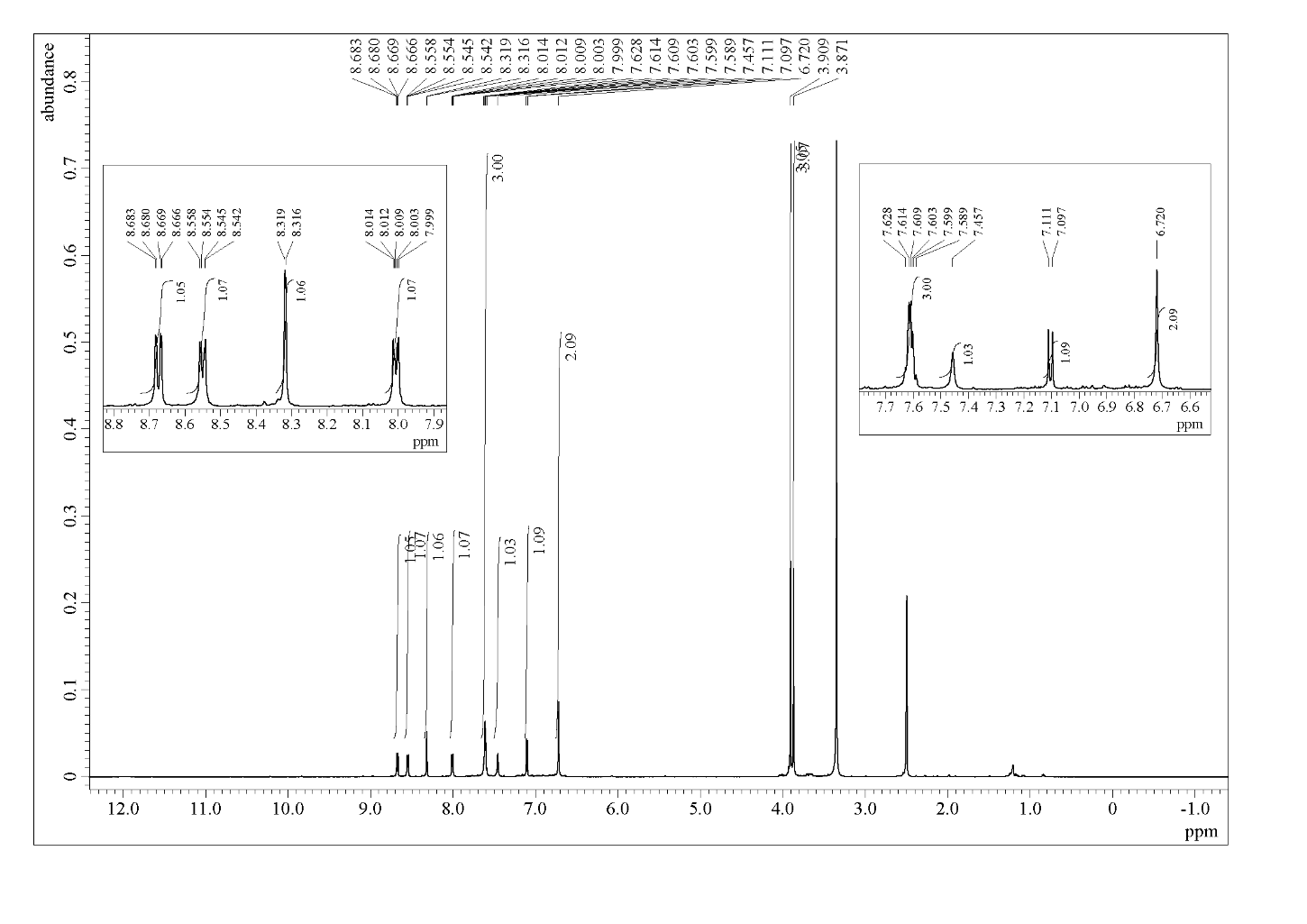


^13^C NMR of **JRC-1:**


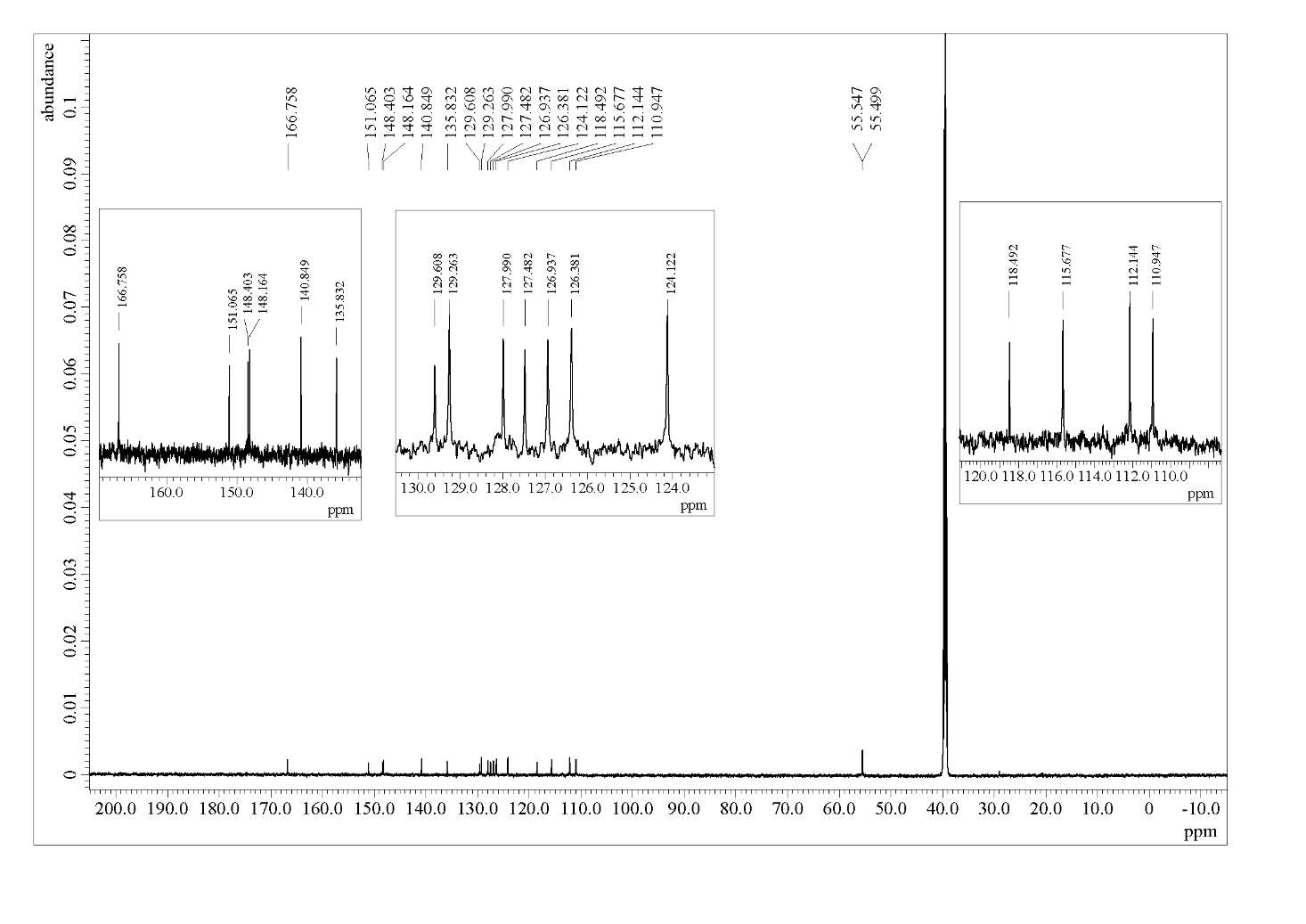


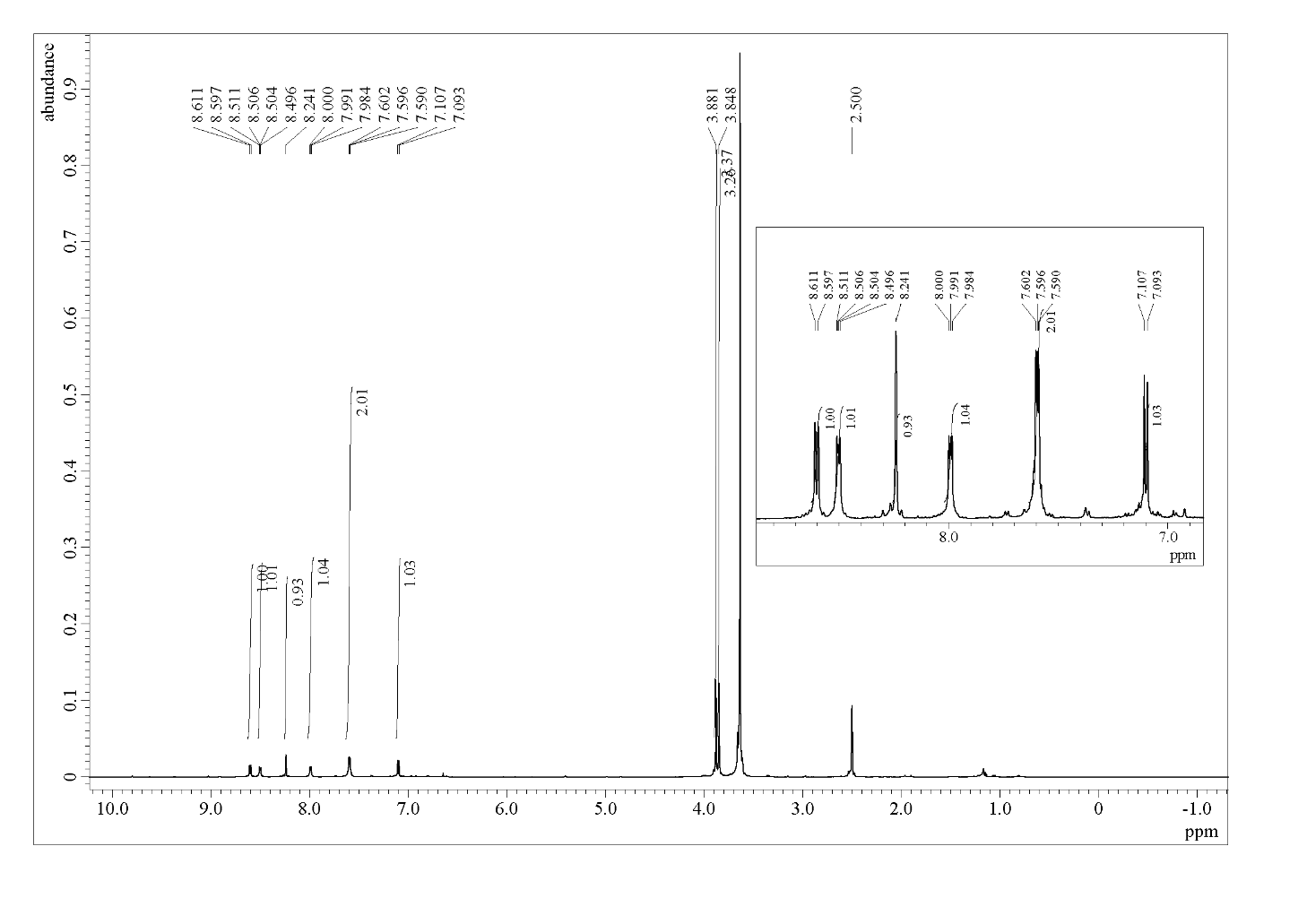


D_2_O exchange

HRMS of **JRC-1:**


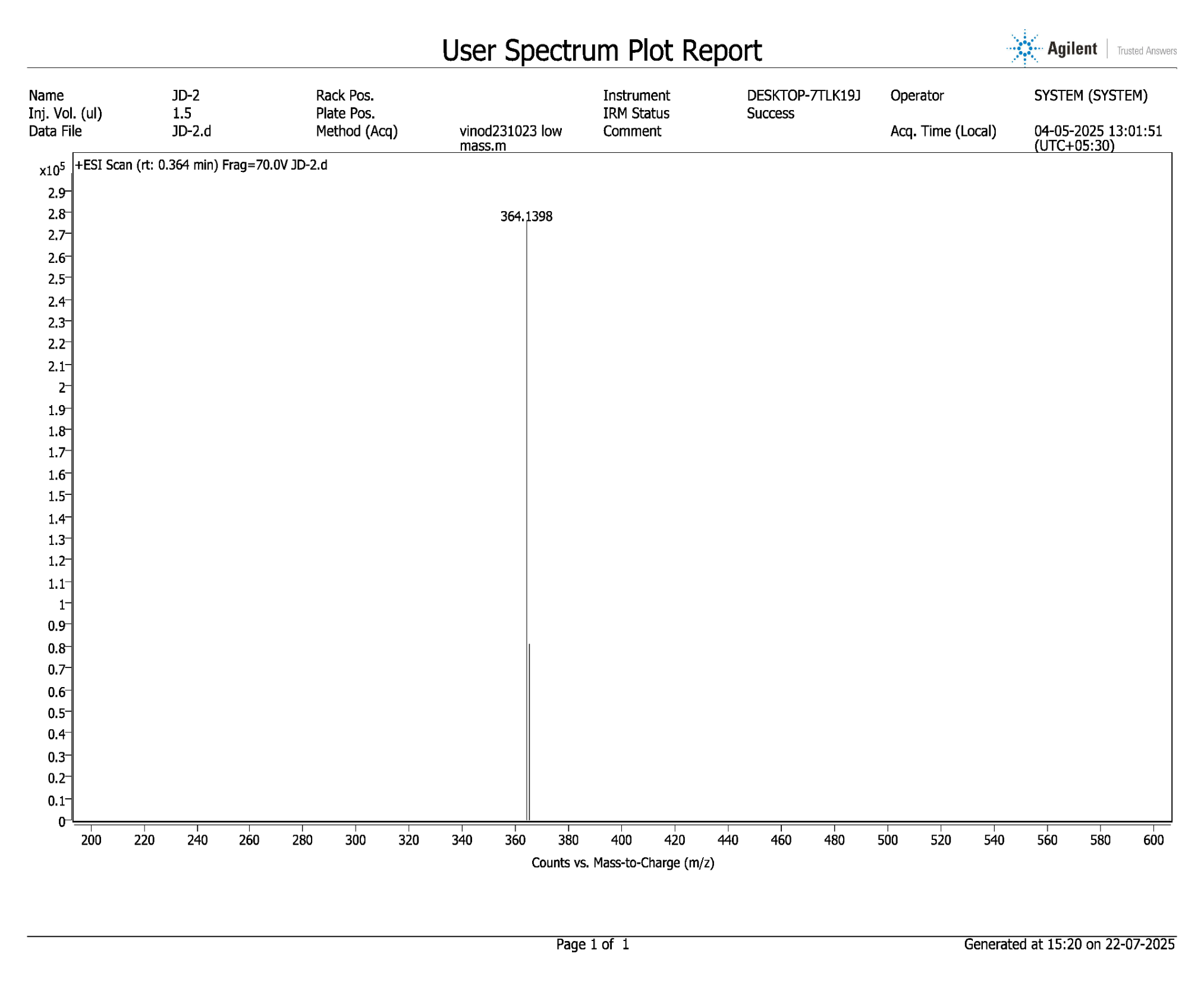


[M+H]^+^

^1^H NMR of **JRC-2**:


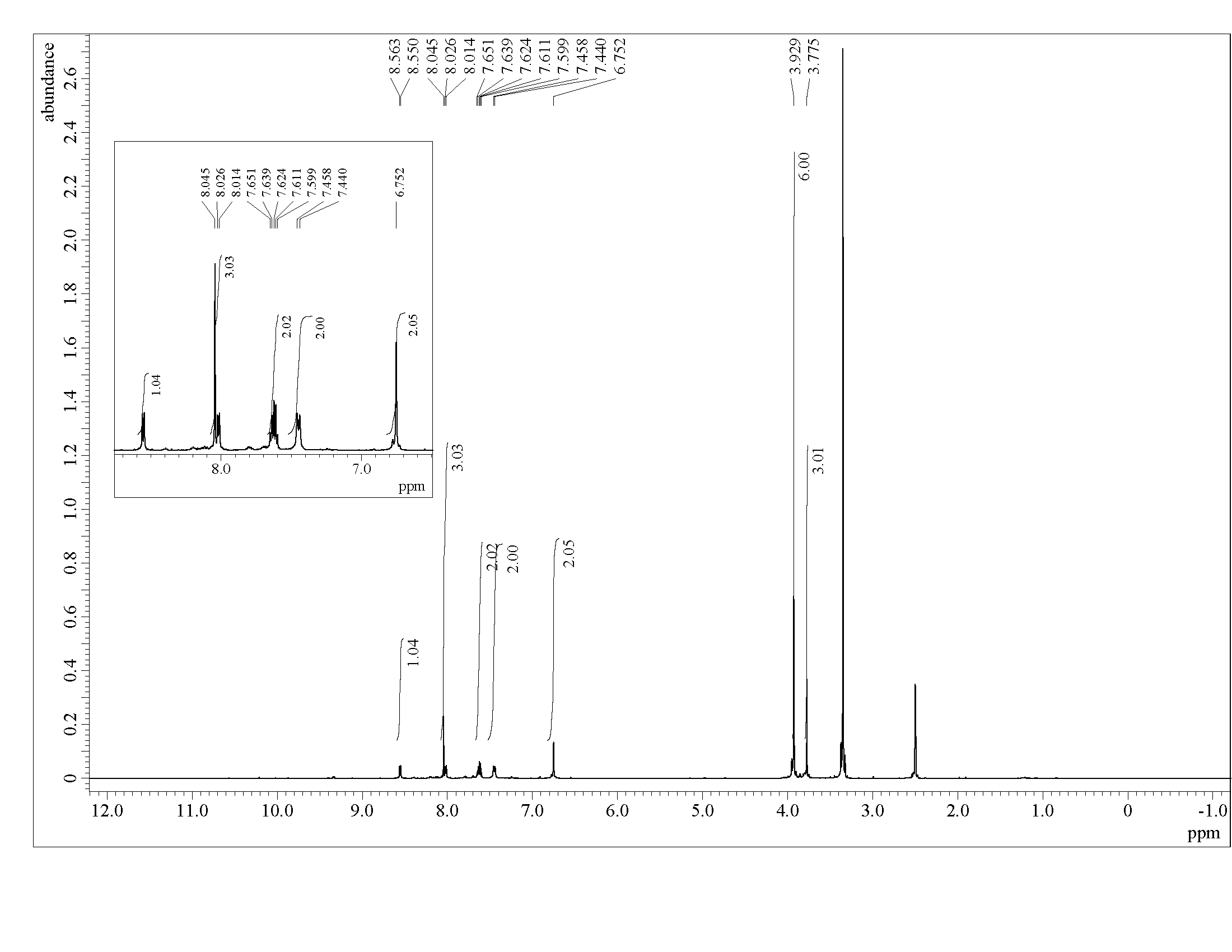


^13^C NMR of **JRC-2**:


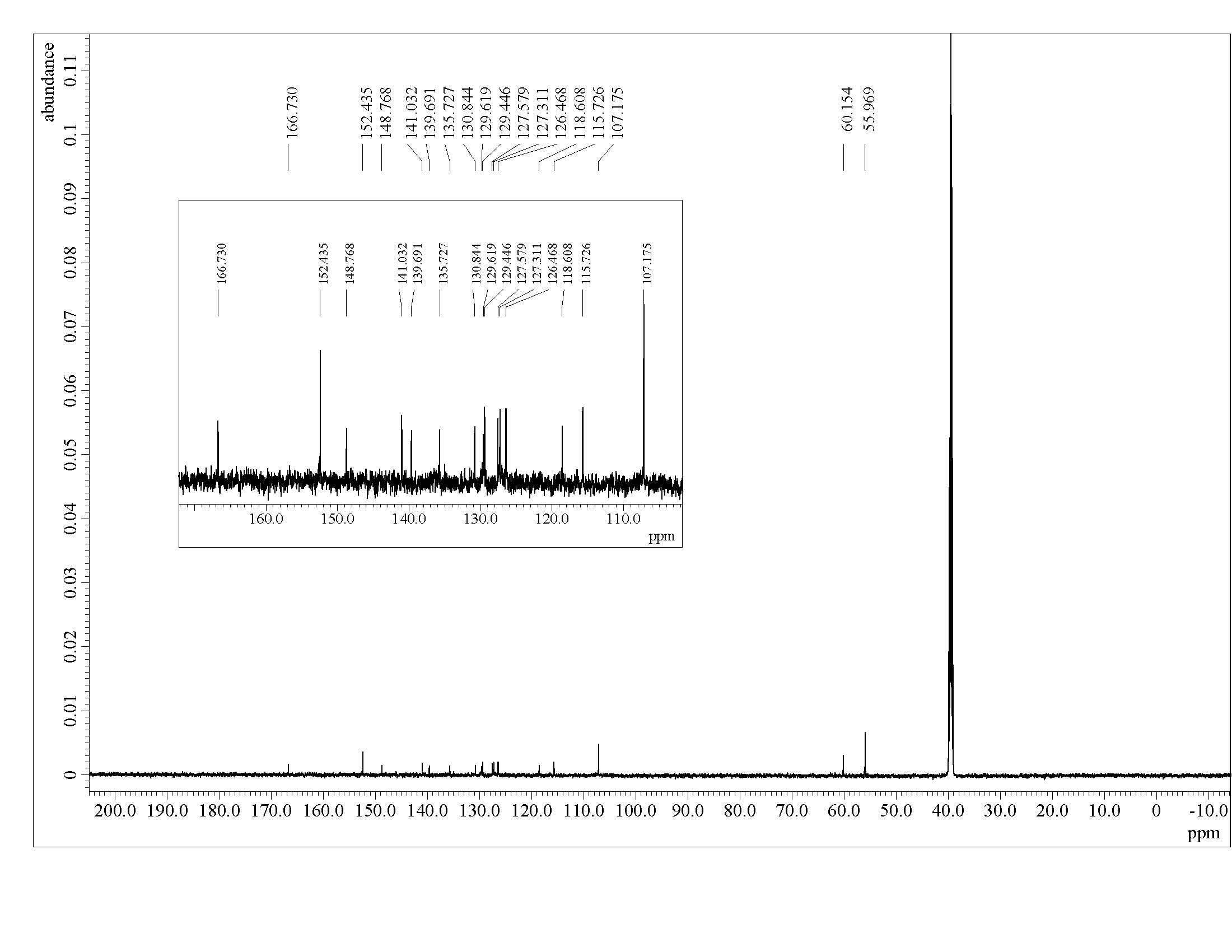


HRMS of **JRC-2**:


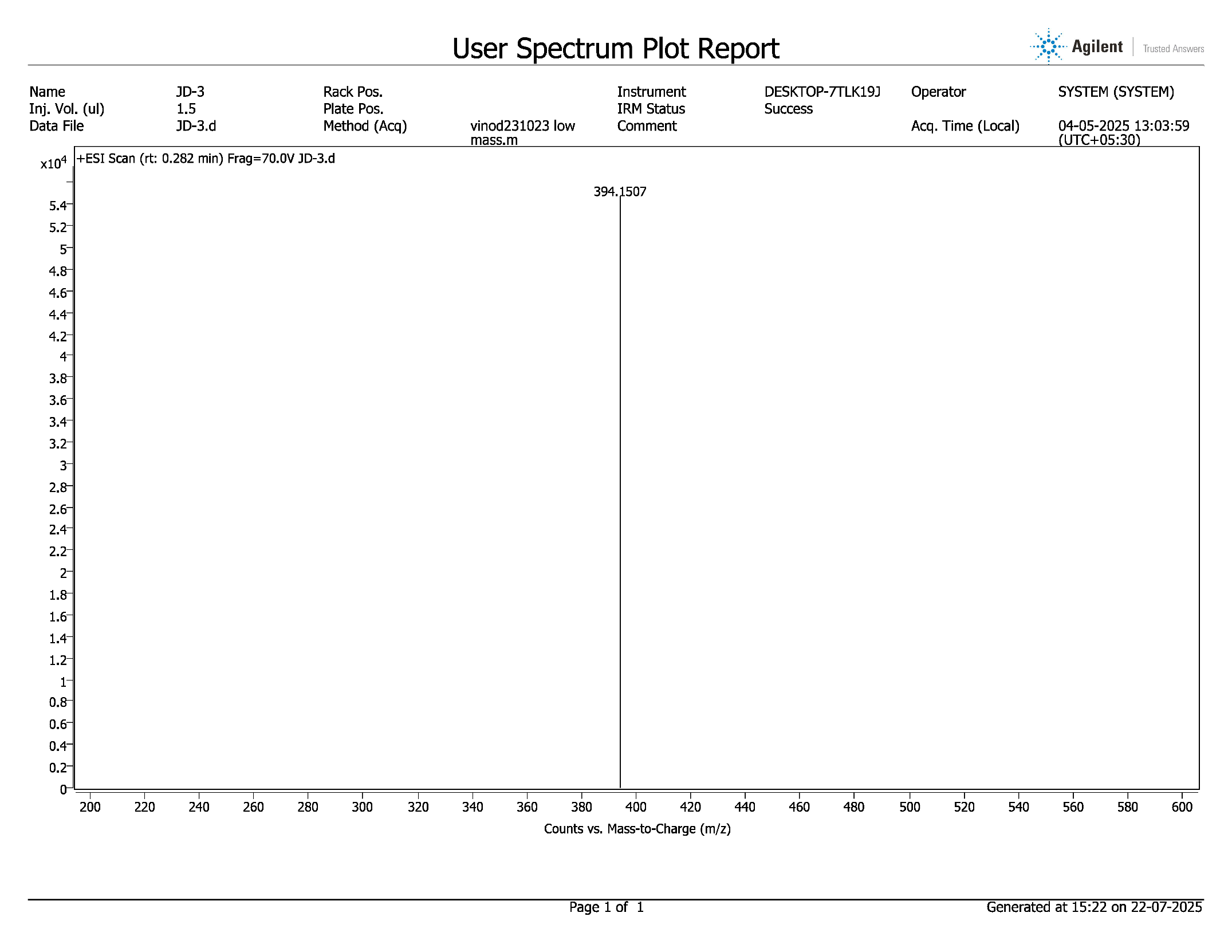


[M+H]^+^

^1^H NMR of **JRC-3**:


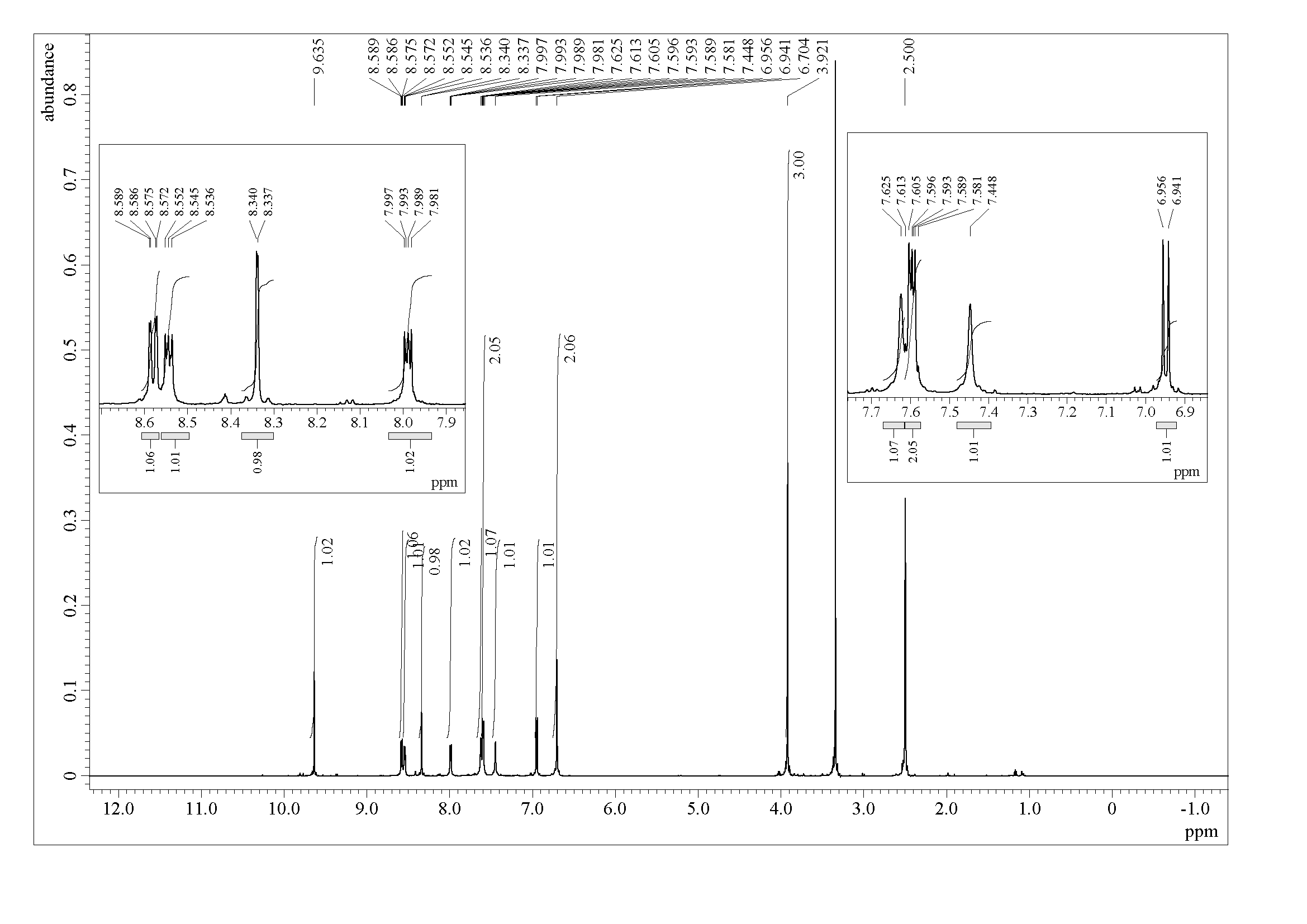


^13^C NMR of **JRC-3**:


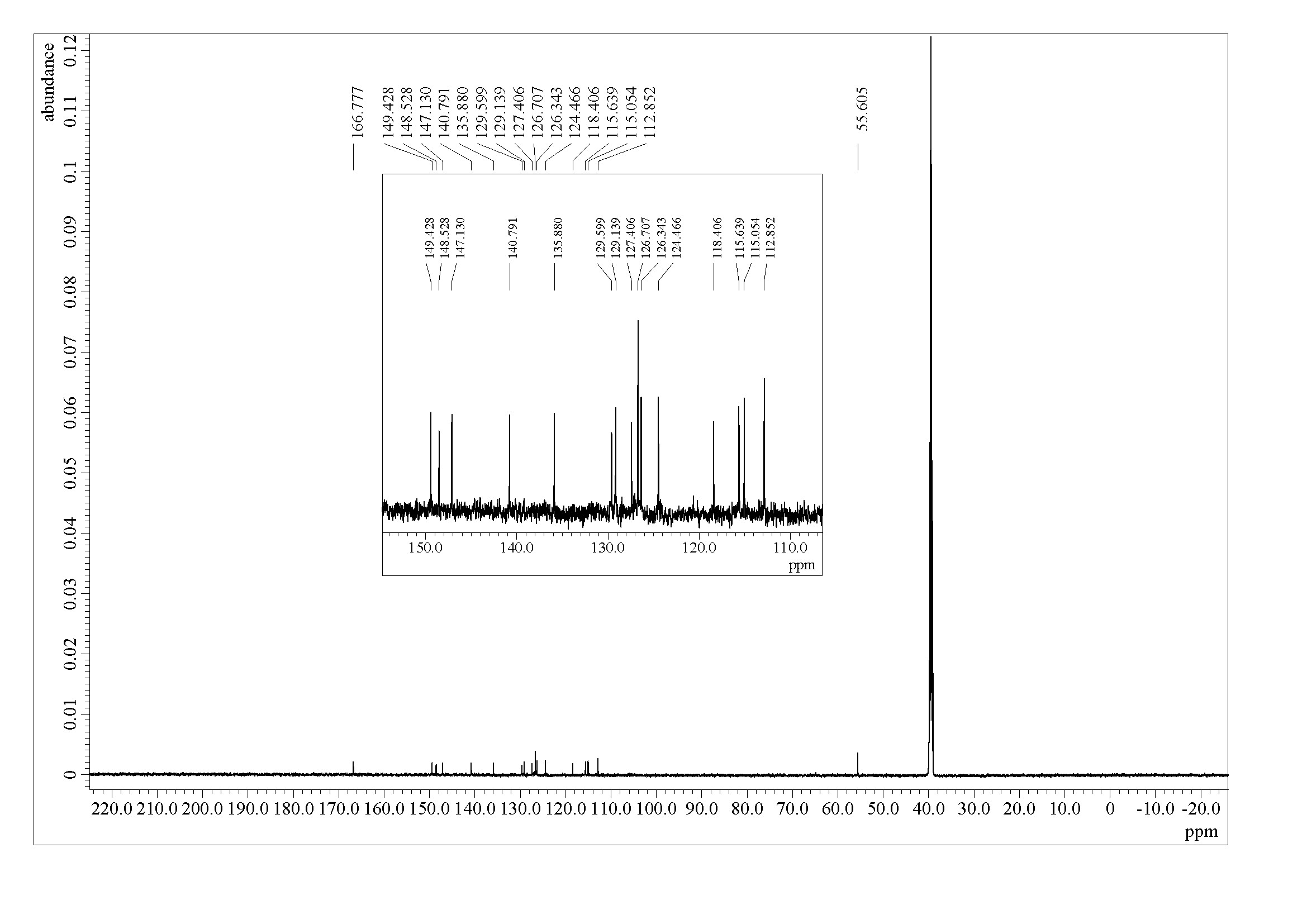


HRMS of **JRC-3**:


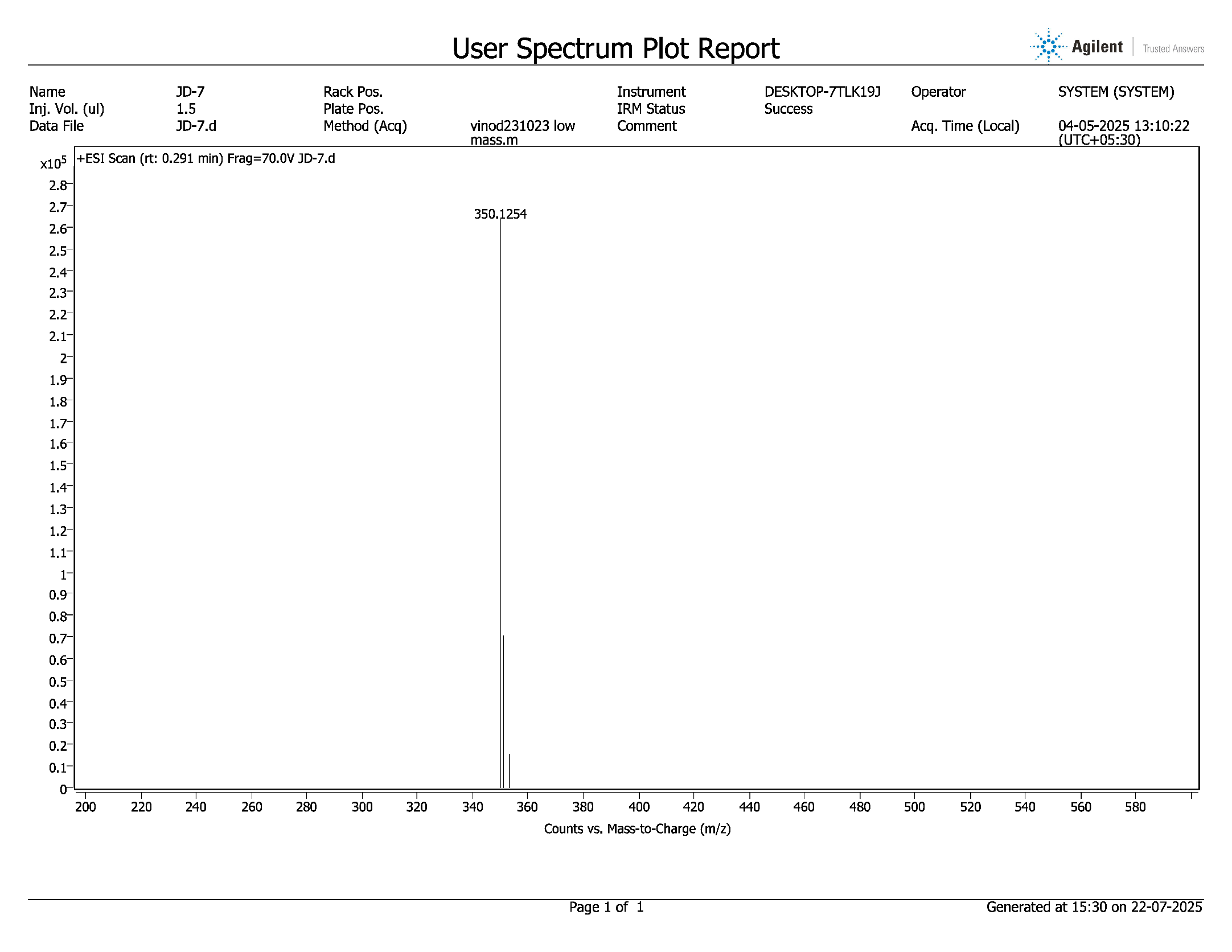


[M+H]^+^

^1^H NMR of **JRC-4**:


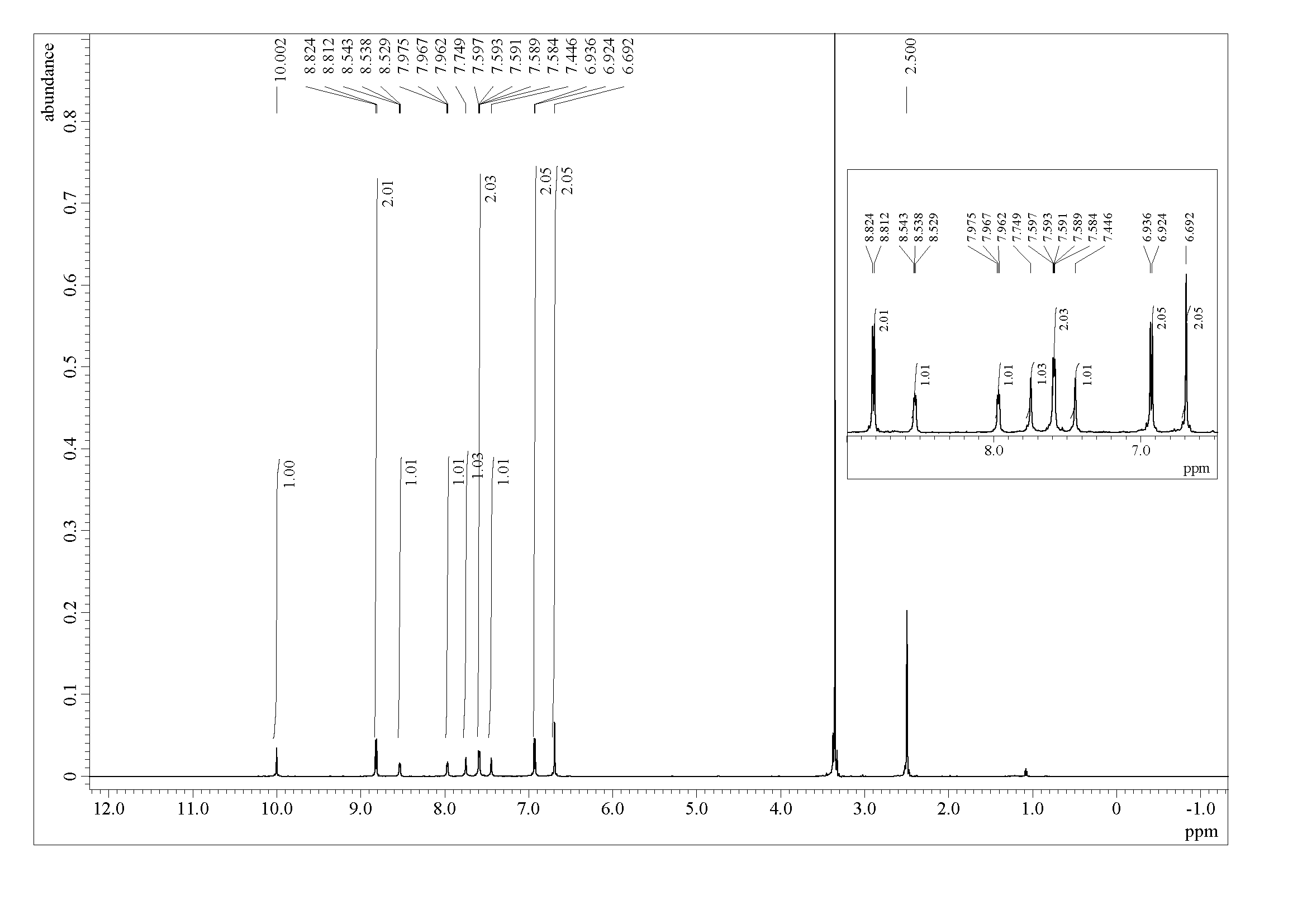


^13^C NMR of **JRC-4**:


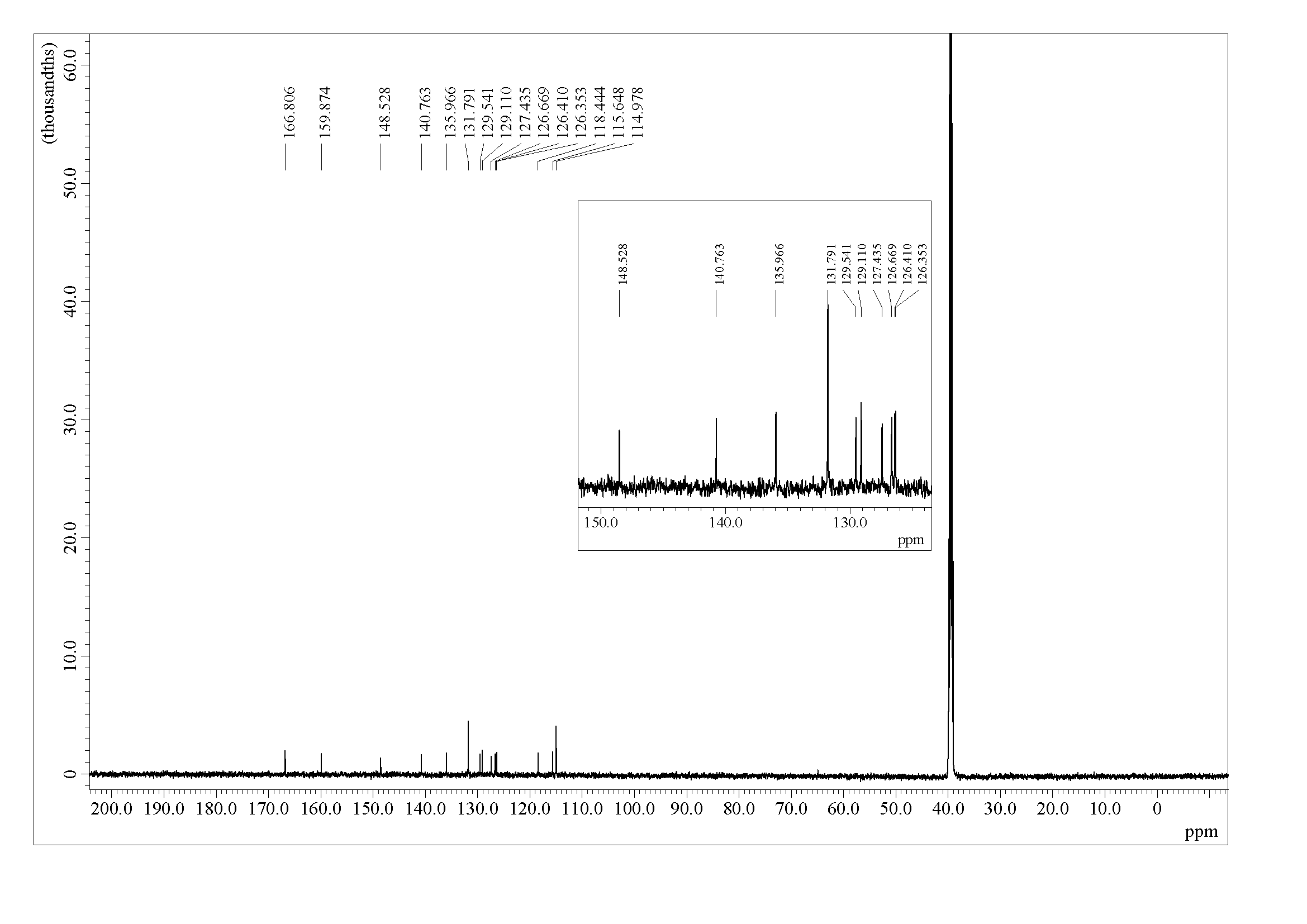


HRMS of **JRC-4**:


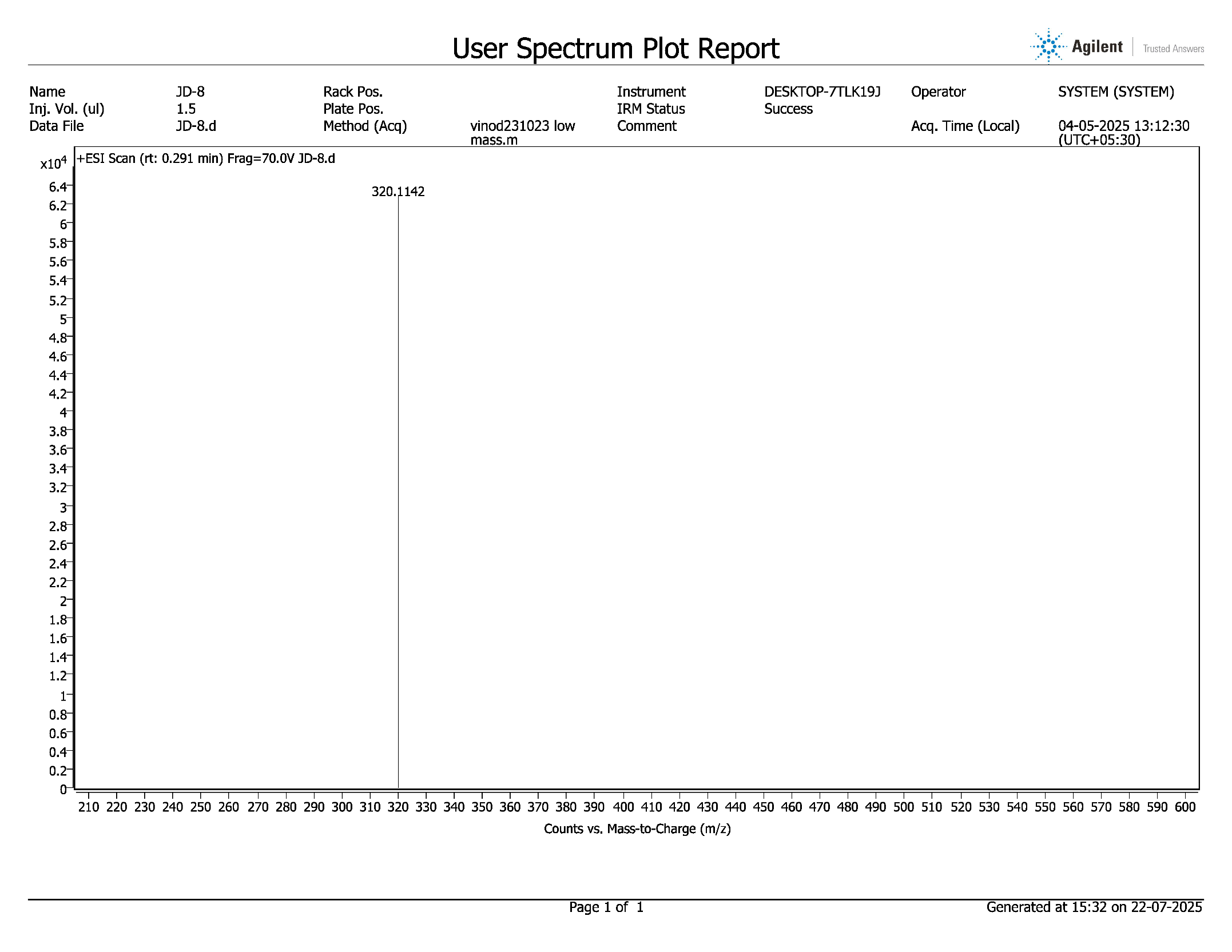


[M+H]^+^

^1^H NMR of **JRC-5**:


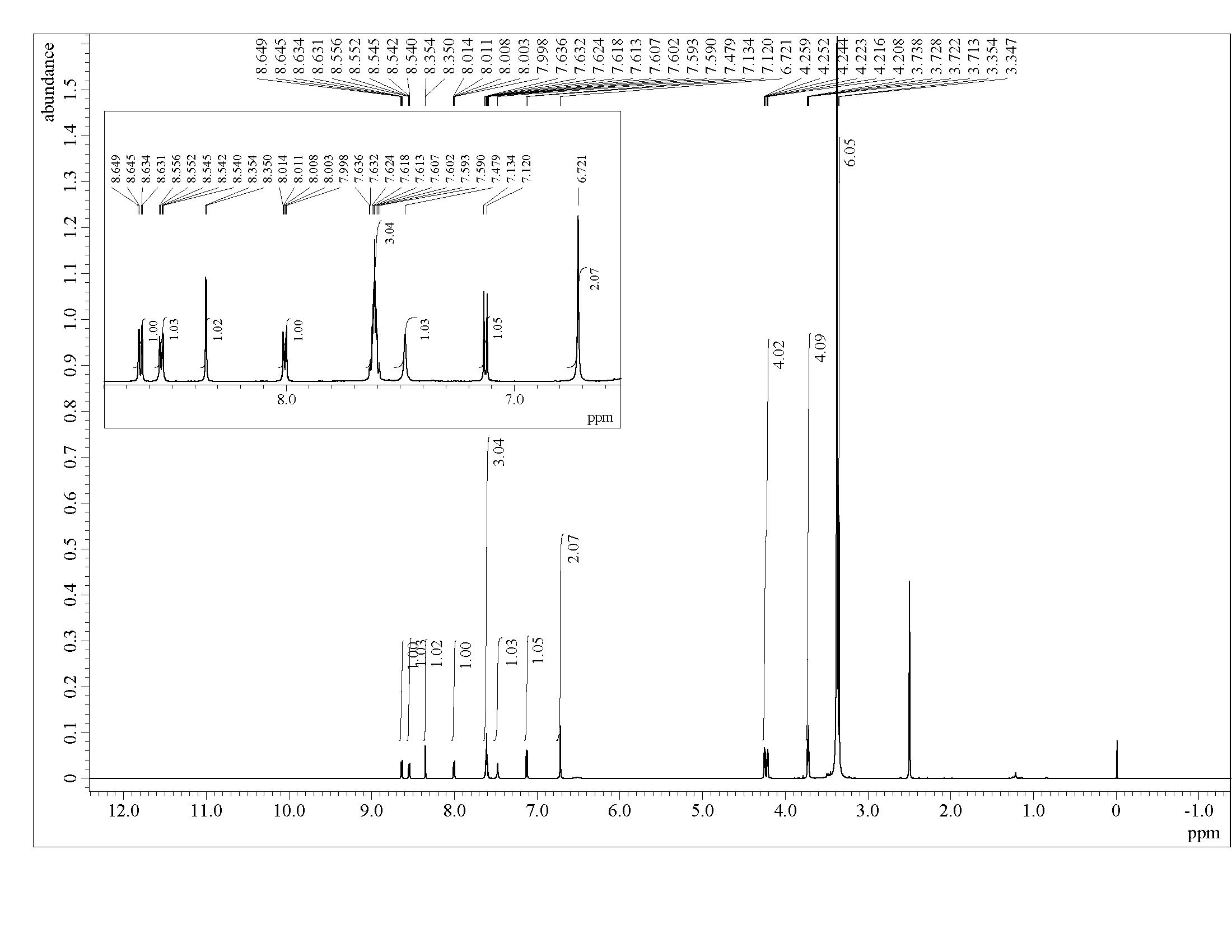


^13^C NMR of **JRC-5**:


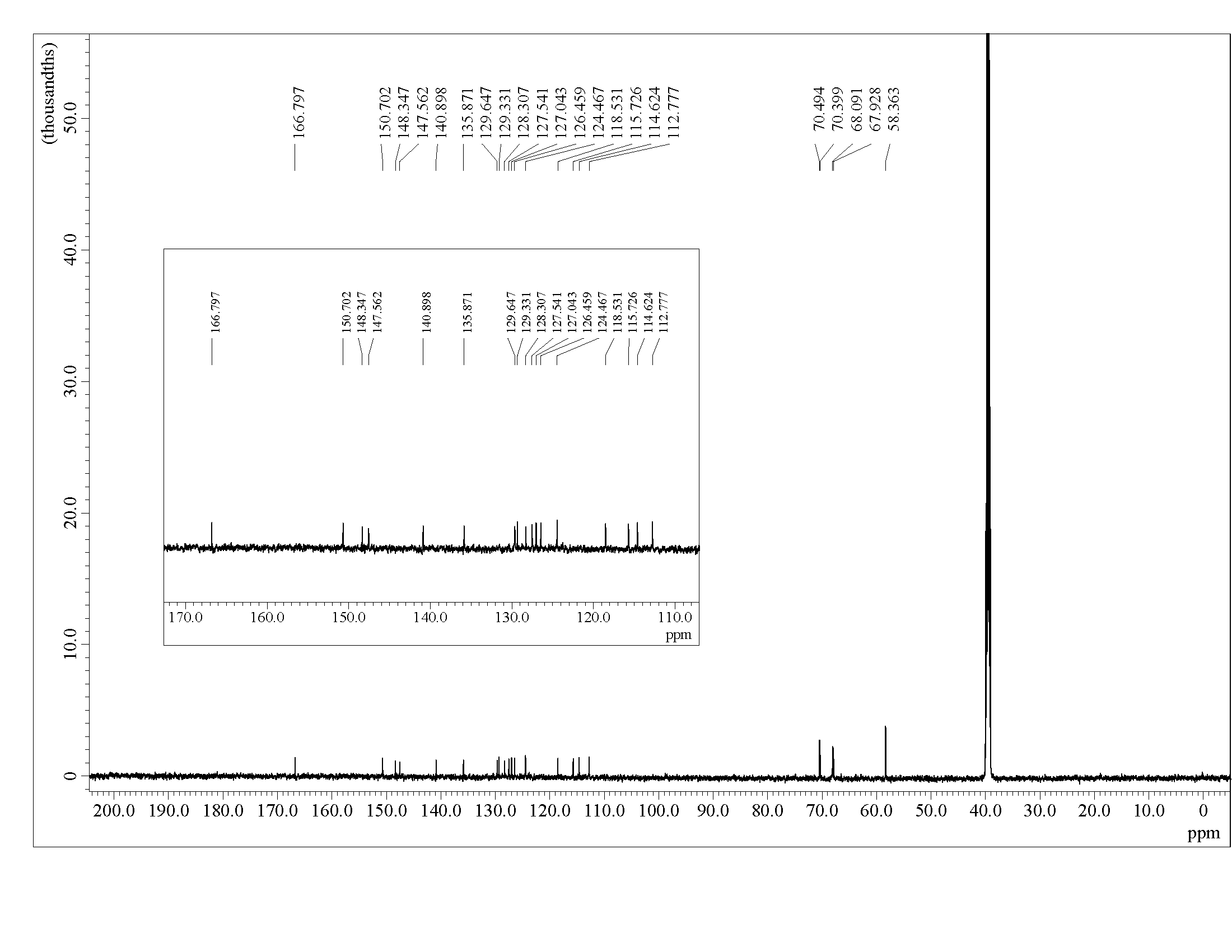


HRMS of **JRC-5**:


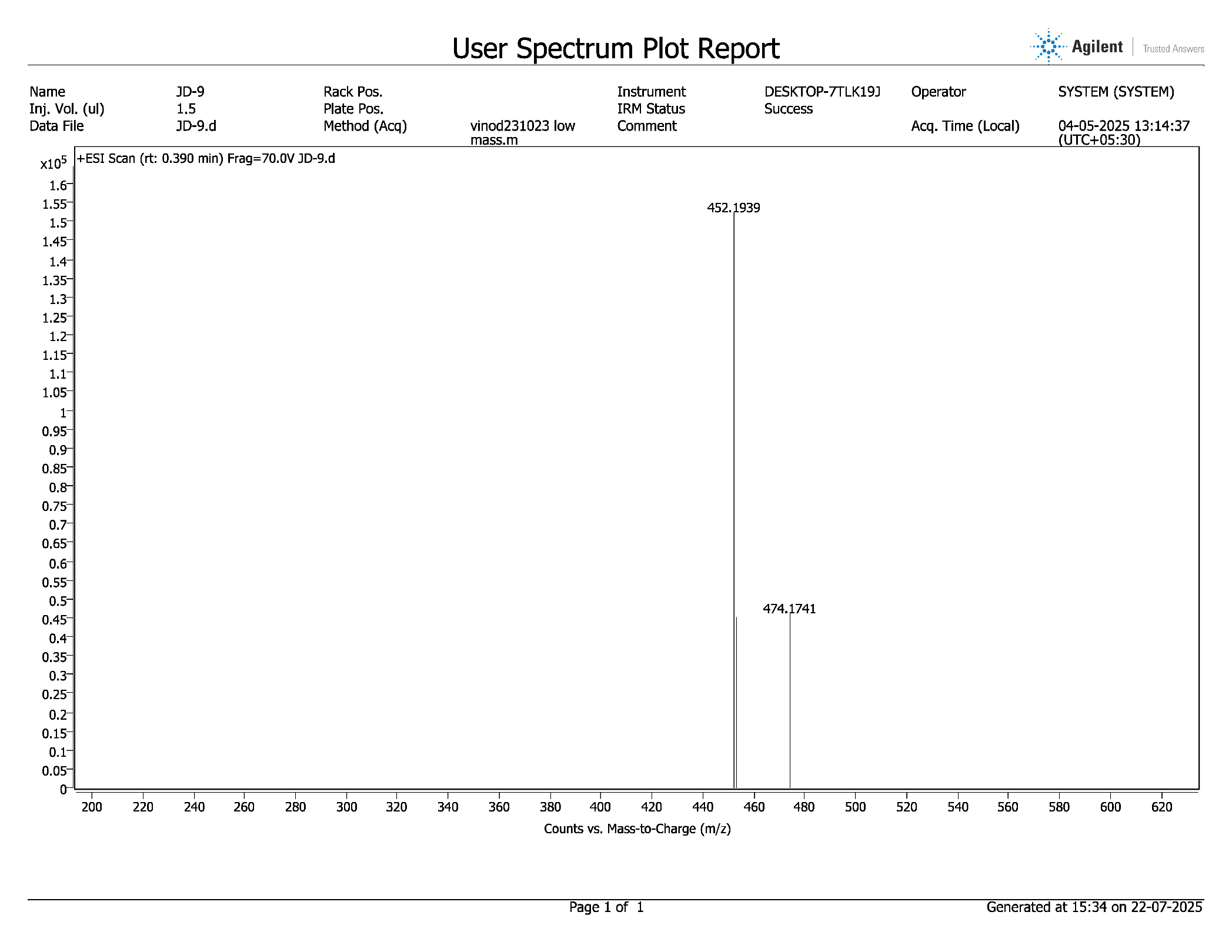


[M+H]^+^

^1^H NMR of **JRC-6**:


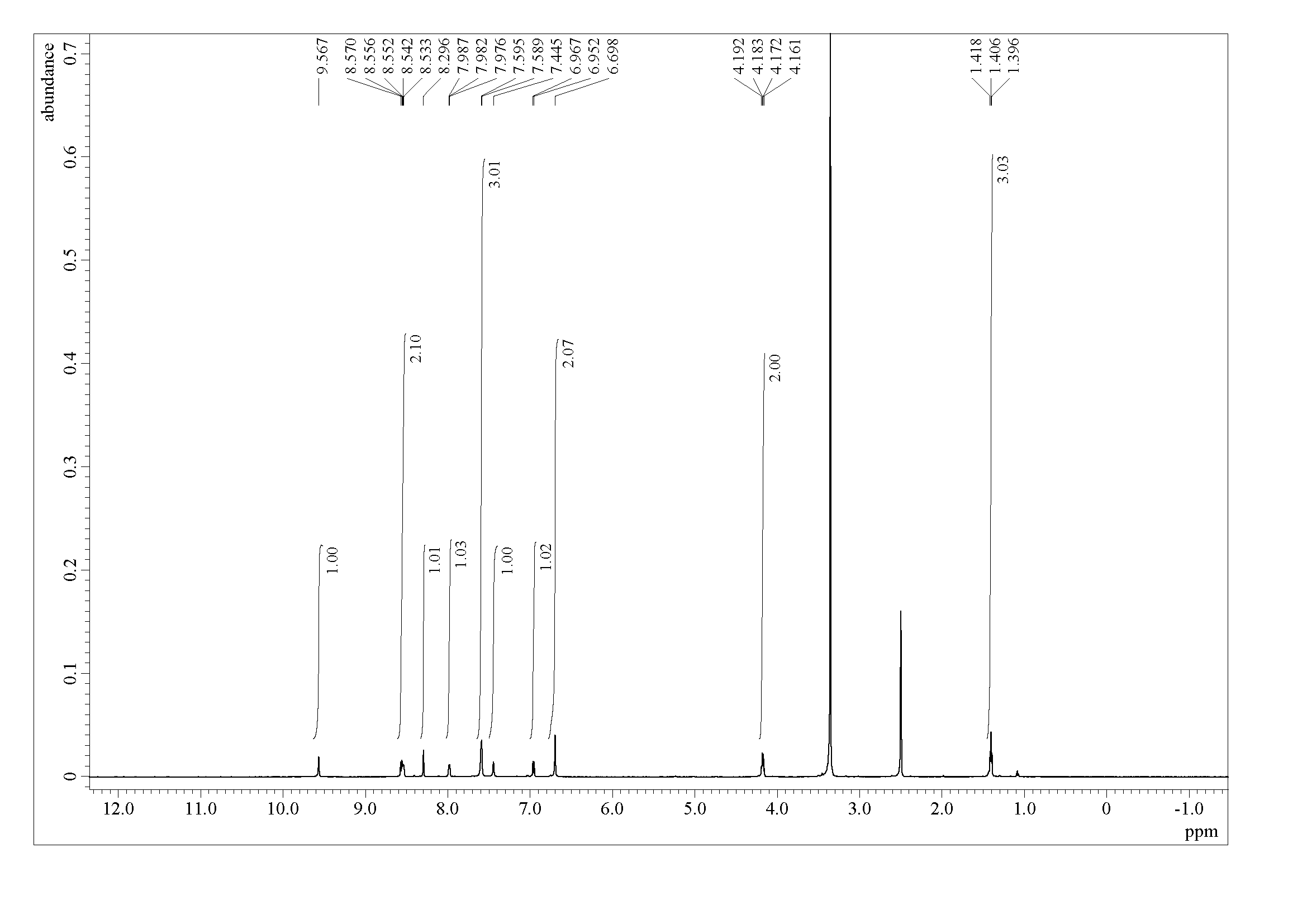


^13^C NMR of **JRC-6**:


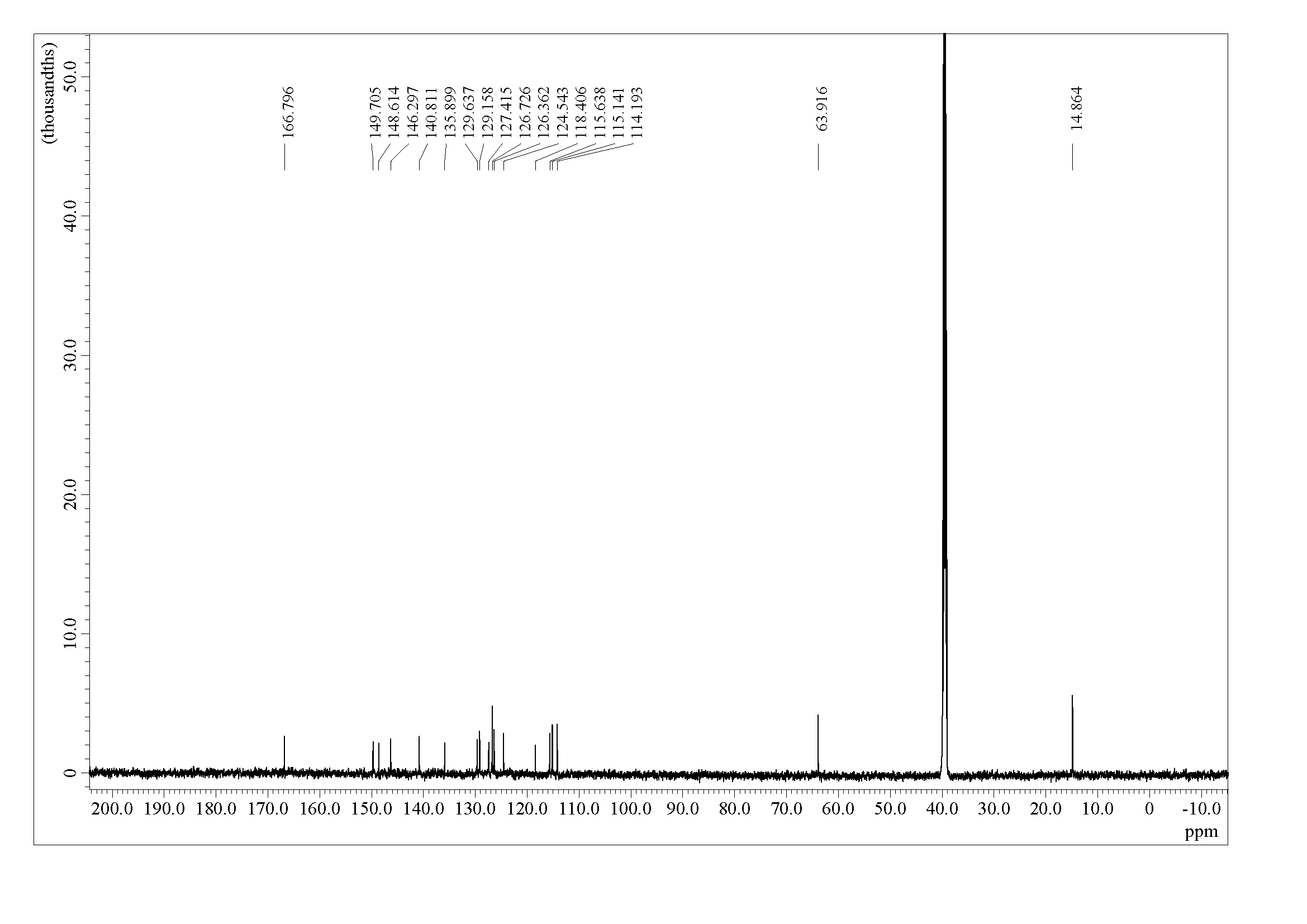


HRMS of **JRC-6:**


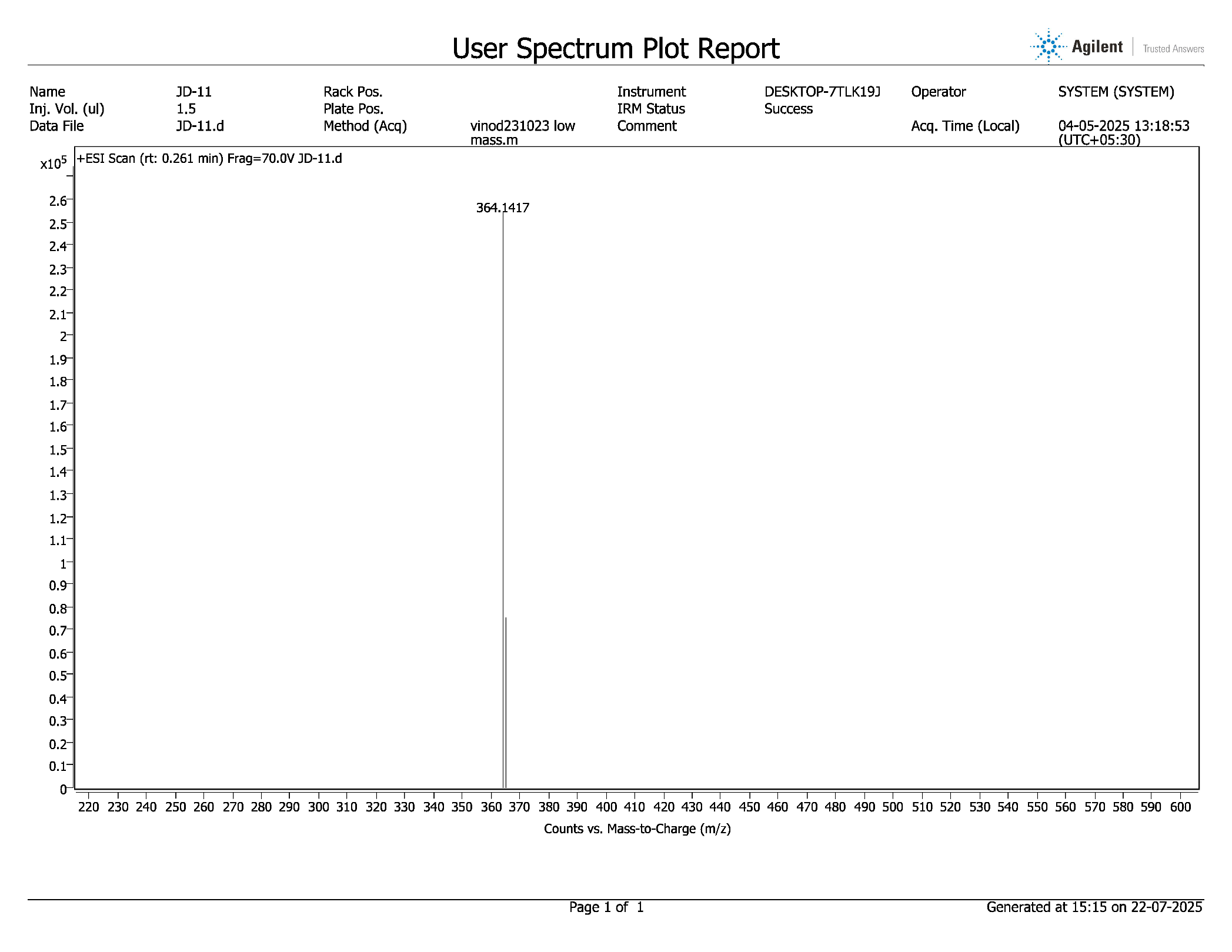


[M+H]^+^

^1^H NMR of **JRC-7**:


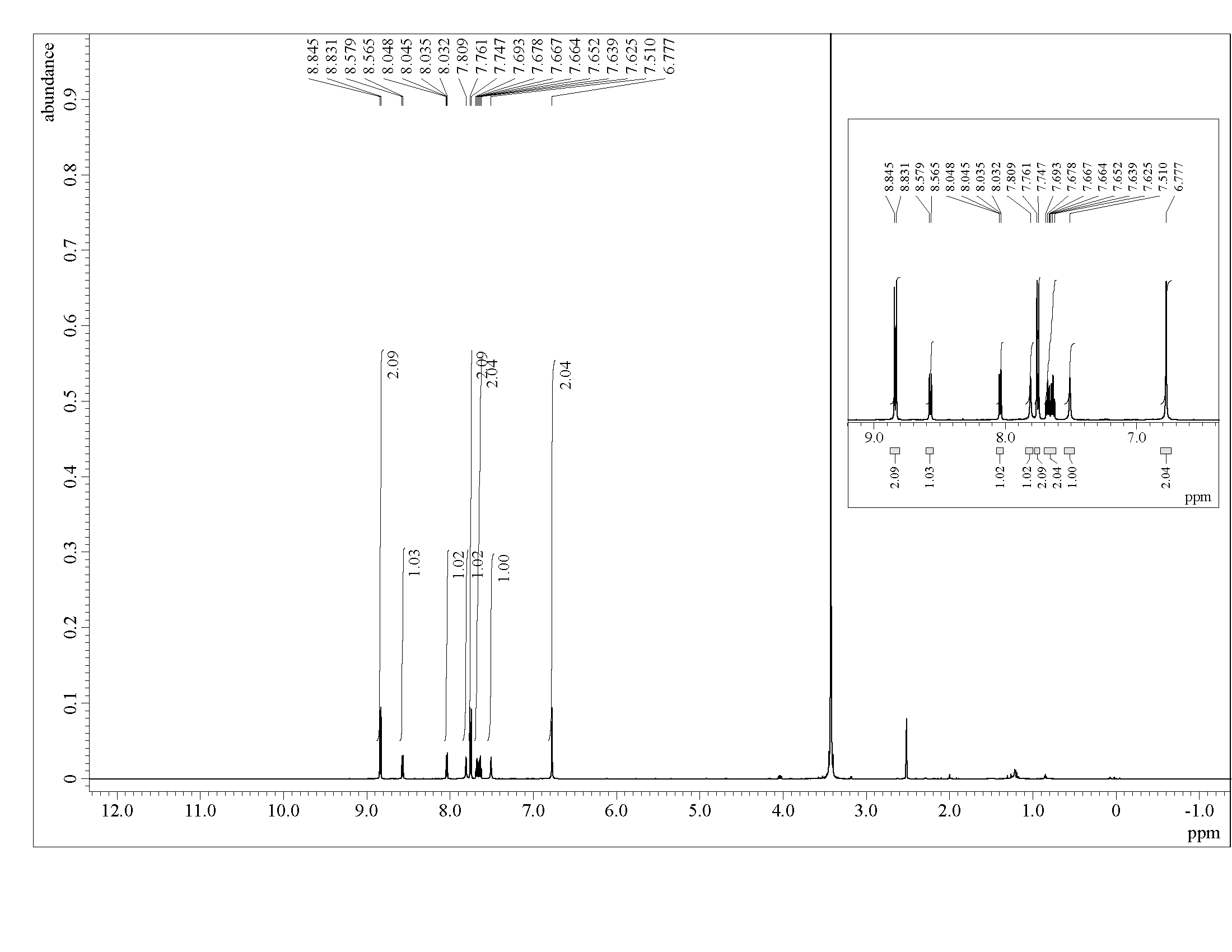


^13^C NMR of **JRC-7**:


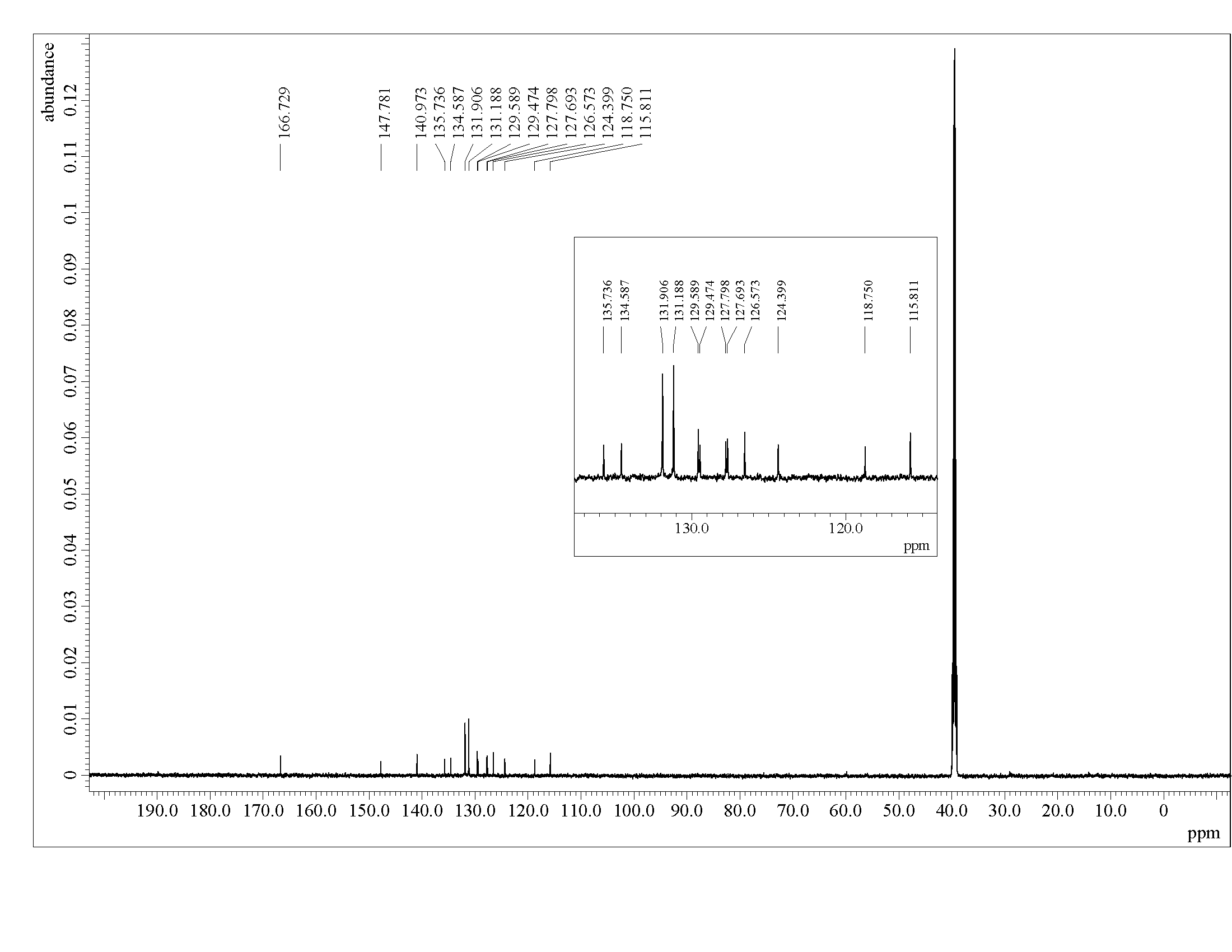


HRMS of **JRC-7**:


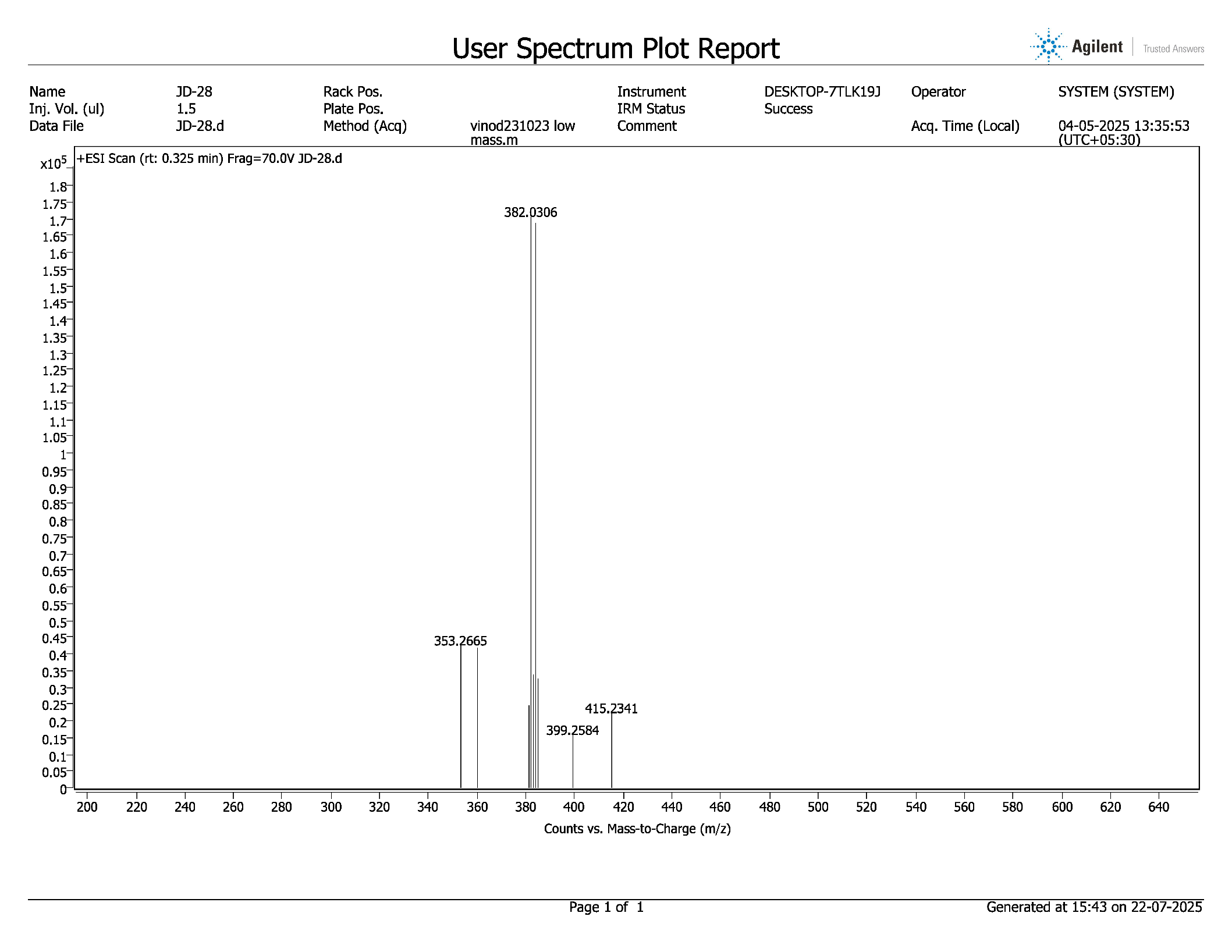


[M+H]^+^

^1^H NMR of **JRC-8**:


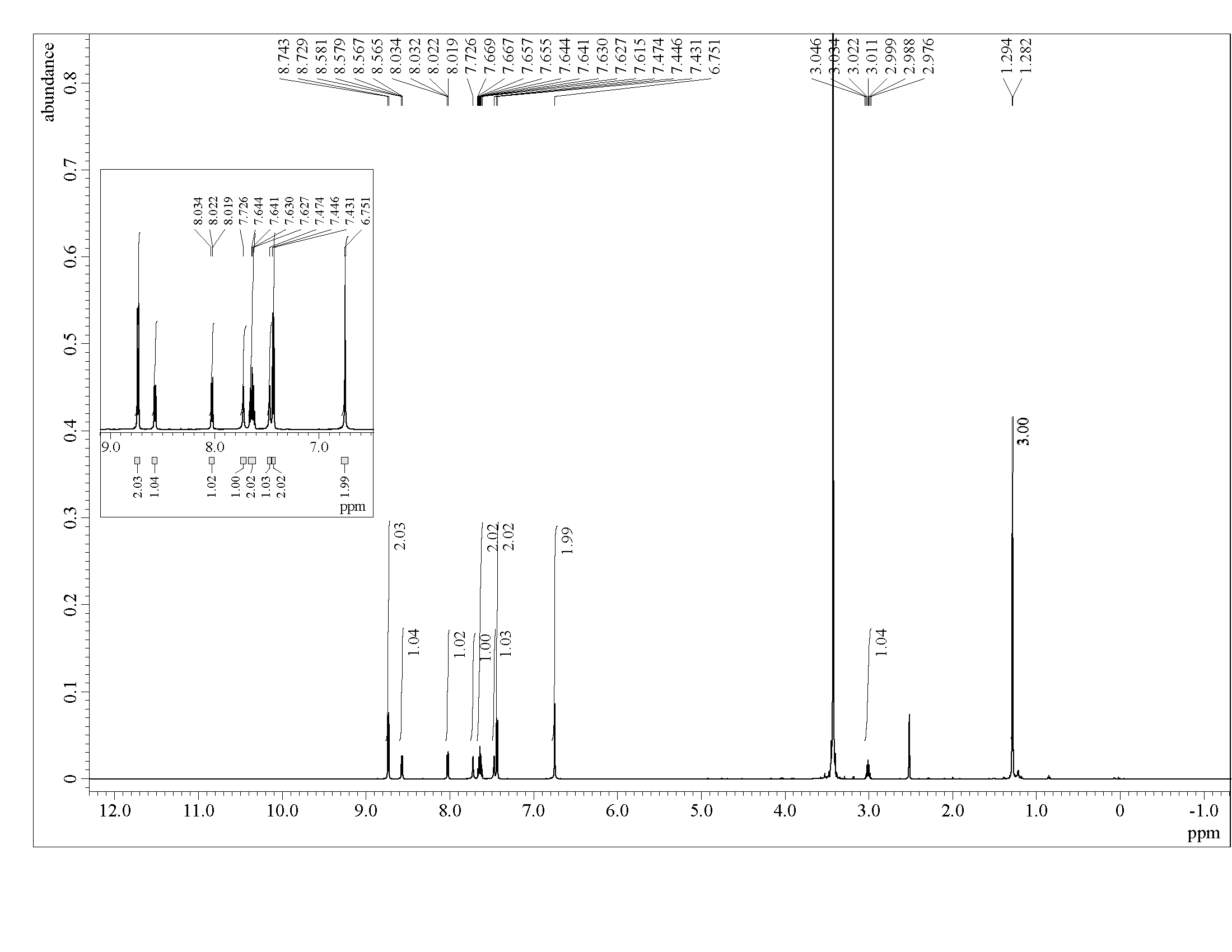


^13^C NMR of **JRC-8**:


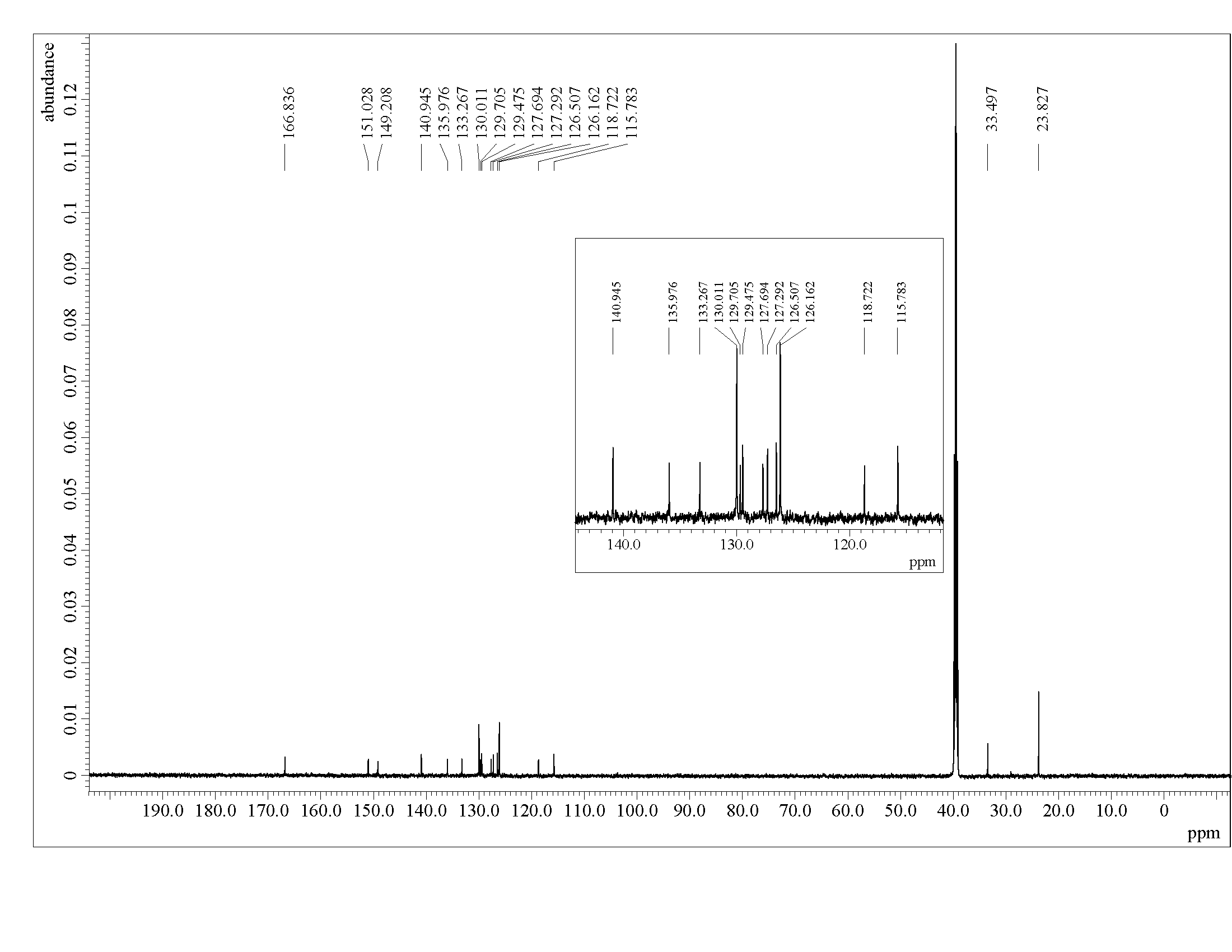


HRMS of **JRC-8**:


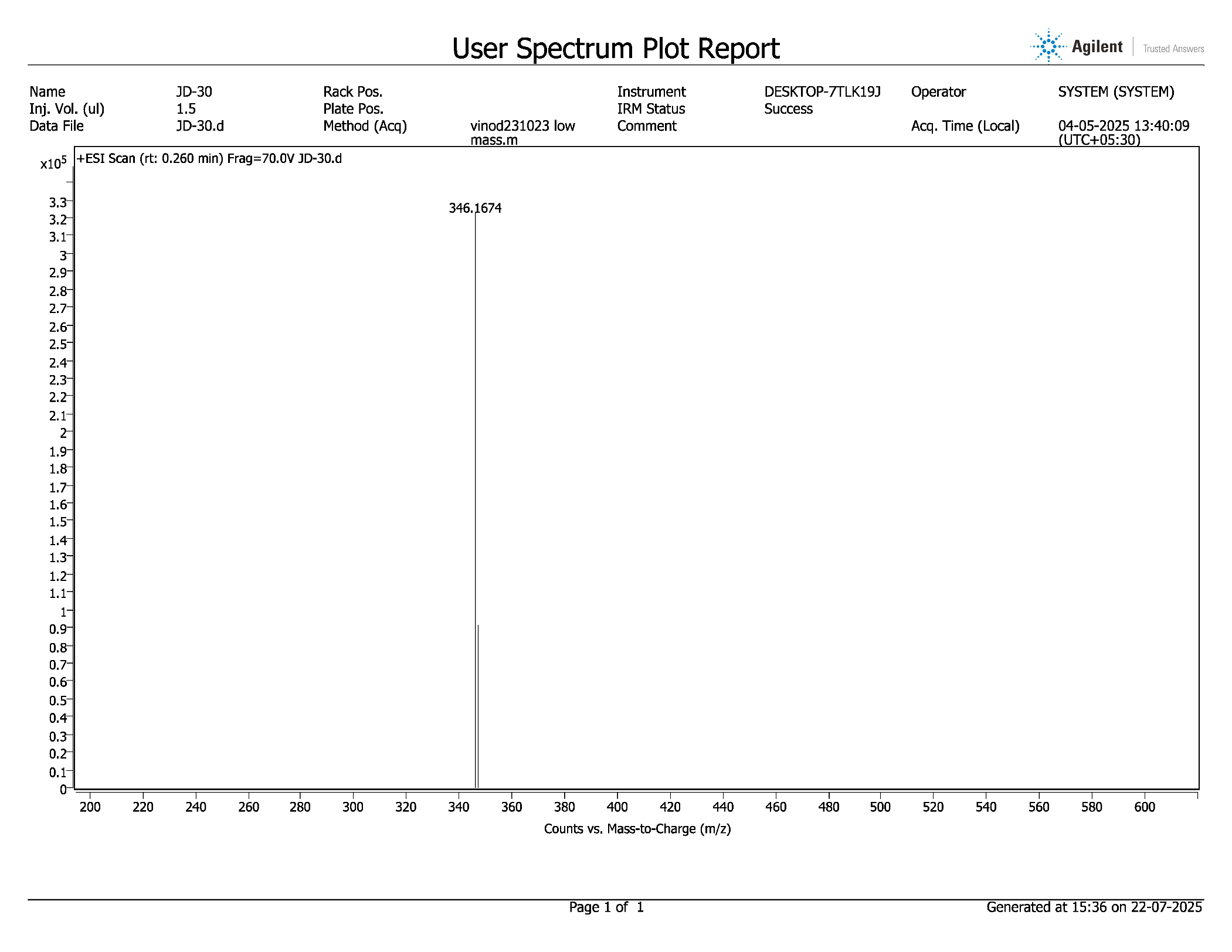


[M+H]^+^

^1^H NMR of **JRC-9**:


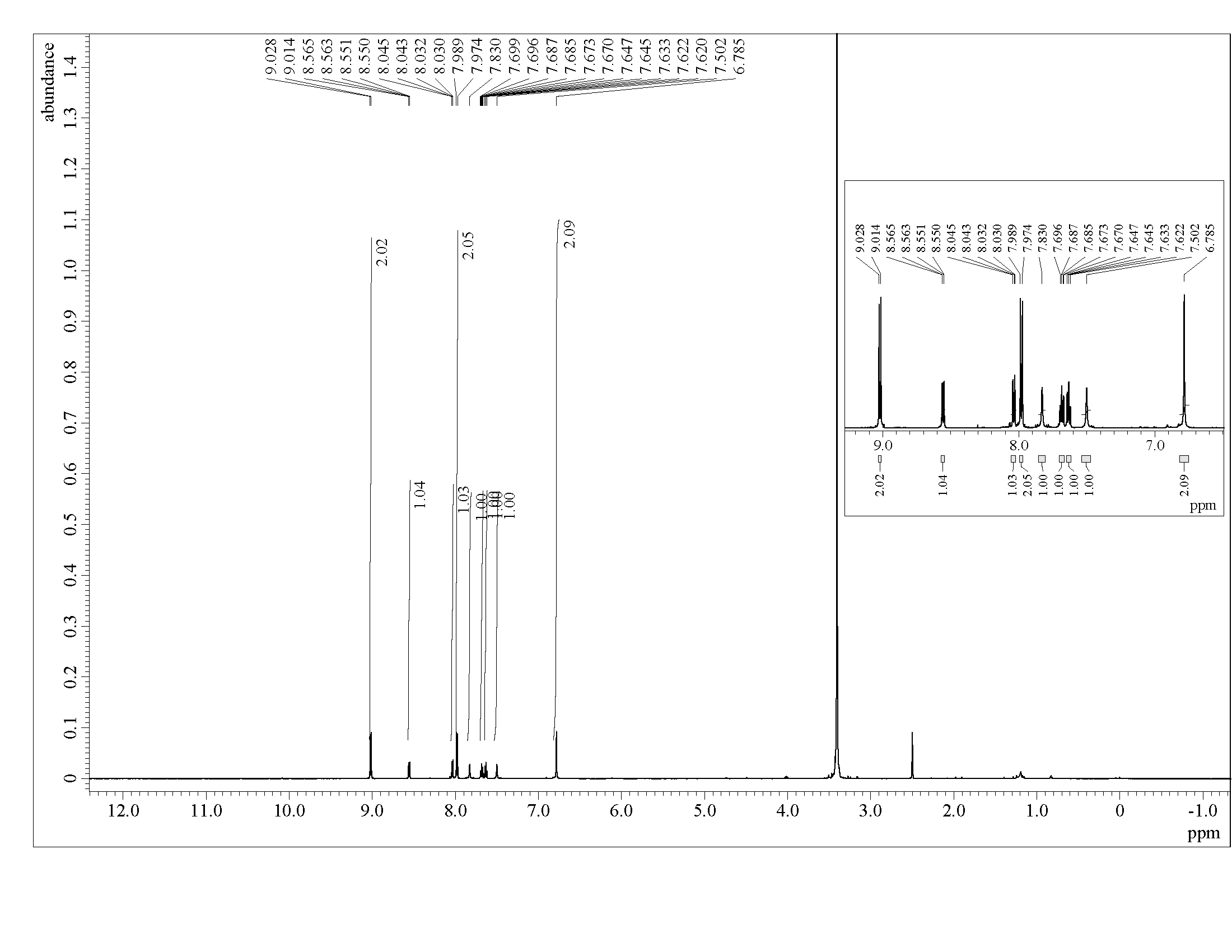


^13^C NMR of **JRC-9**:


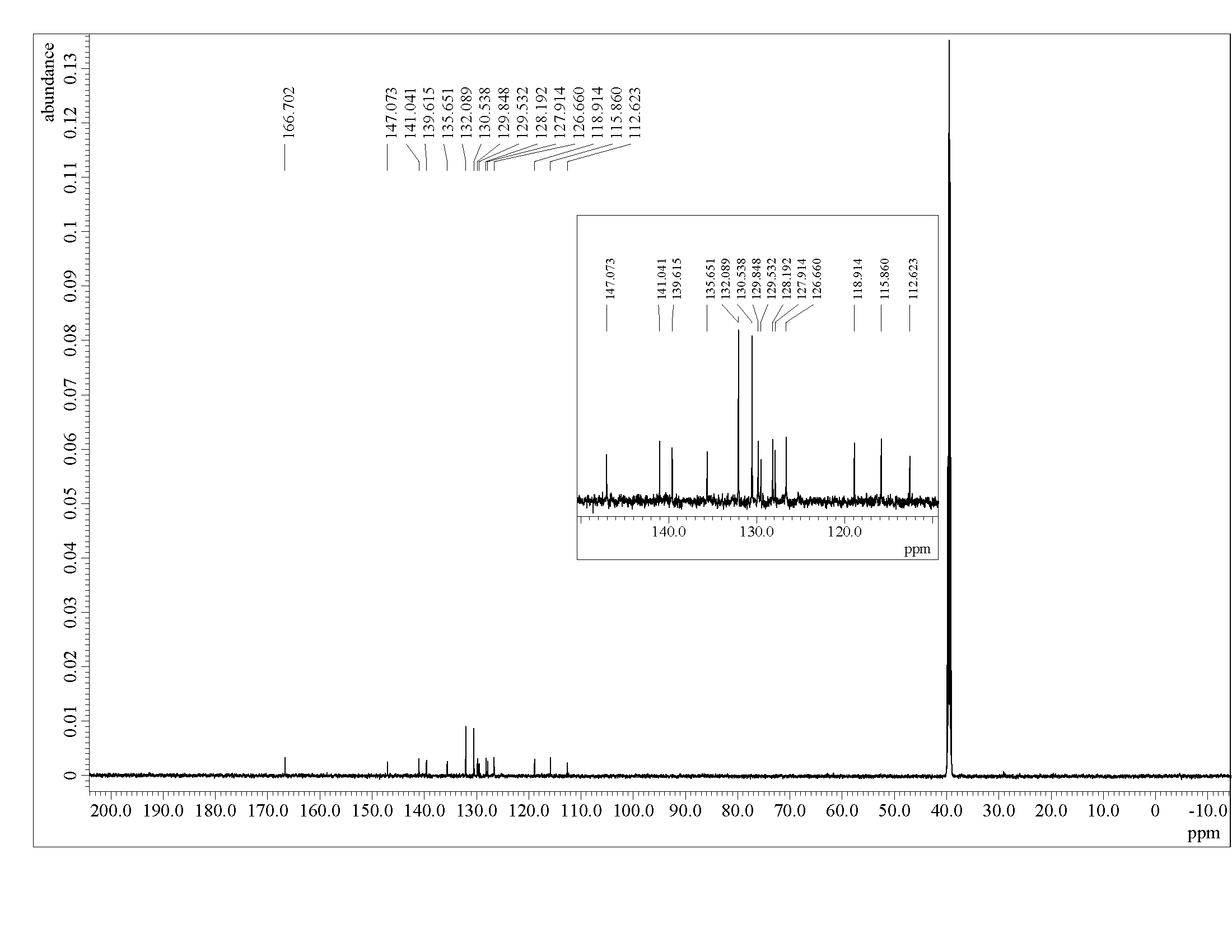


HRMS of **JRC-9**:


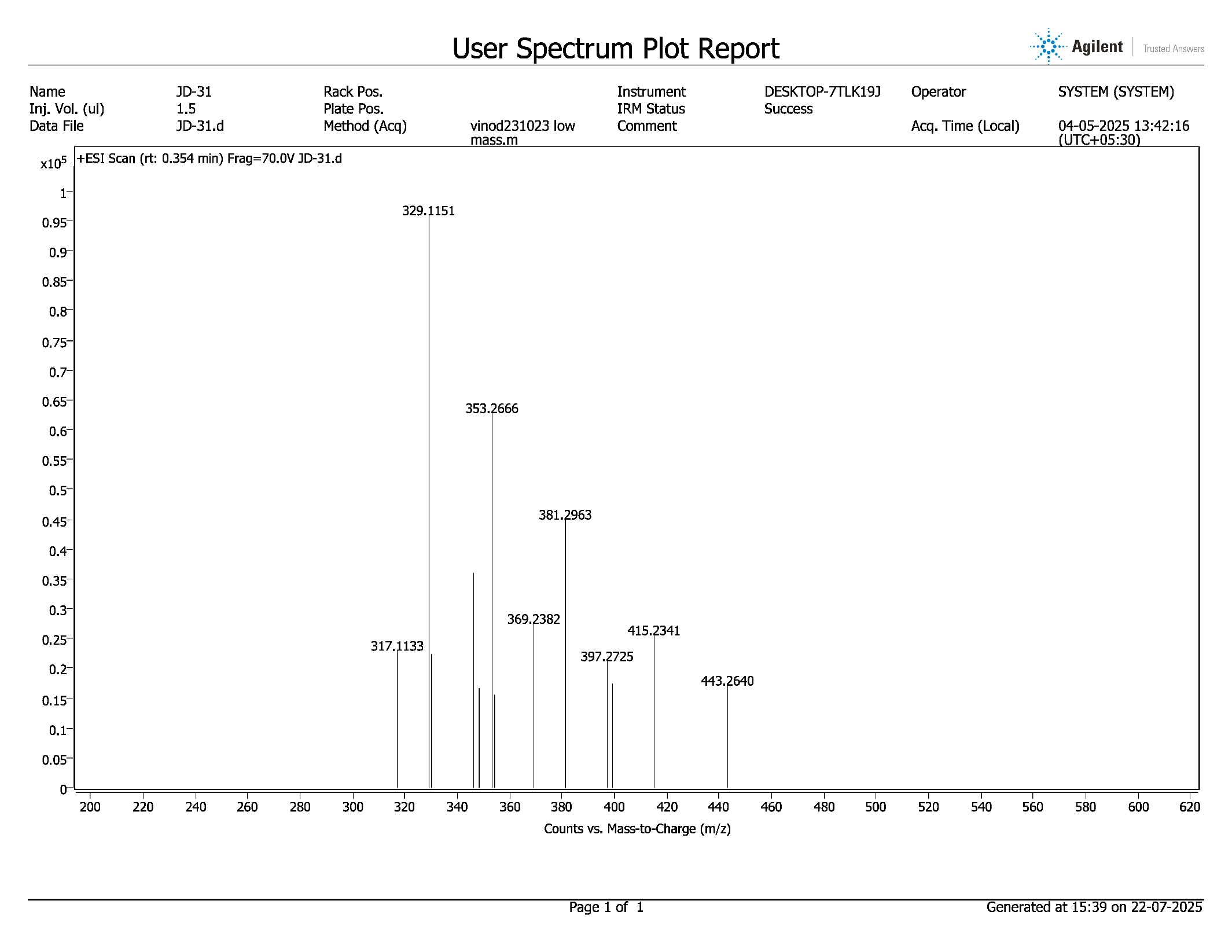


[M+H]^+^

^1^H NMR of **JRC-10**:


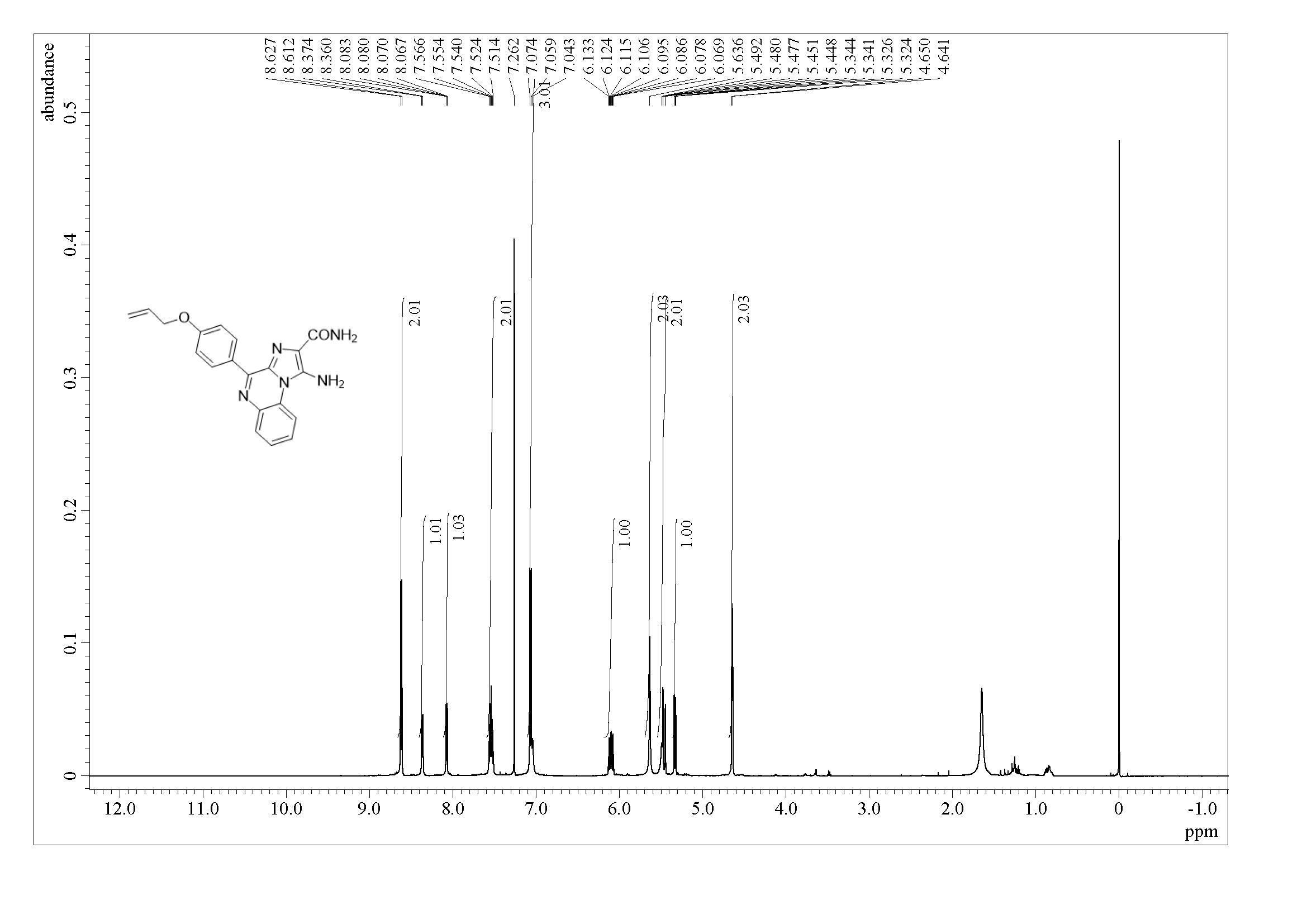


^13^C NMR of **JRC-10**:


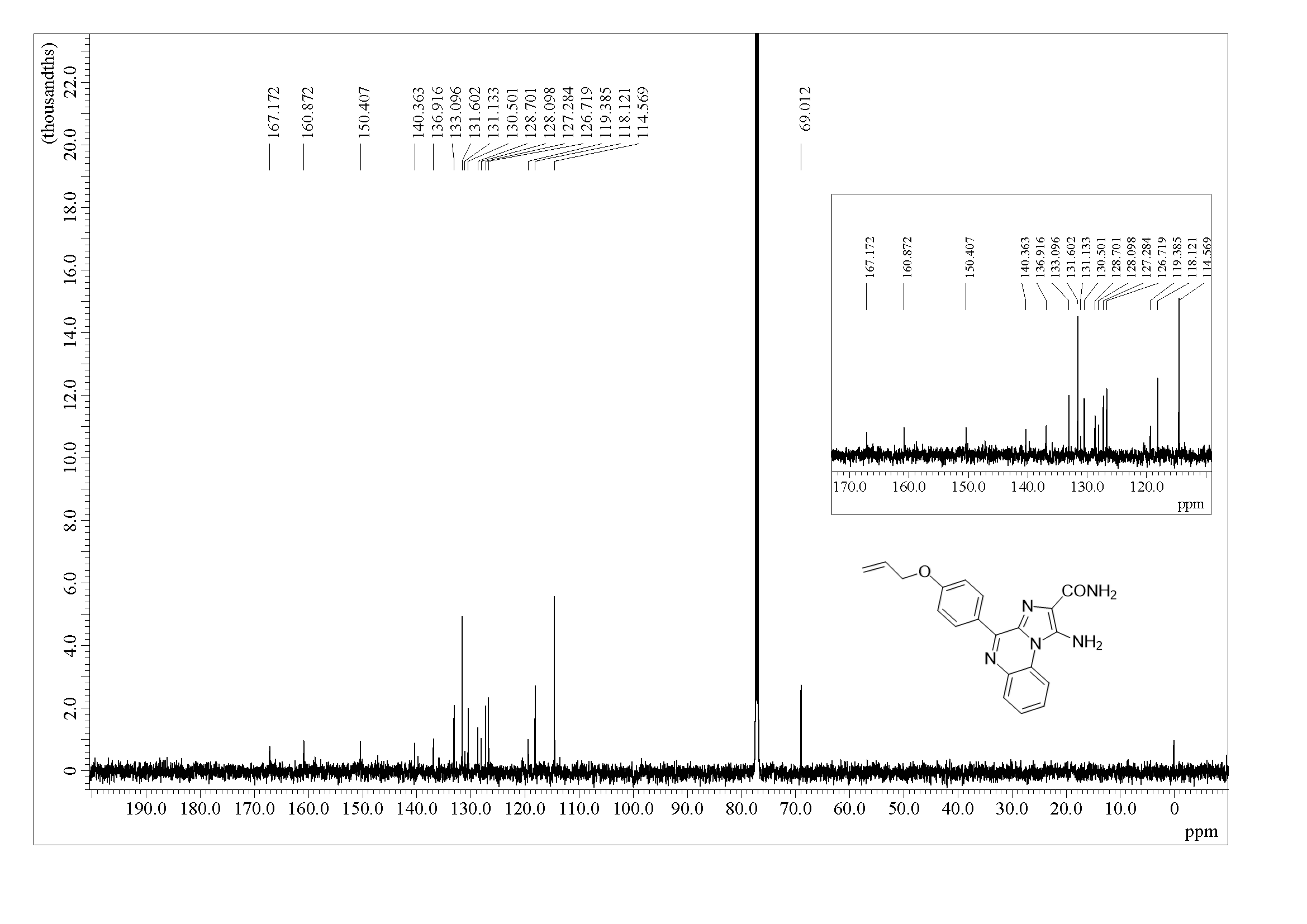


HRMS of **JRC-10**:


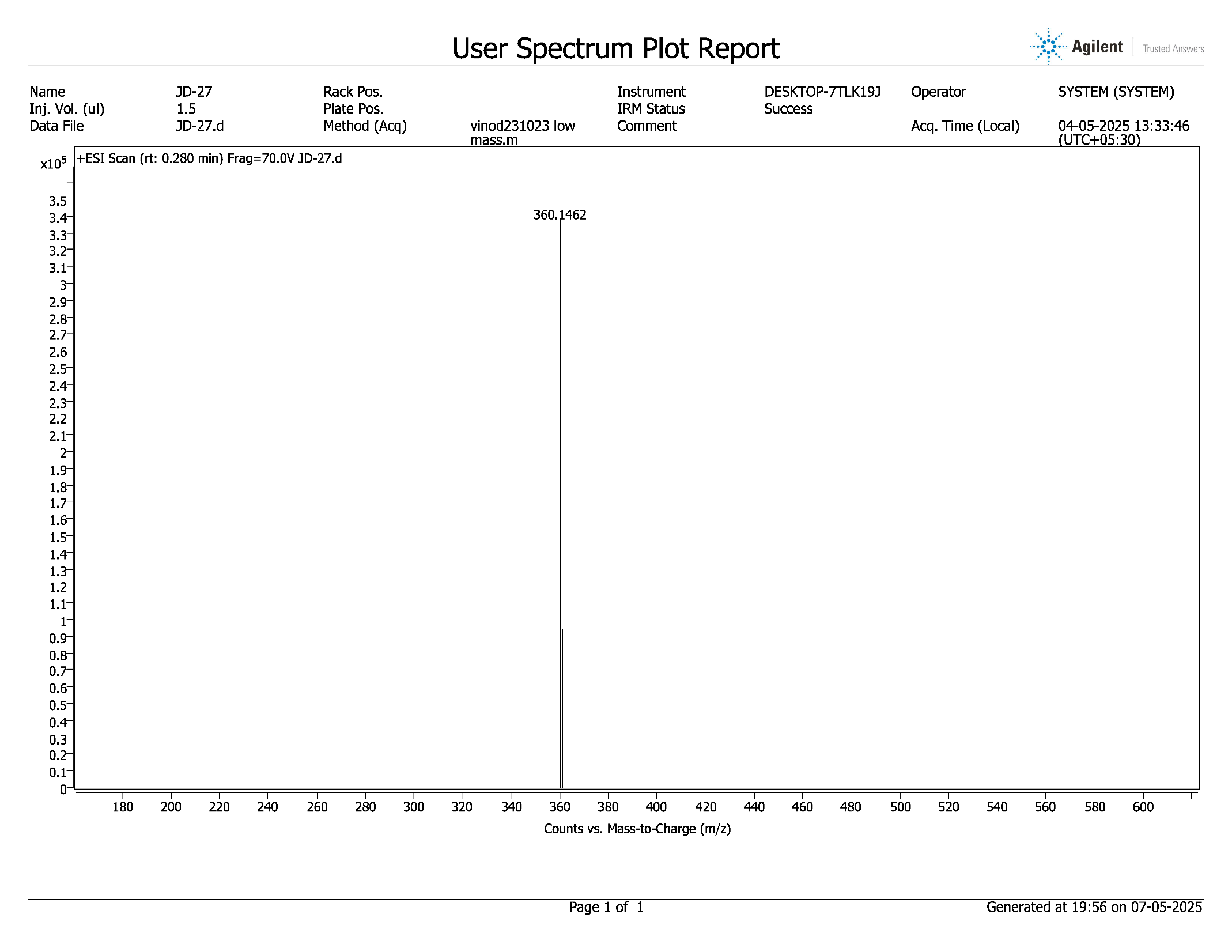


[M+H]^+^

^1^H NMR of **JRC-11**:


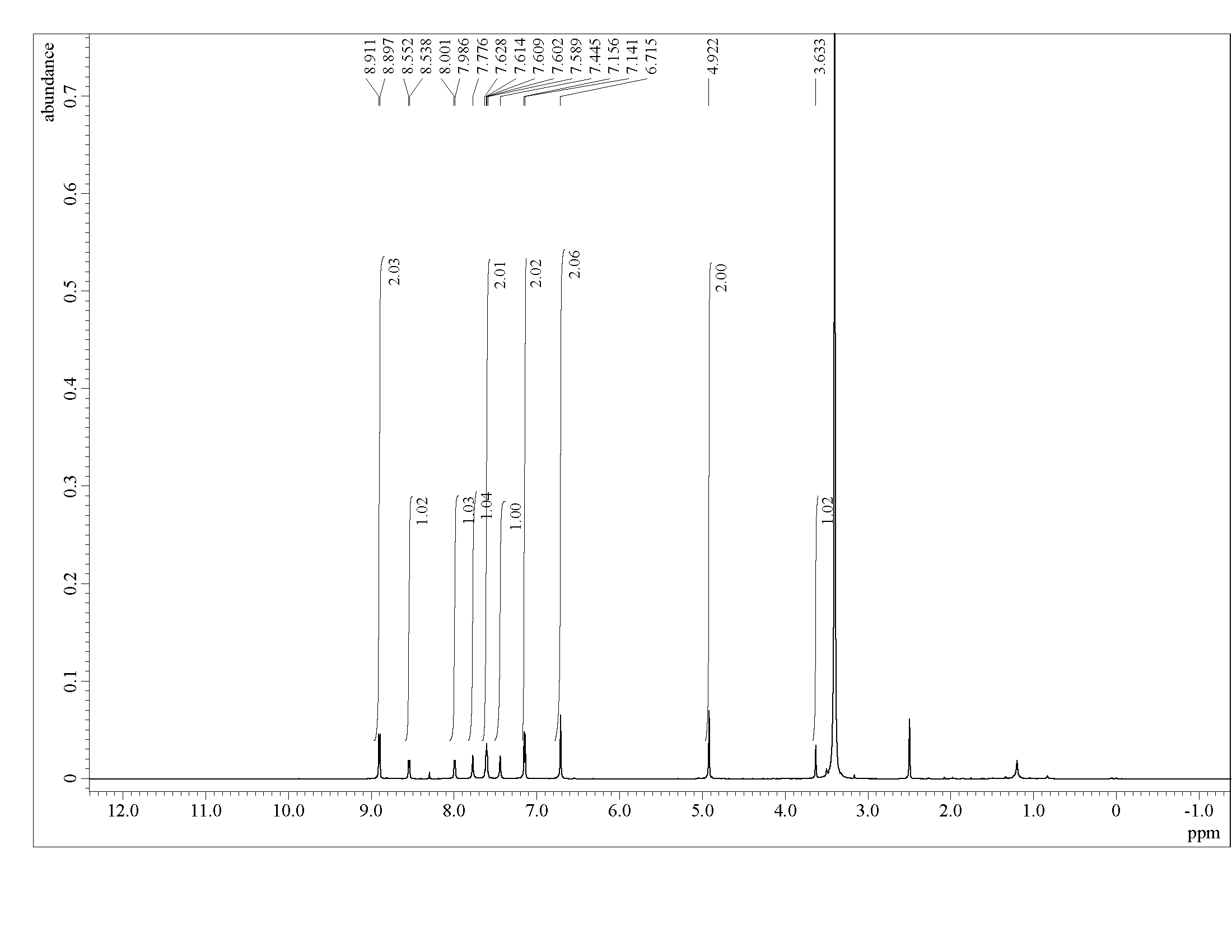


^13^C NMR of **JRC-11**:


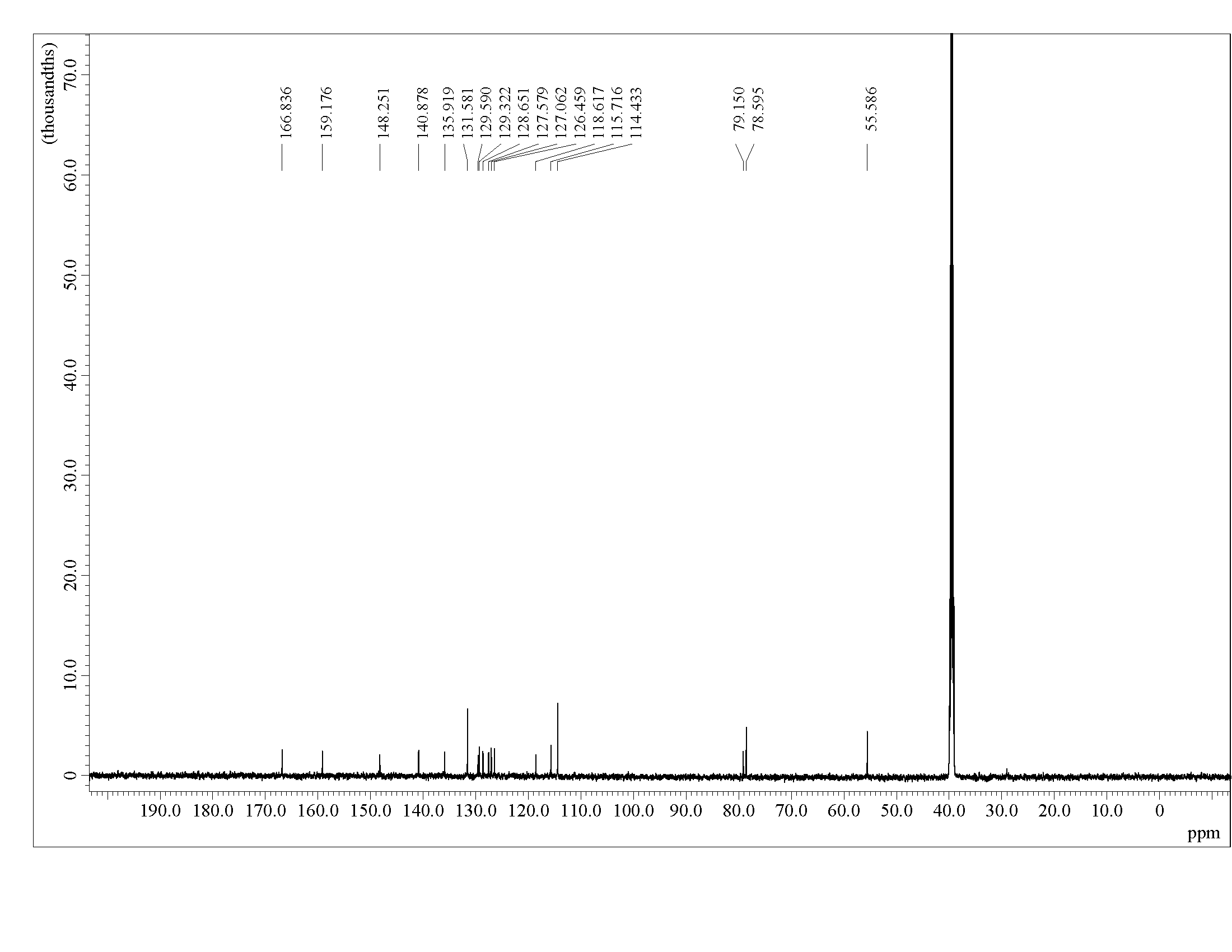


HRMS of **JRC-11**:


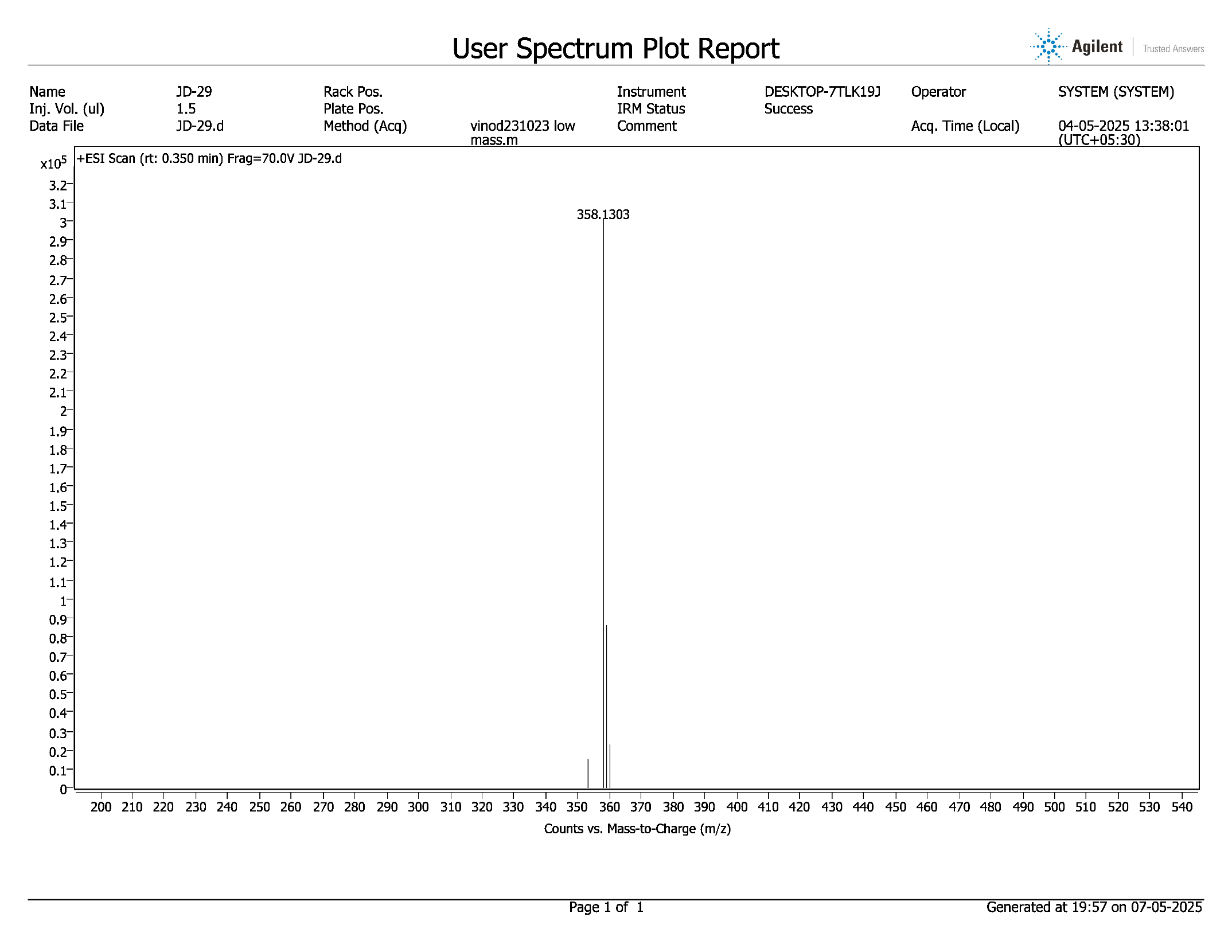


[M+H]^+^

^1^H NMR of **JRC-12**:


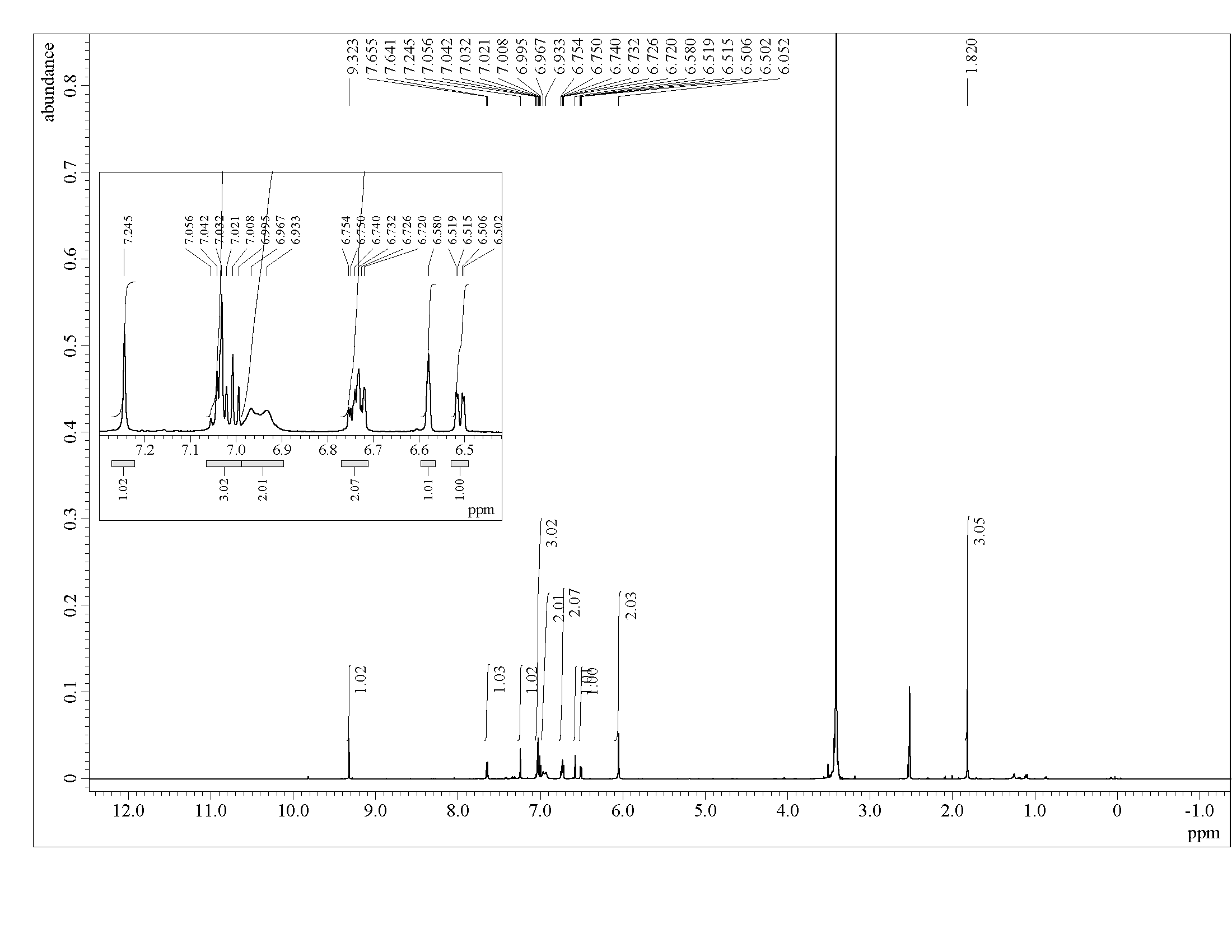


^13^C NMR of **JRC-12**:


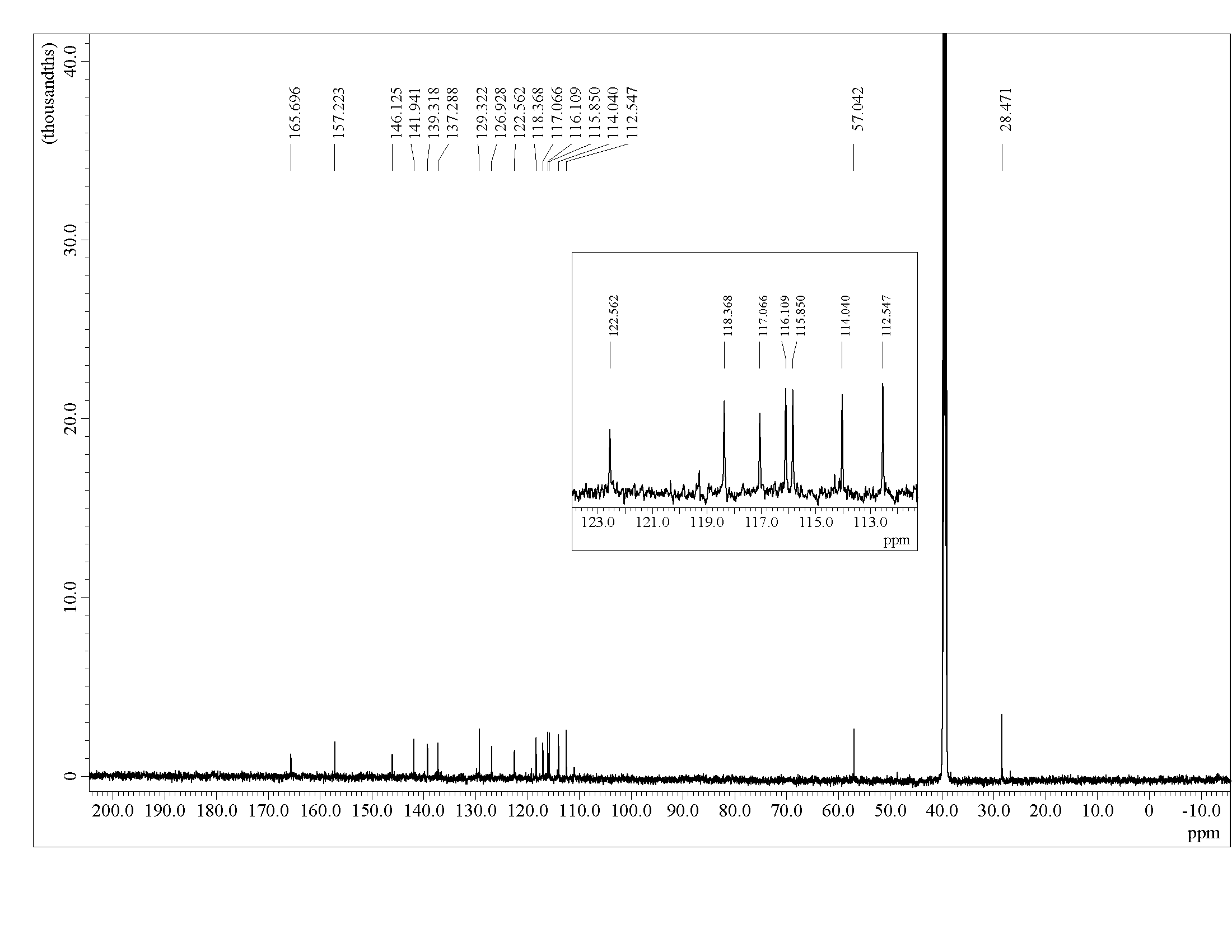


HRMS of **JRC-12**:


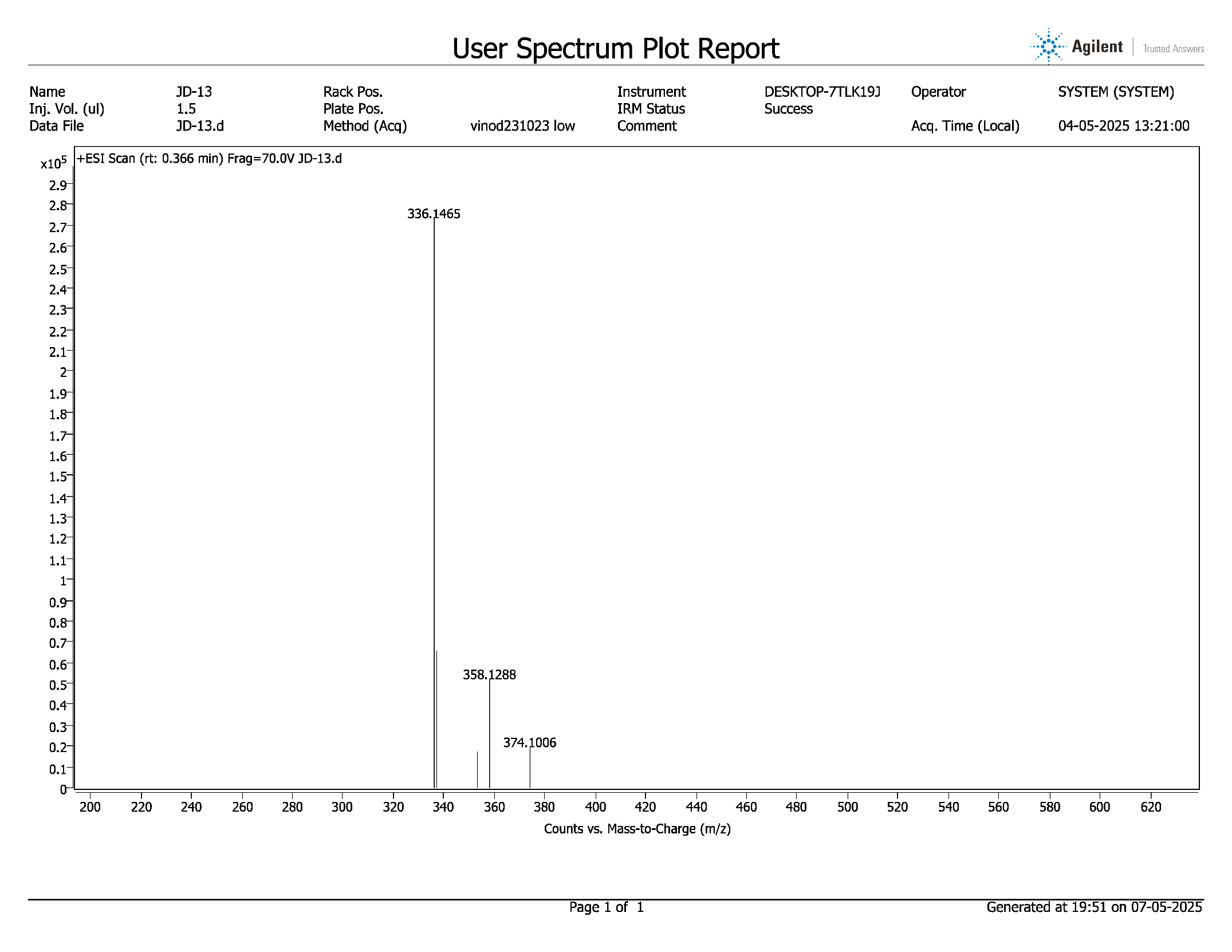


[M+H]^+^

^1^H NMR of **JRC-13**:


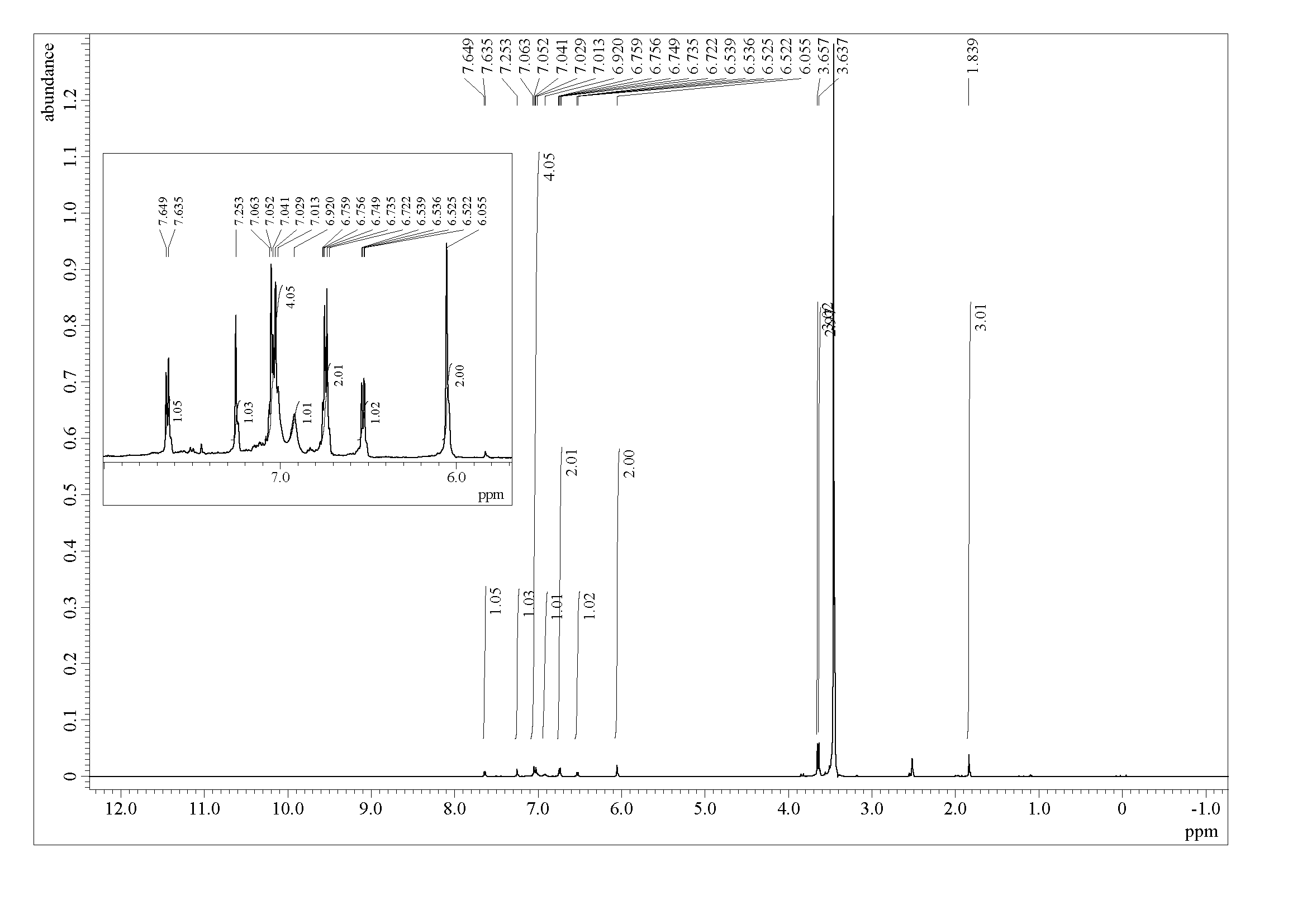


^13^C NMR of **JRC-13**:


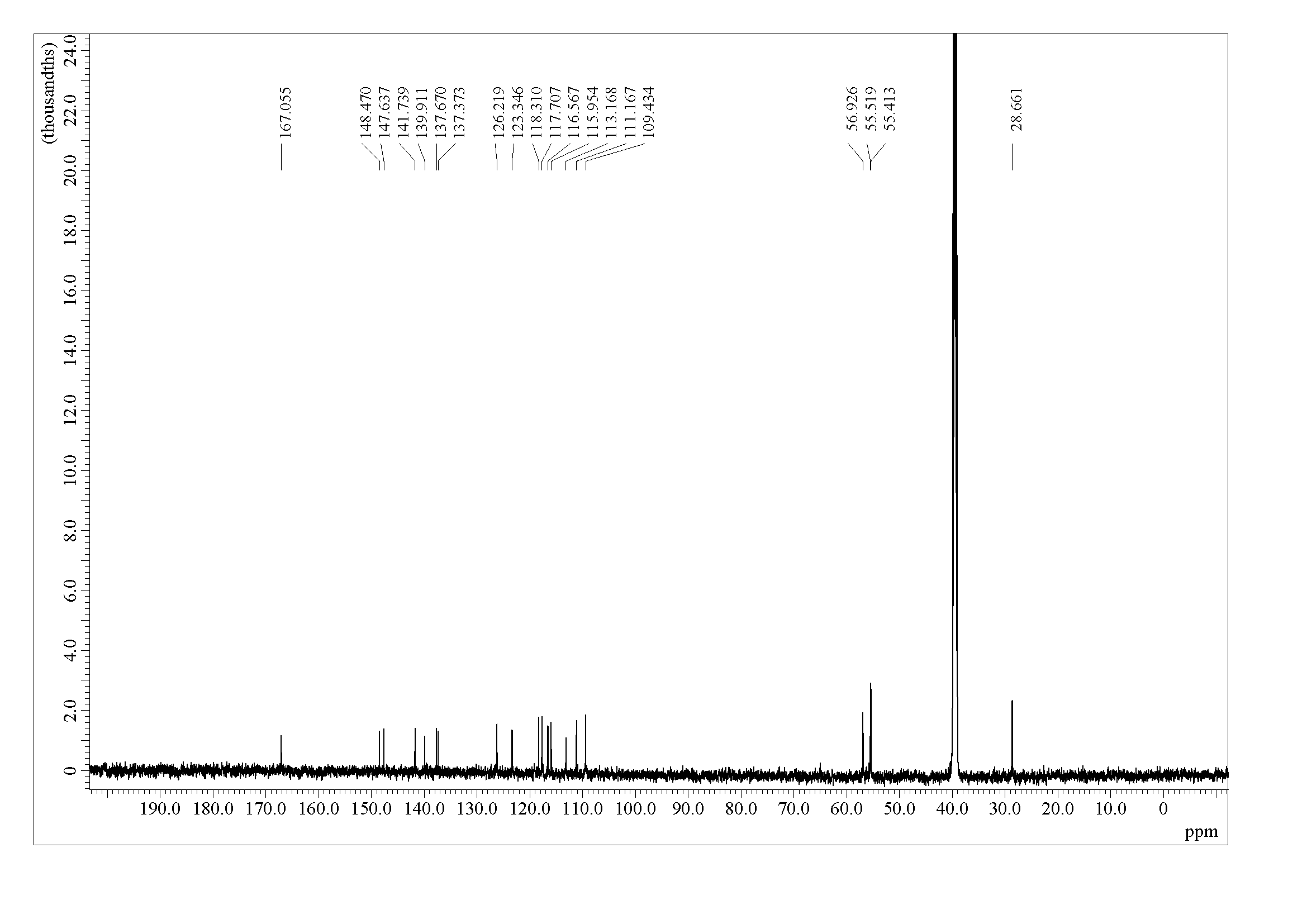


HRMS of **JRC-13**:


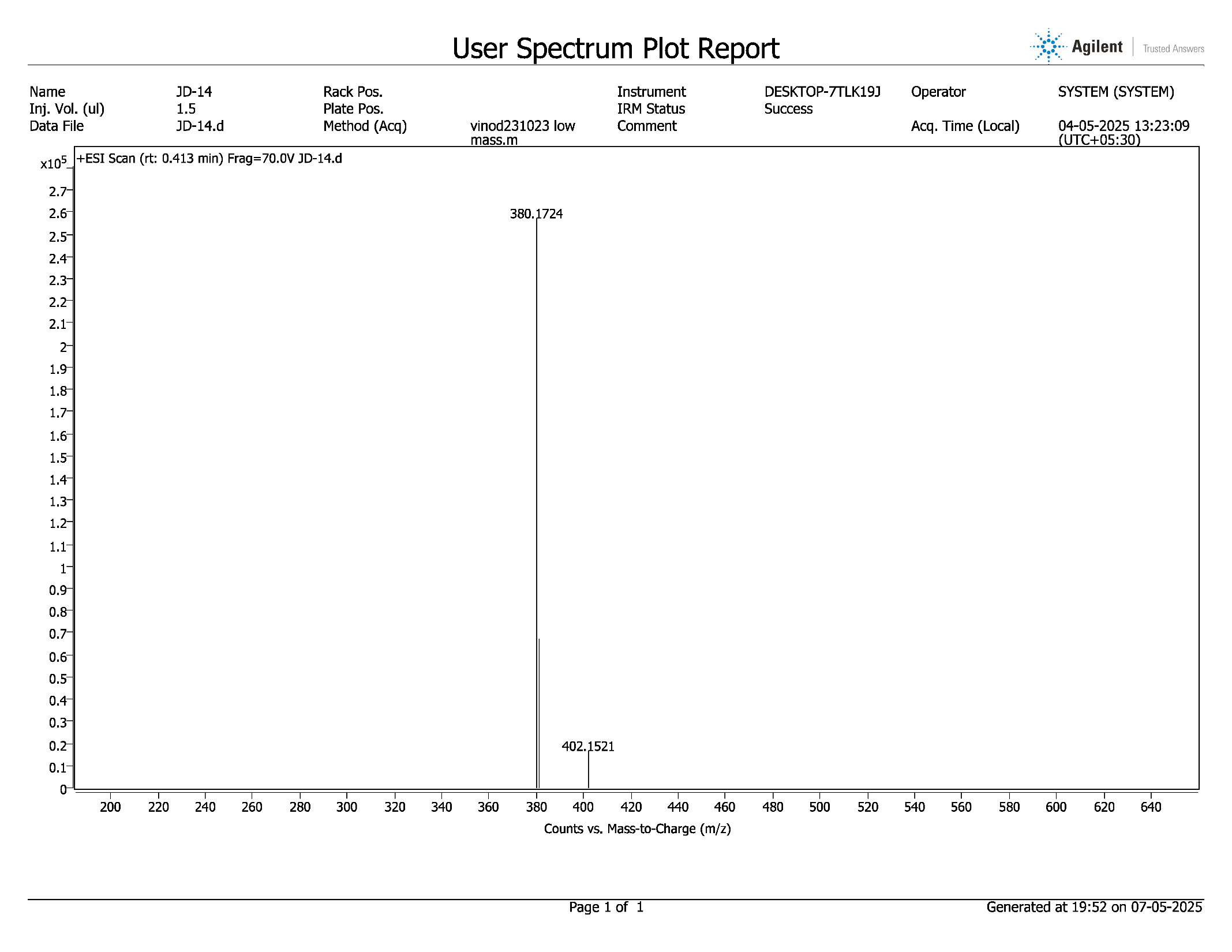


[M+H]^+^

^1^H NMR of **JRC-14**:


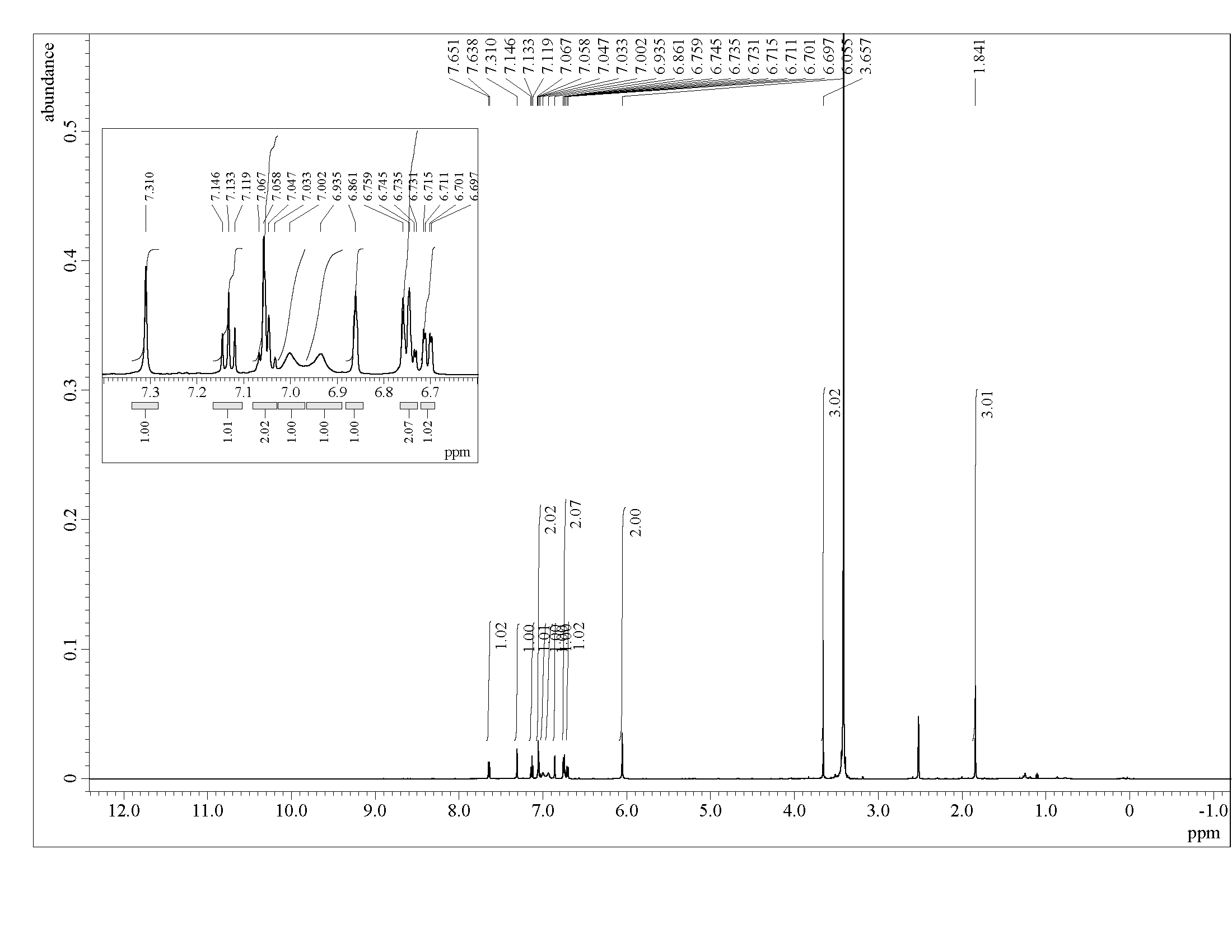


^13^C NMR of **JRC-14**:


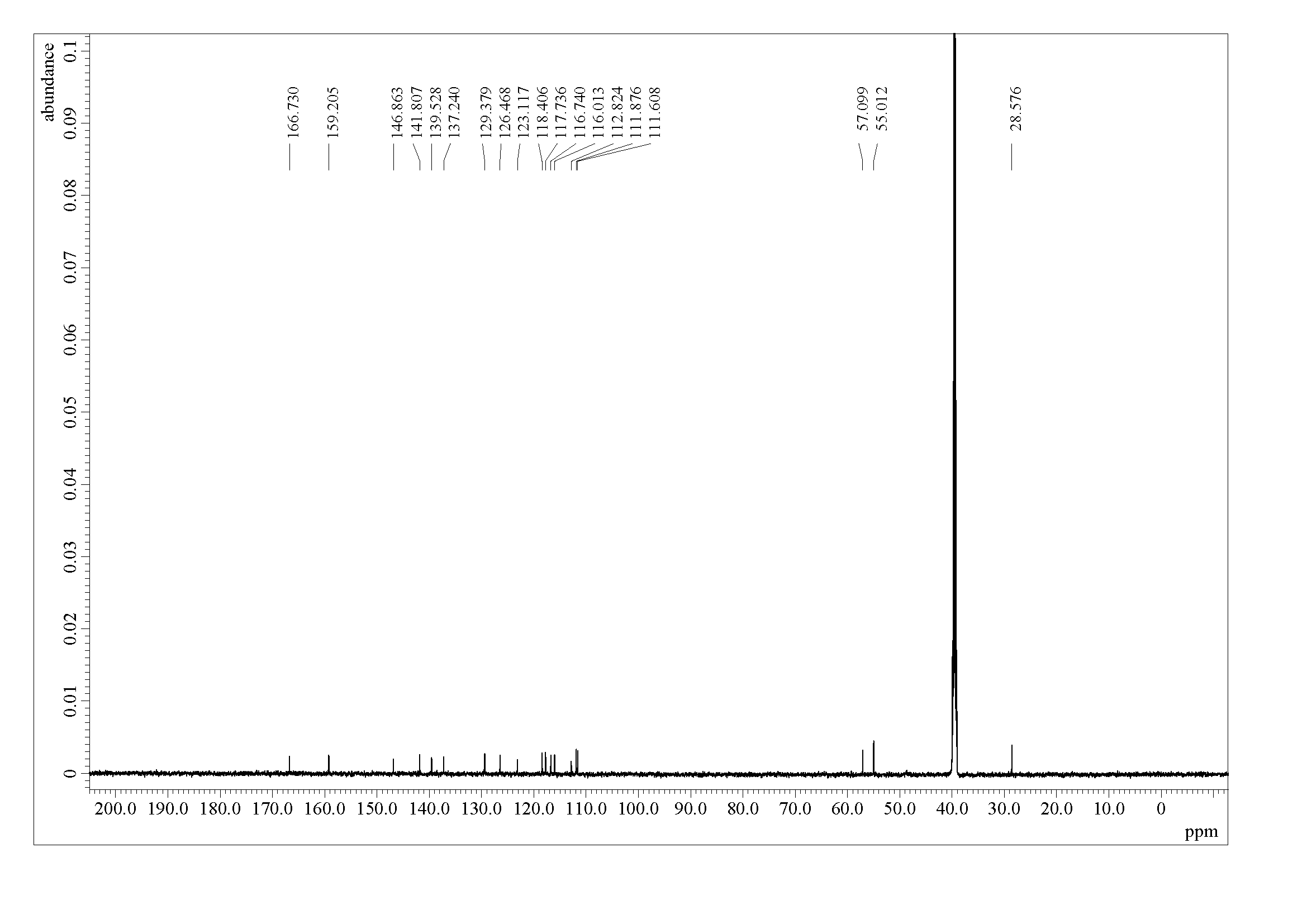


HRMS of **JRC-14**:


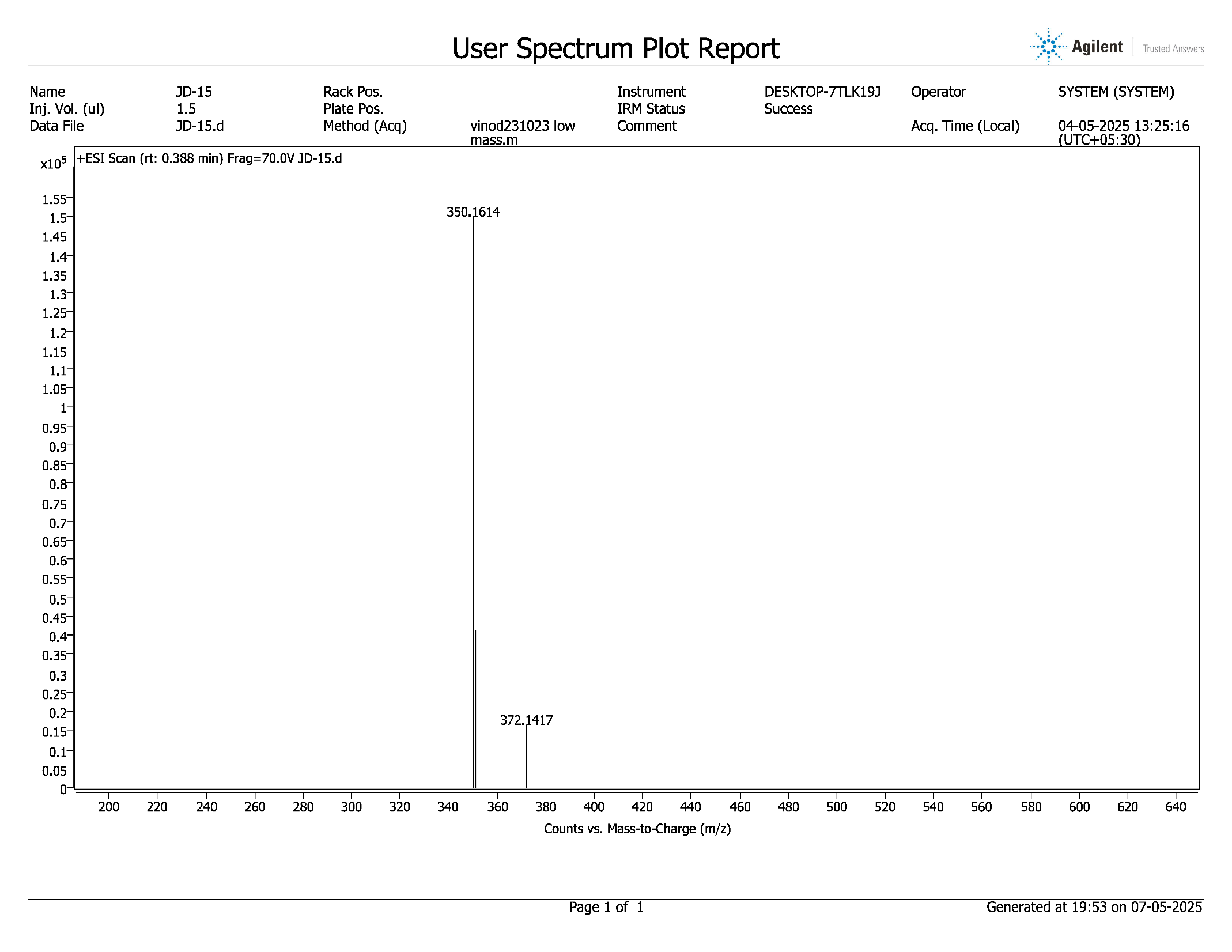


[M+H]^+^

^1^H NMR of **JRC-15**:


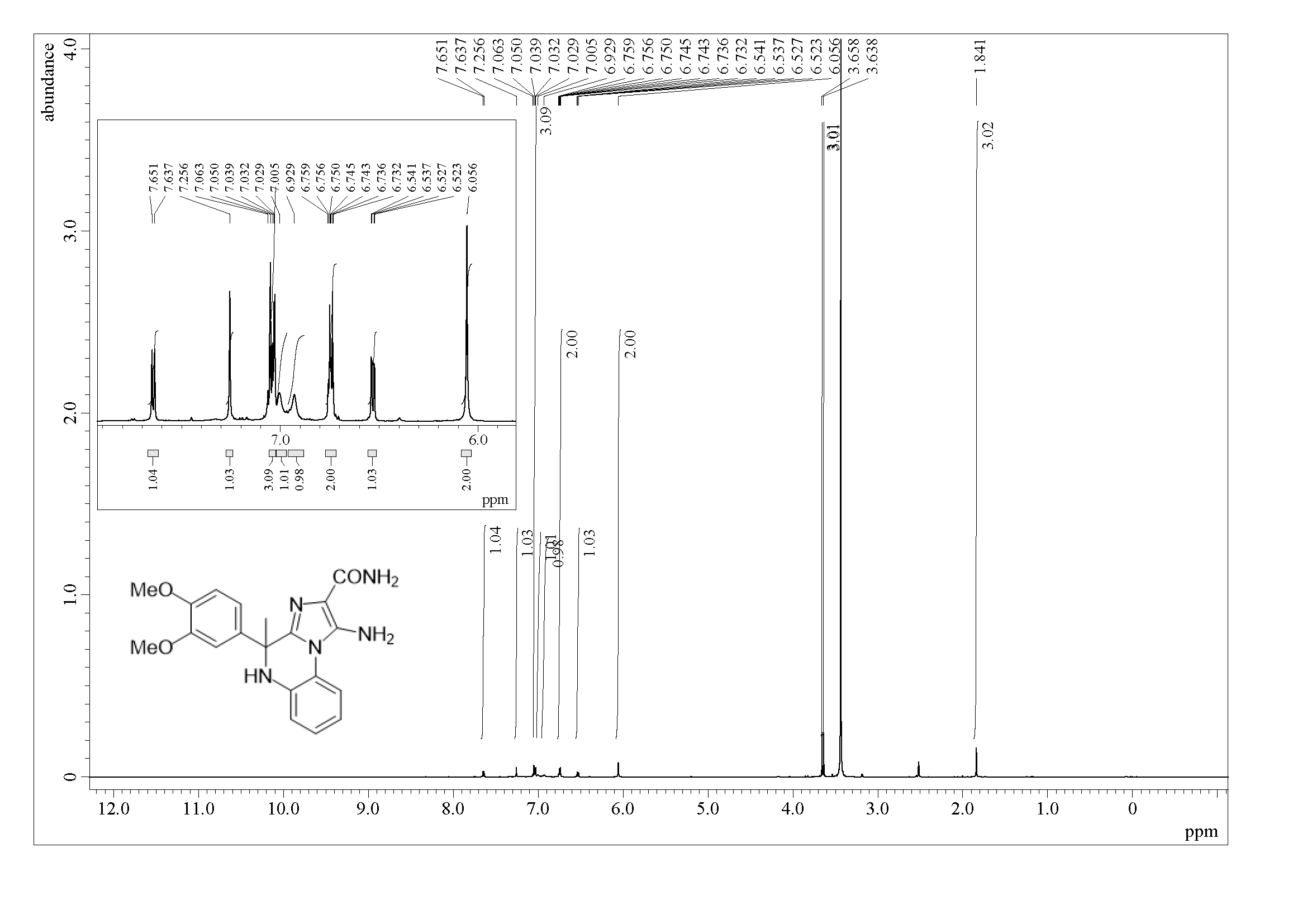


^13^C NMR of **JRC-15**:


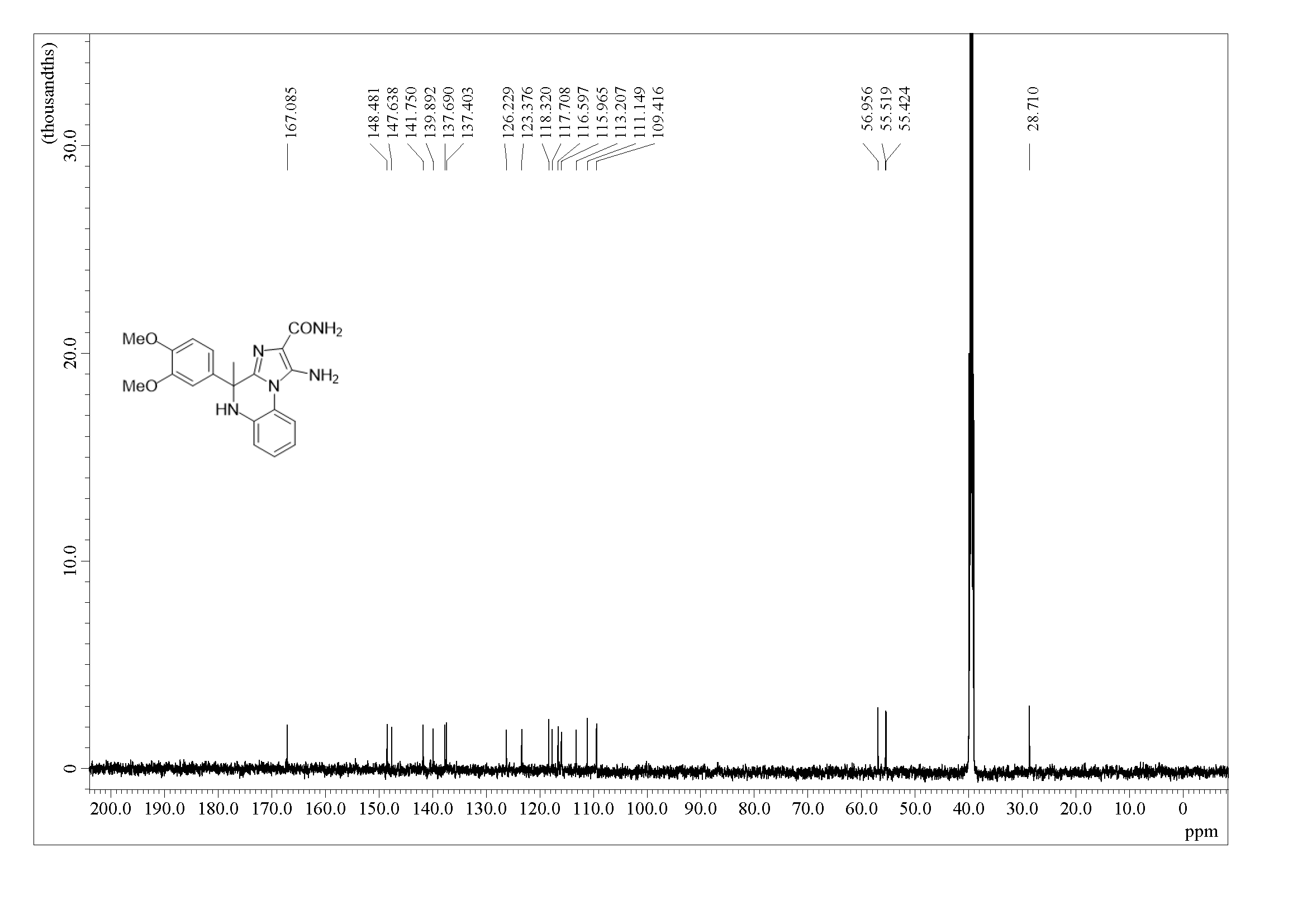


HRMS of **JRC-15**:


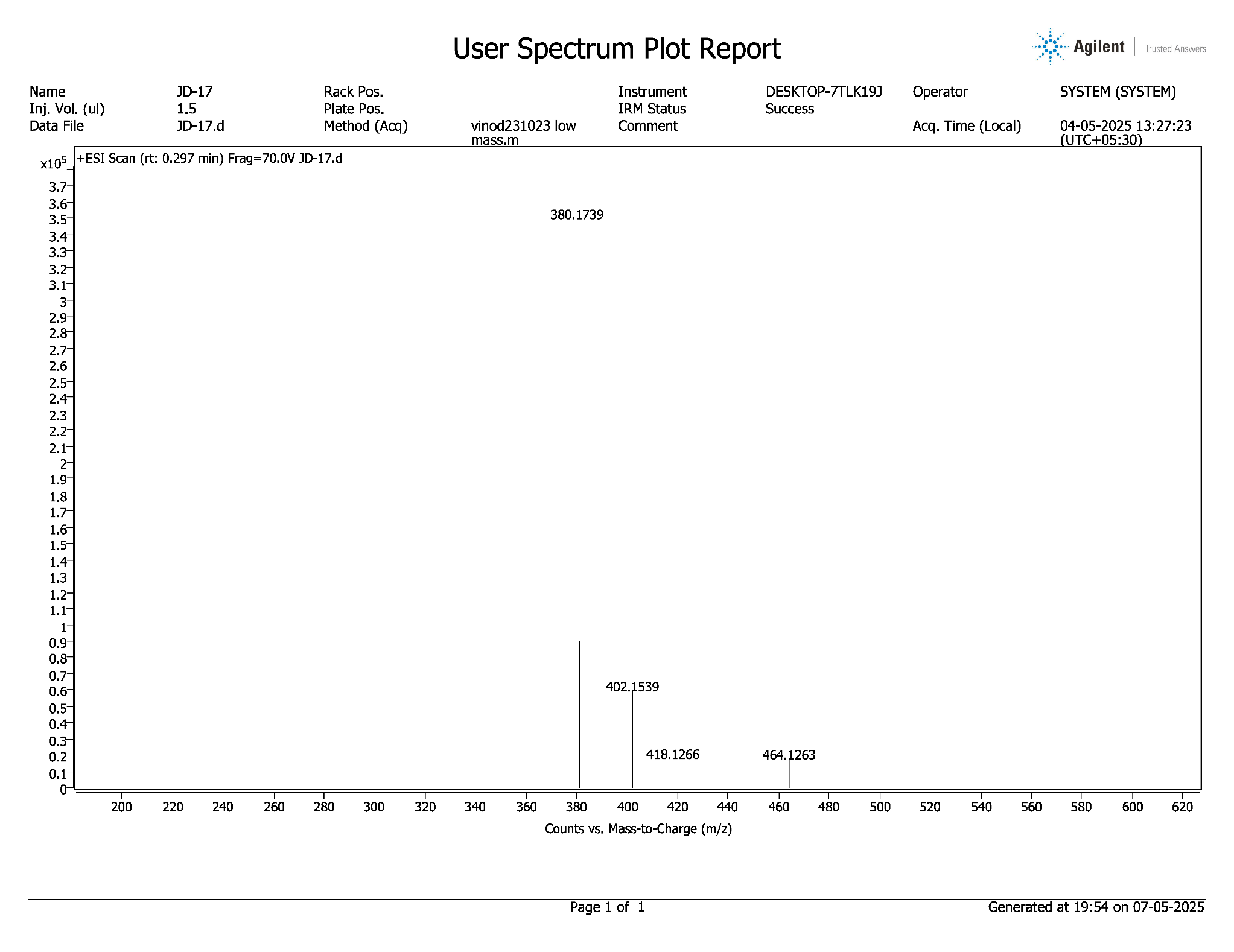


[M+H]^+^

^1^H NMR of **JRC-16**:


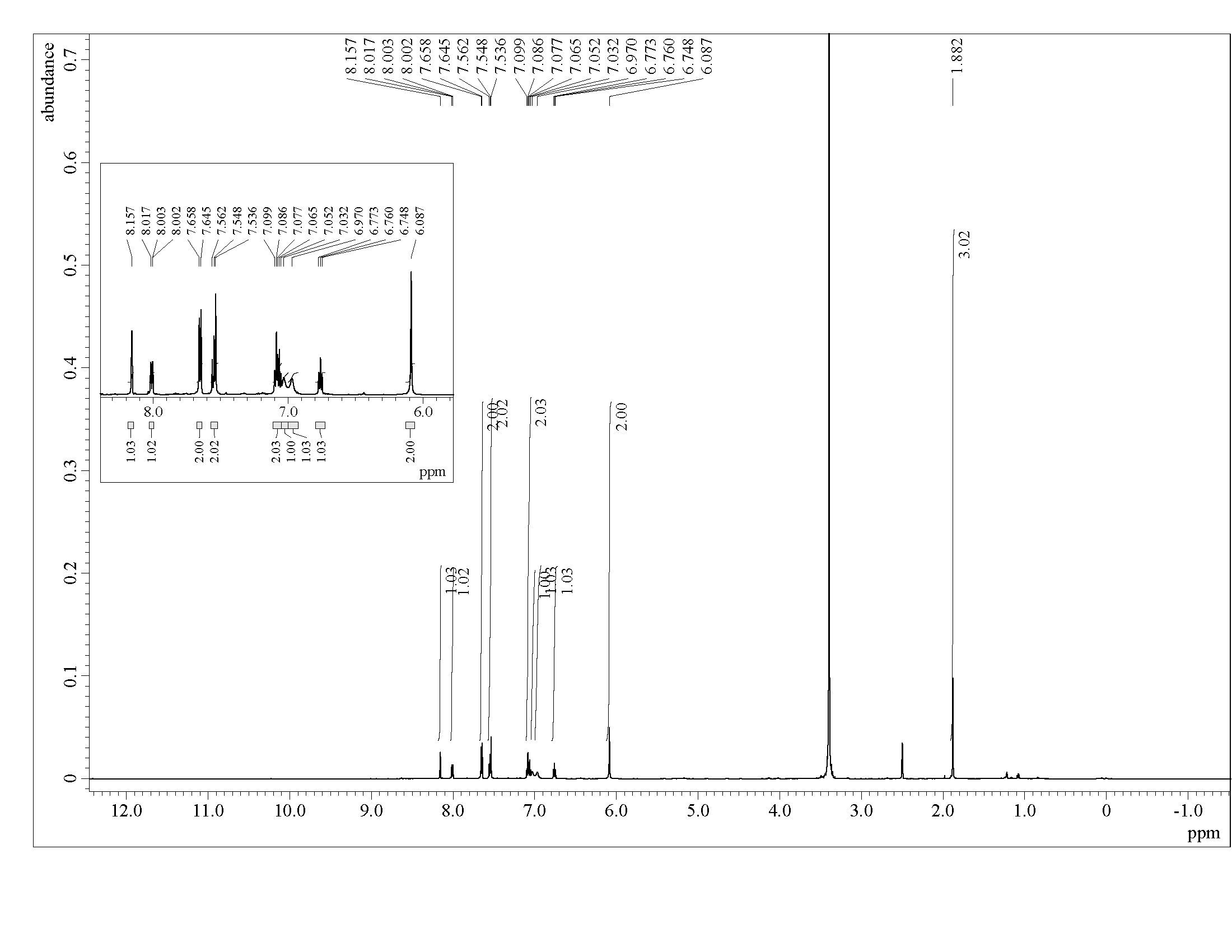


^13^C NMR of **JRC-16:**


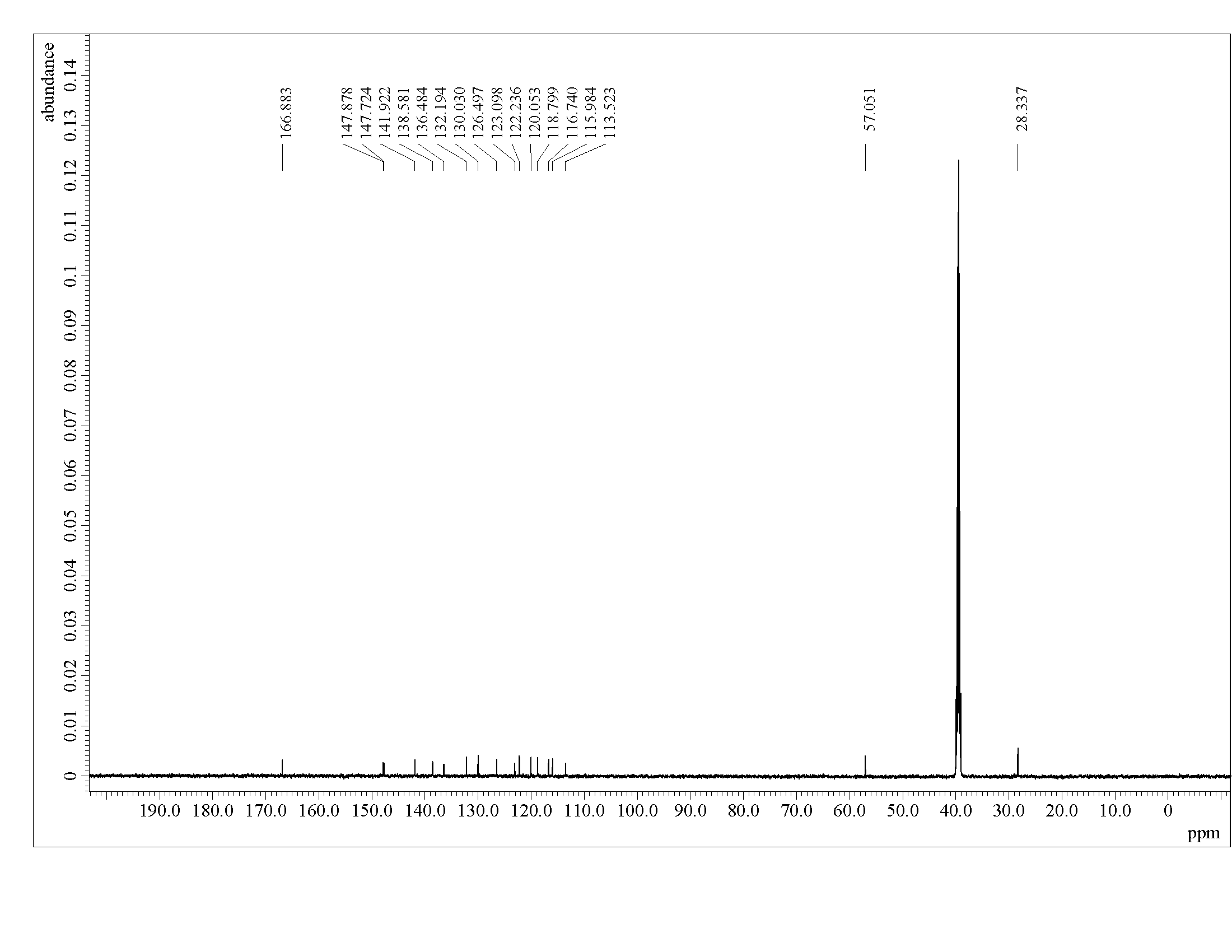


HRMS of **JRC-16**:


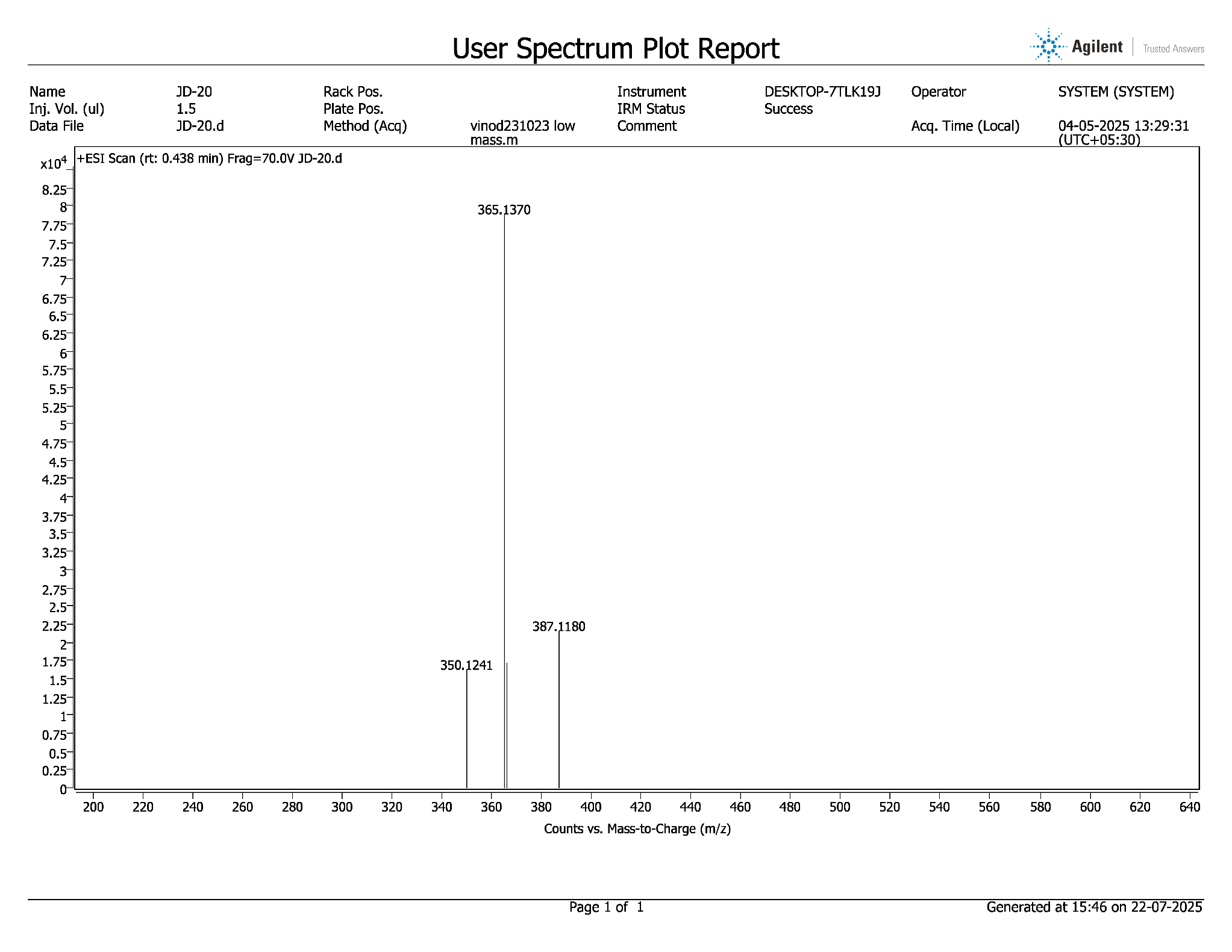


[M+H]^+^

^1^H NMR of **JRC-17**:


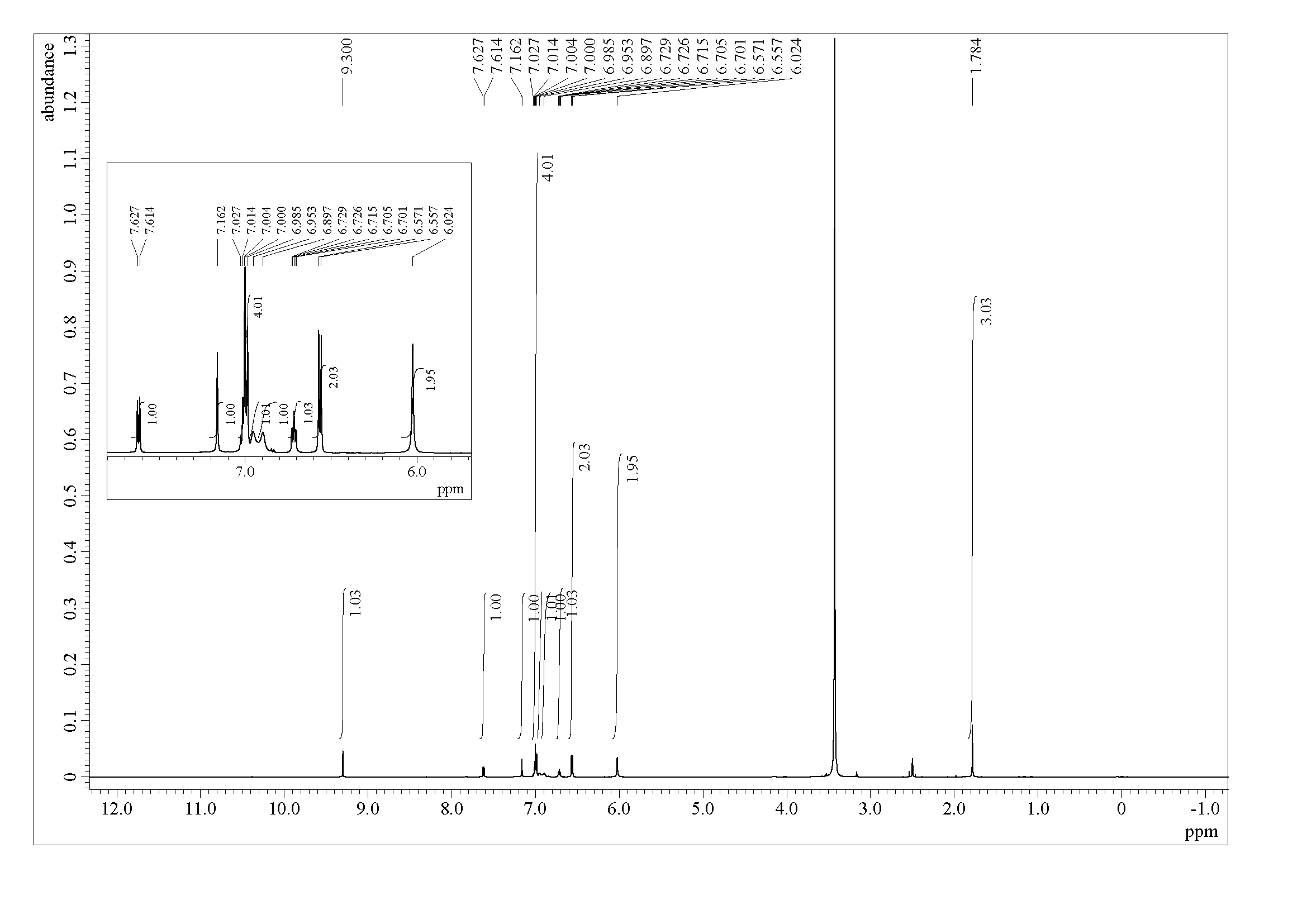


^13^C NMR of **JRC-17**:


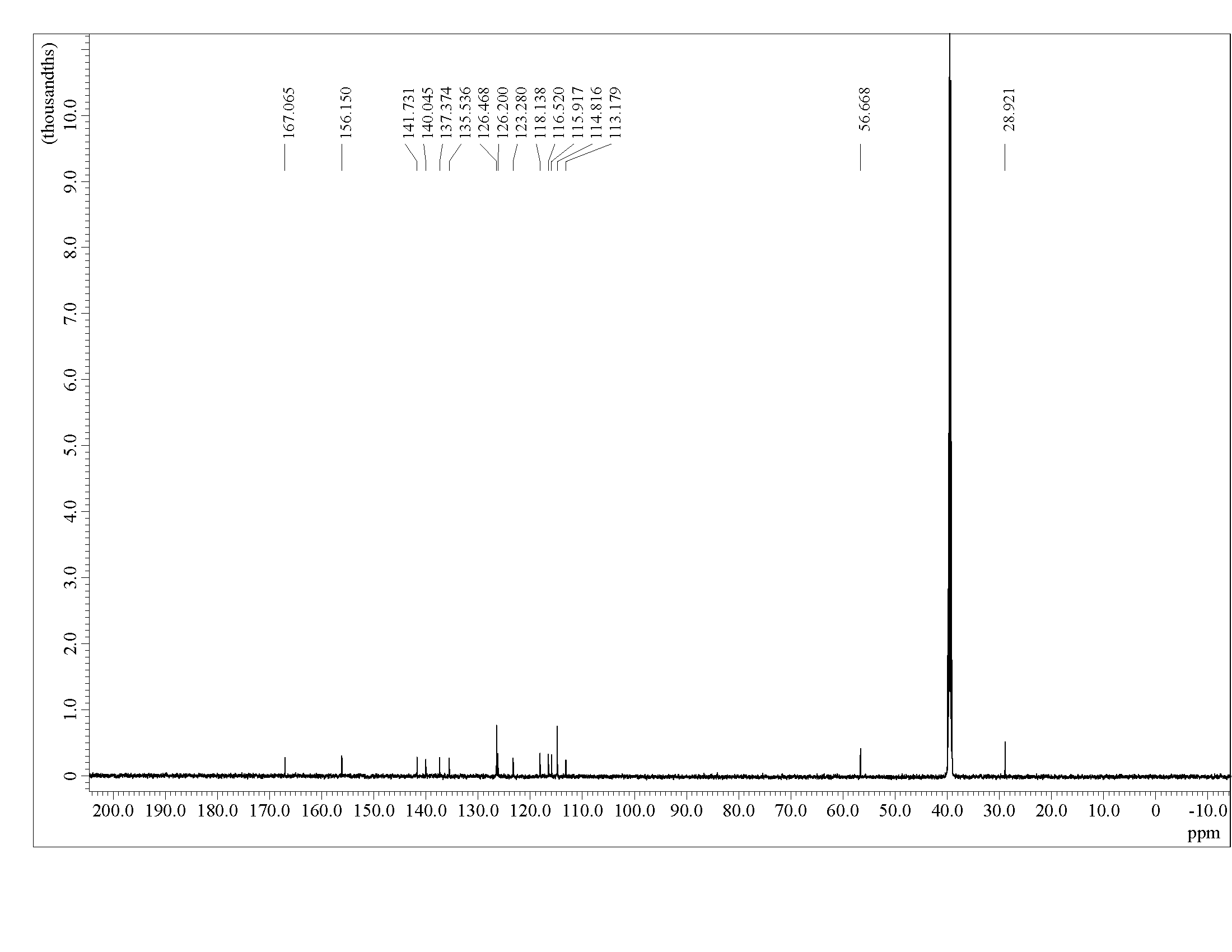


HRMS of **JRC-17**:


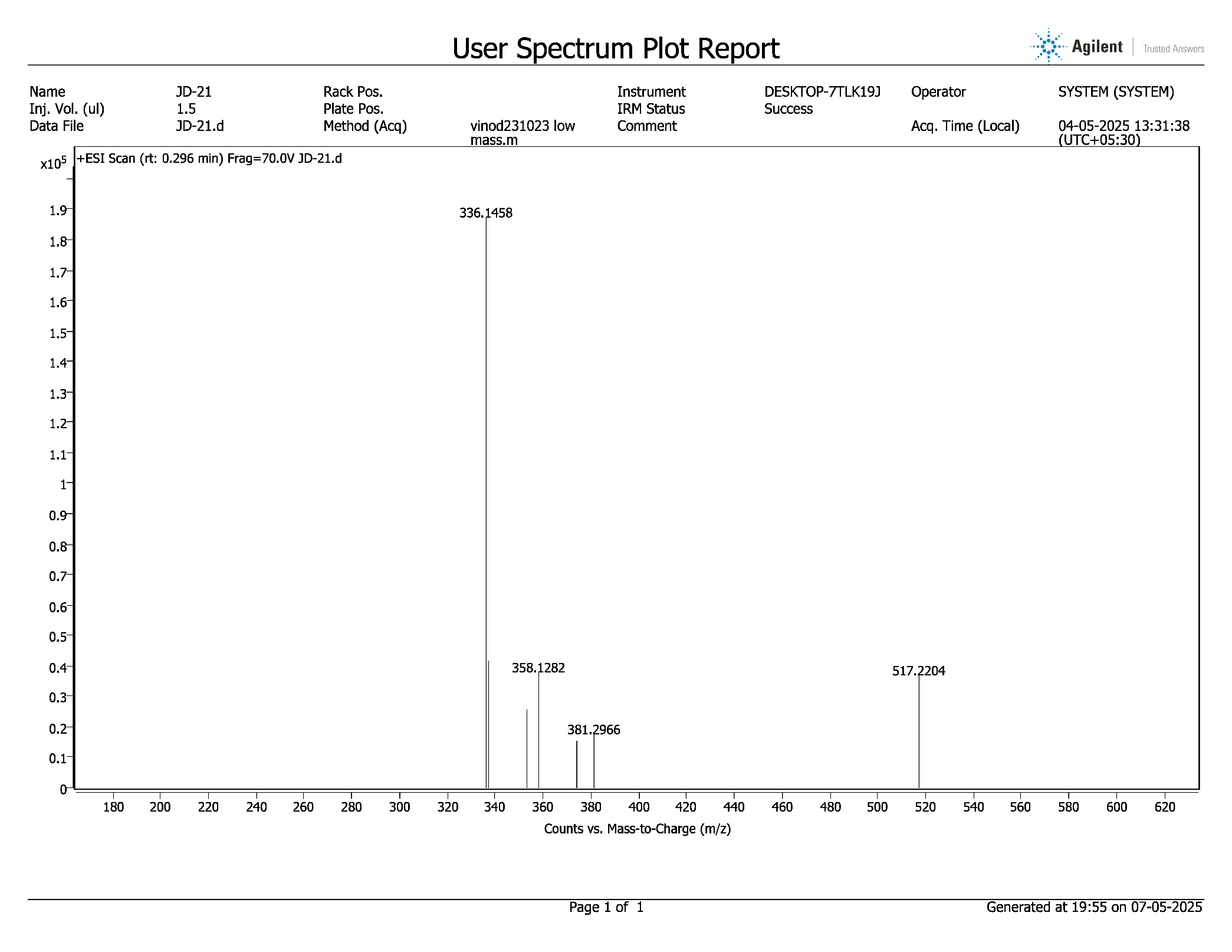


[M+H]^+^

^1^H NMR of **JRC-18**:


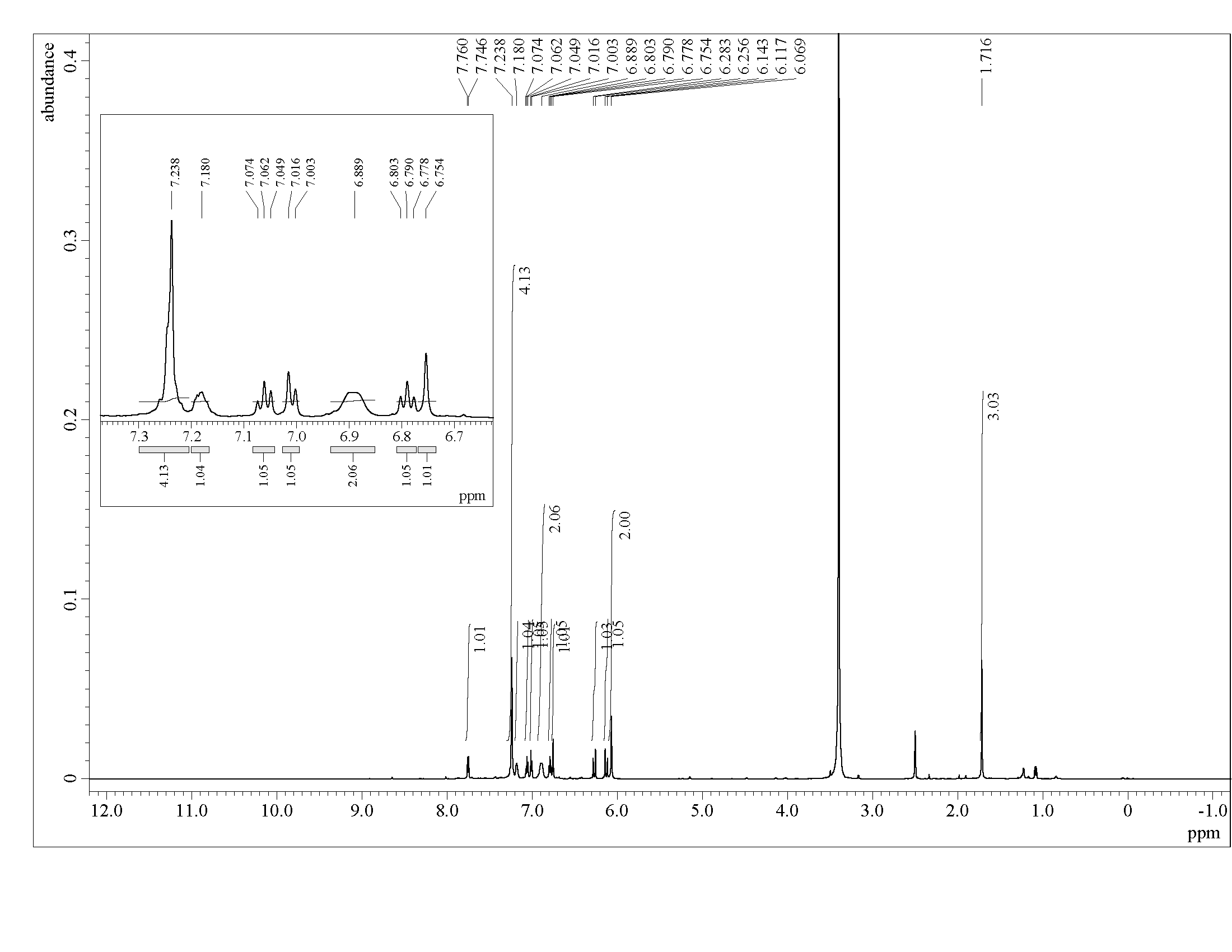


^13^C NMR of **JRC-18**:


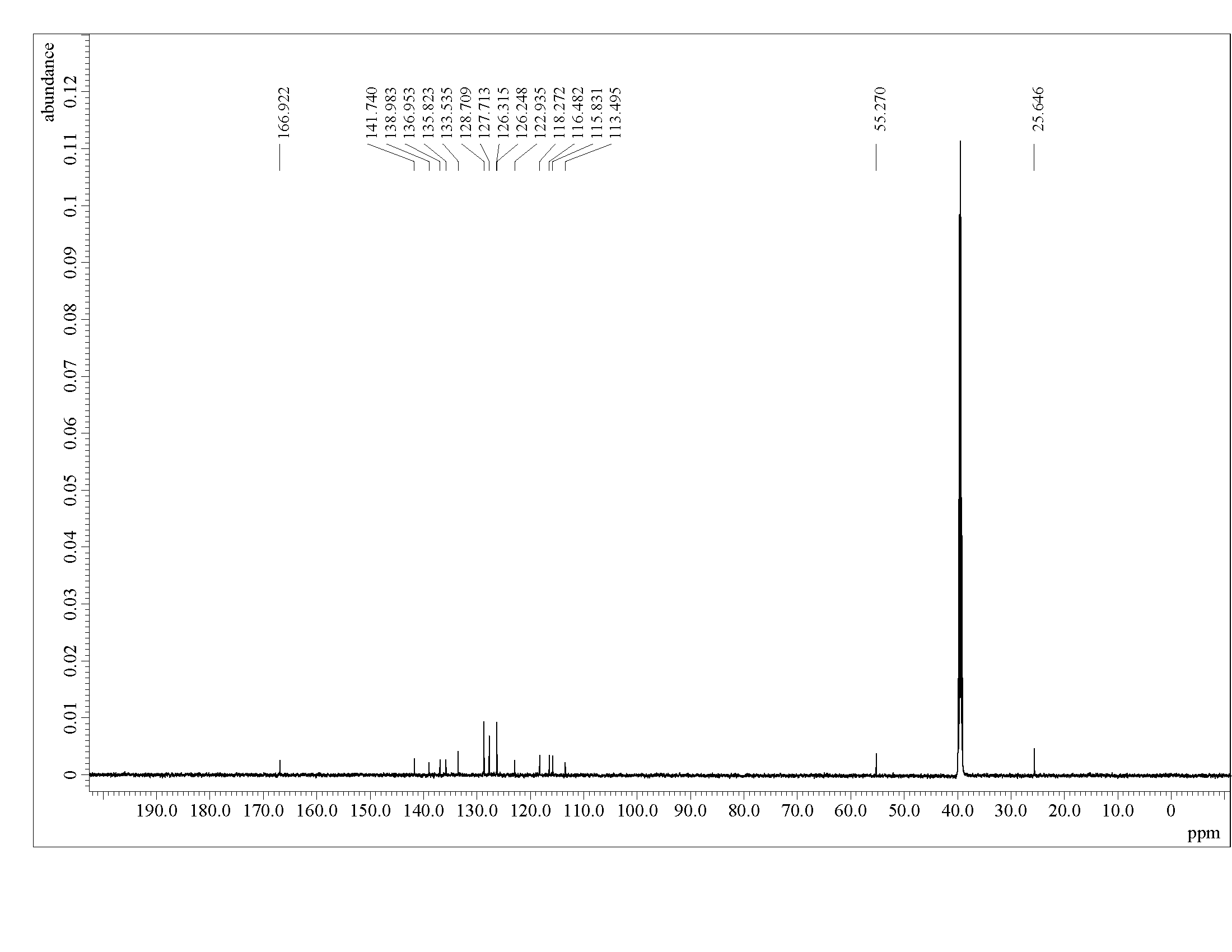


HRMS of **JRC-18**:


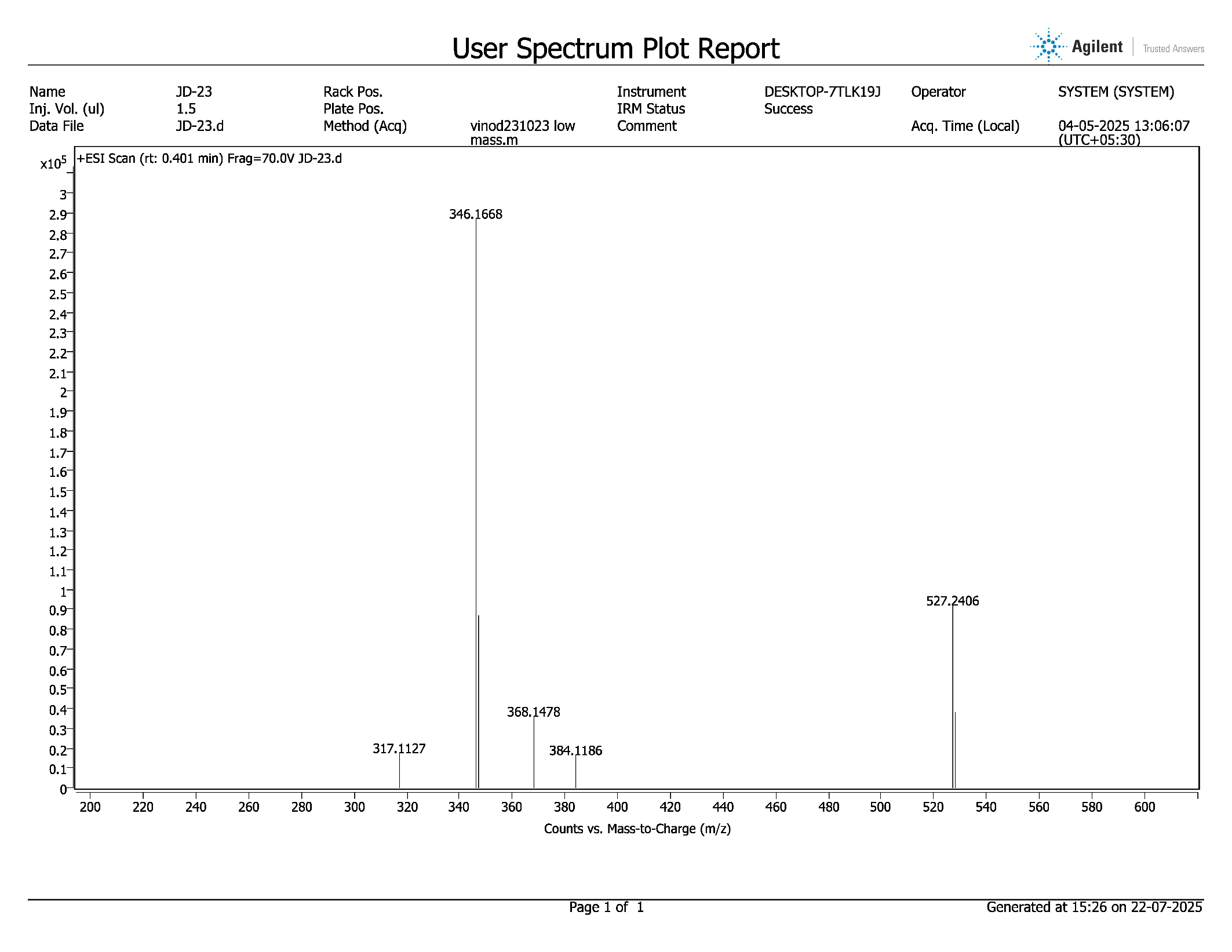


[M+H]^+^

**2. Biological study**


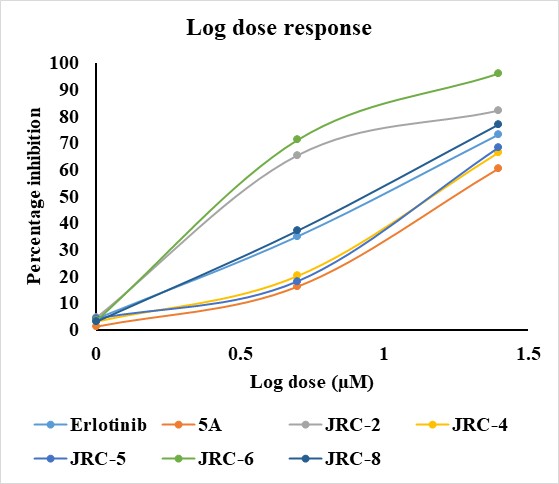

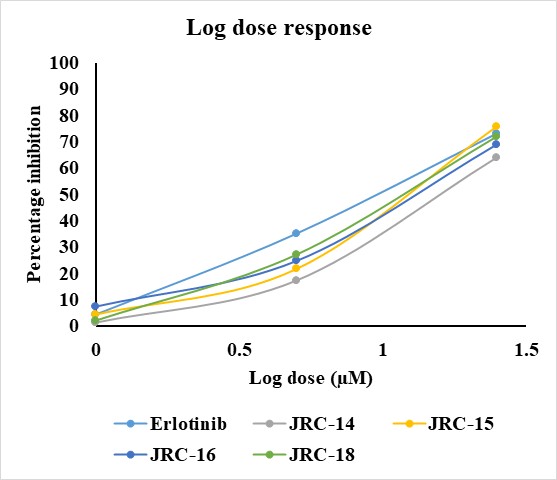


**A**

**B**

**Figure S1.** Log dose response curve of investigational compounds against MCF-7 treated at 1, 5, and 25µM for 24h


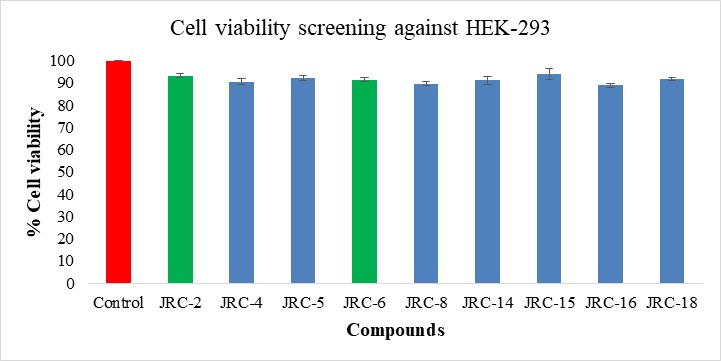


**Figure S2.** Cell viability assay of potent compounds **JRC-2, JRC-4, JRC-5, JRC-6, JRC-8, JRC-14, JRC-15, JRC-16, and JRC-18** against HEK-293 treated at 25µM for 24h

**3. Co-ordinates of optimized geometries of reactant, intermediates, and product**

**5-amino-1-(2-aminophenyl)-1*H*-imidazole-4-carboxamide (JR-A)**

**
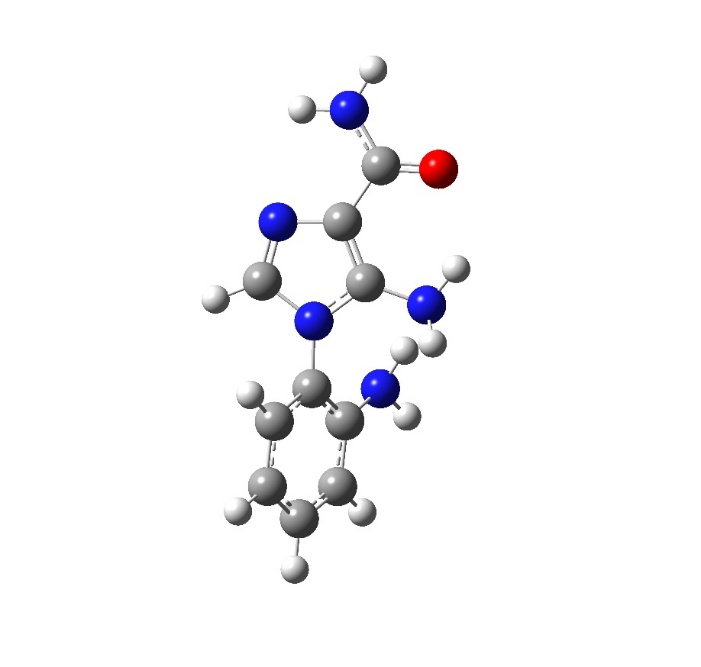
**

**Energy= -736.744647 Hartree**

0 1

C -3.53981100 -0.87314800 -0.38937400

C -4.25670900 0.11329900 0.27336000

C -3.60187900 1.20690900 0.84114200

C -2.21942300 1.29566800 0.72518100

C -1.49216600 0.31871700 0.04312000

C -2.14229200 -0.79834300 -0.52688000

H -4.06017300 -1.72085300 -0.82356100

H -5.33403300 0.02270000 0.35446400

H -4.15605500 1.97242900 1.36954700

H -1.68102800 2.12955200 1.16112200

N -0.08376400 0.50538300 -0.12961700

C 0.94391700 -0.30129000 0.31059700

C 0.52059100 1.58714000 -0.76316500

C 2.11183600 0.32216700 -0.08665900

H -0.07227900 2.37472100 -1.20150900

C 3.45778200 -0.18146800 0.18309800

O 3.63991600 -1.24671900 0.78372500

N 4.47499600 0.60167500 -0.26280800

H 4.27407800 1.45574700 -0.75923000

H 5.42430600 0.30049000 -0.11777000

N -1.44369300 -1.75021900 -1.25564900

H -0.51956100 -1.98280000 -0.91034300

H -1.97773300 -2.57280000 -1.49524000

N 1.81902900 1.49921900 -0.75498700

N 0.74757500 -1.52789800 0.91941700

H 1.64445500 -1.90517900 1.22188300

H 0.04861600 -1.53528200 1.65382900

**5-amino-1-(2-aminophenyl)-1*H*-imidazole-4-carboxamide (JR-B)**


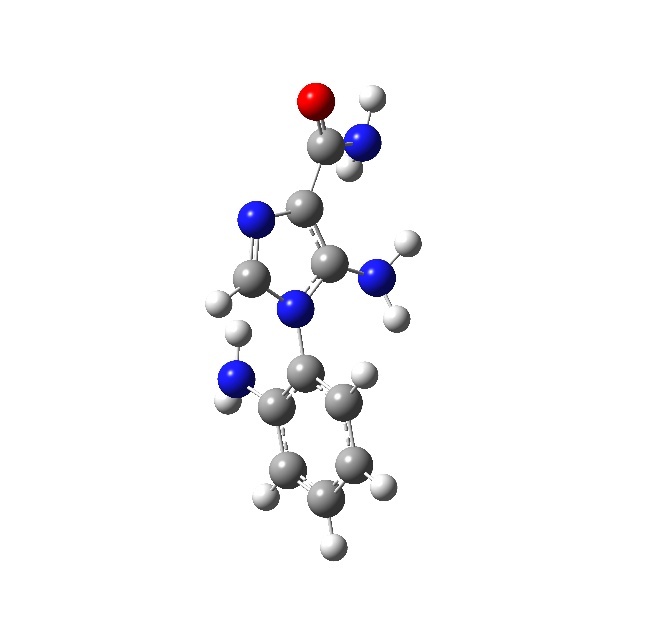


**Energy= -736.726705 Hartree**

0 1

C -3.58104100 -0.75994200 -0.42117200

C -4.17334300 0.02215800 0.56050400

C -3.42910900 0.96426800 1.27380600

C -2.07513900 1.10432600 0.98937500

C -1.46860700 0.31877300 0.00877100

C -2.21418300 -0.62839400 -0.72384400

H -4.17578700 -1.47844100 -0.97543800

H -5.23011600 -0.10221500 0.76867200

H -3.89550900 1.57813800 2.03392900

H -1.46673700 1.82753100 1.52093200

N -0.07133900 0.47372700 -0.26574600

C 0.96290600 -0.21150900 0.33798900

C 0.52536100 1.38388800 -1.13797900

C 2.13202900 0.30685600 -0.20535000

H -0.07487500 2.04535400 -1.74400400

C 3.51948400 -0.03375700 0.14802600

O 4.47201800 0.70277200 -0.01581300

N 3.66380400 -1.28198600 0.77715900

H 3.10211900 -2.04137200 0.41317200

H 4.63513100 -1.53012500 0.91609800

N -1.63412800 -1.35900500 -1.74334200

H -0.62732300 -1.41332400 -1.76206300

H -2.10149100 -2.20593700 -2.02548600

N 1.81904700 1.30625800 -1.11402900

N 0.72479200 -1.22227400 1.25590800

H 1.50756600 -1.38047400 1.87904200

H -0.15449200 -1.13836000 1.75125000

**Benzaldehyde**

**
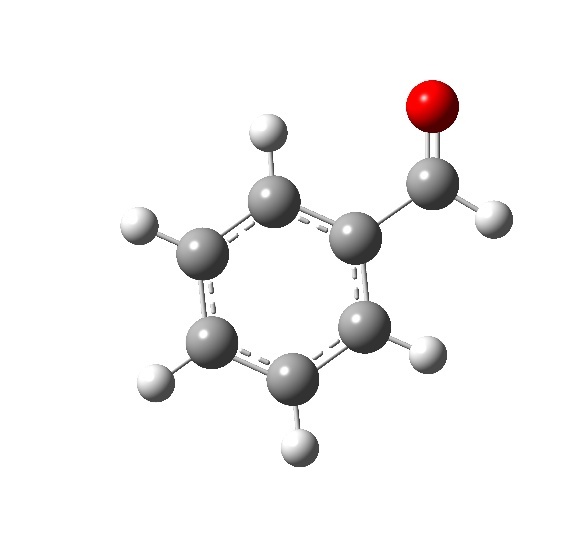
**

**Energy= -345.590310 Hartree**

0 1

C -1.99223800 0.46359200 -0.00001000

O -2.84877000 -0.39240000 0.00001000

C -0.53392200 0.20632400 -0.00000600

C 0.35484700 1.28673200 -0.00000200

C -0.03814300 -1.10472000 -0.00000500

C 1.72936400 1.06379800 0.00000300

H -0.03490400 2.30012300 -0.00000300

C 1.33268900 -1.32587300 0.00000000

H -0.74254500 -1.92824500 -0.00000800

C 2.21657900 -0.24240600 0.00000400

H 2.41740900 1.90123800 0.00000500

H 1.71926300 -2.33863700 0.00000100

H 3.28643200 -0.41910400 0.00000800

H -2.27055100 1.53913500 0.00001800

**JRC-A**

**
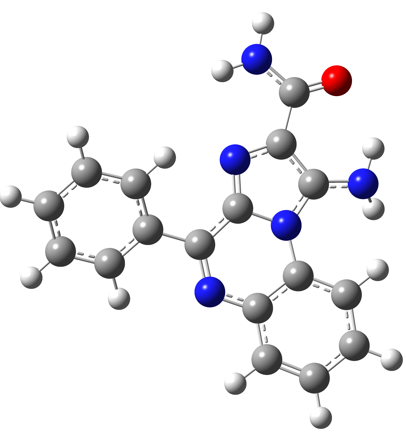
**

**Energy= -1004.703357 Hartree**

0 1

C -0.04280300 0.33175800 0.05553100

C 1.72791200 -1.36617200 0.06132200

C 0.67991600 -2.31590400 -0.02865200

C -1.02418800 -0.72415200 0.01363700

H 3.84635100 -1.06380700 0.35384100

C 3.05721600 -1.78454800 0.20046000

C 1.01781400 -3.68232600 -0.03796800

C 2.33475500 -4.09254900 0.06461400

C 3.35297400 -3.14109600 0.19781100

H 0.20494900 -4.39425600 -0.11264500

H 2.57654700 -5.14880500 0.05897200

H 4.38303700 -3.45840700 0.30953400

N -0.65547300 -1.97623300 -0.06259700

C -2.48040100 -0.43309600 0.01849600

C -3.35388600 -1.36602100 -0.56272700

C -3.01958800 0.71670600 0.61377100

C -4.72648700 -1.15043100 -0.55790200

H -2.93714700 -2.25745400 -1.01315300

C -4.39727000 0.92173400 0.62785900

H -2.36183500 1.44421200 1.06660000

C -5.25467500 -0.00538700 0.03980100

H -5.38641200 -1.87637400 -1.01962100

H -4.80061000 1.80969500 1.10179100

H -6.32614500 0.16111700 0.04725200

N 1.32459100 -0.02236600 0.02918900

C 2.03167700 1.15623700 -0.04278500

C 1.07059100 2.16310200 -0.01959500

C 1.36935300 3.59956100 -0.10998000

N 0.28639900 4.41680400 -0.13381600

H -0.64265100 4.02581400 -0.13132200

H 0.42603800 5.40961300 -0.22598900

O 2.53104500 4.01238800 -0.16467700

N 3.40303600 1.27595500 -0.07244100

H 3.87913500 0.68459900 -0.74210400

H 3.65290700 2.25939800 -0.17092400

N -0.18997800 1.64027700 0.03866700

**JRC-B**

**
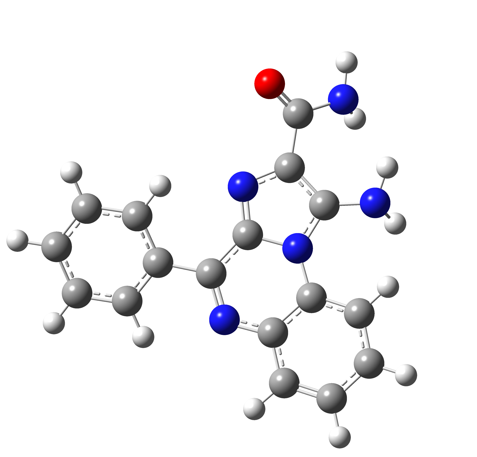
**

**Energy= -1004.683438 Hartree**

0 1

C 1.11537500 -0.61600400 0.00408000

C -0.43567300 -2.36116300 0.01571900

C -1.57115200 -1.51632600 -0.06068900

C 0.03174300 0.34074500 -0.04474300

H 0.24008900 -4.38403200 0.07173600

C -0.63902400 -3.75464300 0.00750500

C -2.85447000 -2.06231700 -0.19636300

C -3.01611900 -3.44094700 -0.21088300

C -1.90926900 -4.29065700 -0.09641100

H -3.71055400 -1.42026400 -0.33505600

H -4.01115200 -3.85568800 -0.32158900

H -2.04660400 -5.36549700 -0.10477300

C 2.54039500 -0.19520800 0.01529000

C 3.50992300 -1.13351300 0.41083000

C 2.96265200 1.08557200 -0.37578900

C 4.85795000 -0.80103000 0.41947100

H 3.18482100 -2.12170400 0.70773600

C 4.31789400 1.40922300 -0.37318400

H 2.23379800 1.82525500 -0.67246600

C 5.26899900 0.47329700 0.02441200

H 5.59069800 -1.53580600 0.73421900

H 4.62703600 2.40110500 -0.68308800

H 6.32203000 0.73253400 0.02869900

N 0.85936900 -1.89701300 0.06025300

N -1.29359000 -0.14034700 -0.01574800

C -1.24189800 2.06504900 0.02191400

C -2.10507100 0.97132700 0.05318800

N 0.05636900 1.65464200 -0.05002700

N -3.49462700 0.91147000 0.03335100

H -3.90868300 0.35896400 0.77550700

H -3.90369800 1.83801200 0.00124000

C -1.59478000 3.50795300 -0.00923300

N -2.77162700 3.83571000 0.66928900

H -2.94533400 3.35134600 1.54016100

H -2.93633000 4.83351800 0.71492200

O -0.95652700 4.33785900 -0.62097000

**5-amino-1-(2-aminophenyl)-1*H*-imidazole-4-carbonitrile (4)**


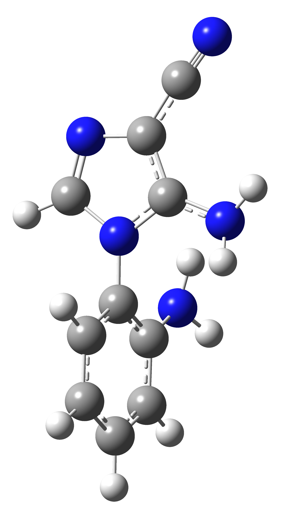


**Energy= -660.265528 Hartree**

0 1

C -3.17746300 0.92150000 0.16328300

C -3.83433900 -0.15539500 -0.41484700

C -3.14240900 -1.31975800 -0.75531700

C -1.77554700 -1.38291700 -0.50999700

C -1.10697400 -0.30509100 0.07097400

C -1.79657200 0.87312800 0.42585100

H -3.73150000 1.81503600 0.43112700

H -4.90020100 -0.08589400 -0.60072400

H -3.65783600 -2.15973100 -1.20336900

H -1.20547900 -2.26999500 -0.76247900

N 0.30006900 -0.40002800 0.32870600

C 1.31120300 0.11346100 -0.45226300

C 0.91939100 -1.04899300 1.39429700

C 2.49067600 -0.24247700 0.18496300

H 0.33781500 -1.54712000 2.15439700

N -1.15454600 1.91673100 1.06468100

H -0.15216700 1.97631000 0.96952600

H -1.61733900 2.81207500 1.04654800

N 2.21396200 -0.96744900 1.33679300

N 1.06458400 0.88986700 -1.56492300

H 1.87951100 1.05685900 -2.13965600

H 0.25093700 0.62926200 -2.10744300

C 3.78955500 0.07266500 -0.27289800

N 4.82323500 0.36323100 -0.70700100

**3,4,5-trimethoxy benzaldehyde**

**
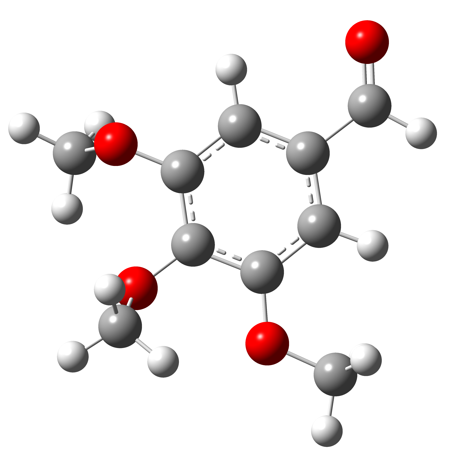
**

**Energy= -689.161719 Hartree**

0 1

C -3.34043800 -0.30400100 -0.13247100

O -4.14343700 0.59467600 -0.25367000

C -1.87297900 -0.13544500 -0.07544700

C -1.06971300 -1.27438000 0.07076300

C -1.29827500 1.13265700 -0.16001100

C 0.31624600 -1.14796900 0.12505600

H -1.54220400 -2.24585700 0.14500300

C 0.08481100 1.26642300 -0.10962400

H -1.92314700 2.00889500 -0.27778200

C 0.90465600 0.13104200 0.02013800

H -3.68444200 -1.35811300 -0.05287100

O 0.63987100 2.50912600 -0.26196600

O 2.25639400 0.29636700 0.10389600

O 1.18565500 -2.18658200 0.27237700

C 0.65536700 -3.49833700 0.42735400

H 0.08842400 -3.80732500 -0.45719100

H 1.51678200 -4.15215700 0.55099000

H 0.01793400 -3.56623700 1.31488400

C 3.02278900 -0.15081100 -1.02688100

H 2.91047800 -1.22649100 -1.17340400

H 2.71773800 0.39022000 -1.92749500

H 4.06070000 0.08293500 -0.79408200

C 1.27154000 3.06419700 0.90141000

H 2.09699400 2.43471400 1.23893600

H 1.64885000 4.04040900 0.59942400

H 0.54000200 3.19004500 1.70676100

**JRC-2C**


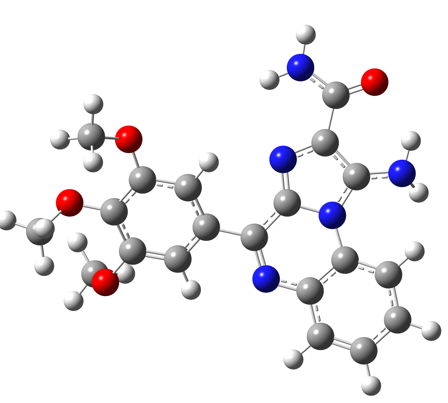


**Energy= -1348.271162 Hartree**

0 1

C -1.39490400 0.31760900 -0.00906100

C -3.13427500 -1.40040800 -0.22651600

C -2.06880000 -2.33470900 -0.24026400

C -0.39295100 -0.71859300 -0.07966800

H -5.25640900 -1.10432500 -0.49750000

C -4.45466800 -1.82369100 -0.42386200

C -2.38095500 -3.69894300 -0.39383800

C -3.68922500 -4.11809900 -0.55423600

C -4.72471600 -3.17631300 -0.58275400

H -1.55522900 -4.39992100 -0.39644600

H -3.91087800 -5.17197400 -0.67443200

H -5.74795500 -3.49699600 -0.73883900

N -0.74152400 -1.97708000 -0.15498500

C 1.05779400 -0.40986600 -0.03540400

C 1.94676300 -1.42831300 0.34003100

C 1.57587600 0.84069300 -0.38262400

C 3.30962900 -1.18867400 0.37938700

H 1.57163300 -2.40581000 0.61065500

C 2.95032800 1.08100600 -0.37497800

H 0.92543100 1.65193000 -0.67108700

C 3.84063900 0.06133900 0.00381100

N -2.75580600 -0.06286200 -0.03486700

C -3.48644400 1.08699800 0.16180000

C -2.54577100 2.10812200 0.25876200

N -4.86047900 1.17675100 0.19264800

H -5.32800000 0.50753400 0.79139100

H -5.12905600 2.13868400 0.39753800

N -1.27537800 1.61859900 0.15380900

O 3.33925600 2.35914600 -0.67605500

O 5.18312100 0.32426700 0.09906800

O 4.16449000 -2.19532100 0.78191100

C 4.41905500 2.56657800 -1.59123100

H 5.38560300 2.41694500 -1.10964300

H 4.32644300 1.90224300 -2.45712000

H 4.32852400 3.60052300 -1.92441100

C 6.04941800 -0.43201300 -0.76489900

H 5.93455800 -1.50328400 -0.59843400

H 5.84097500 -0.19461800 -1.81345700

H 7.06374500 -0.12063500 -0.51702500

C 4.54989900 -2.12562200 2.16133700

H 3.67250500 -2.21076700 2.81086500

H 5.21492900 -2.97100200 2.33675600

H 5.07594900 -1.19048400 2.37537200

C -2.87187500 3.52098400 0.50148100

N -1.80526100 4.35064600 0.61869000

H -1.96450600 5.32722800 0.80453200

H -0.86764300 3.98407000 0.56477500

O -4.04191500 3.90312800 0.59455000

**JRC-2D**


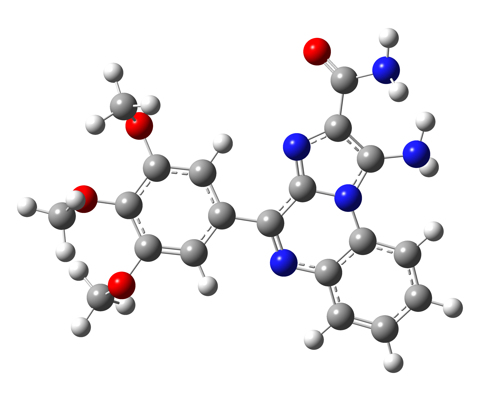


**Energy= -1348.252172 Hartree**

0 1

C -1.36064900 0.28228500 -0.00379800

C -3.08063000 -1.47066200 -0.00407800

C -2.00007100 -2.38514800 0.07262900

C -0.34228900 -0.74102000 0.06979500

H -5.21219200 -1.24642300 -0.28708500

C -4.39517100 -1.93638000 -0.14043600

C -2.29149000 -3.76278800 0.07210600

C -3.59300000 -4.21887900 -0.02931400

C -4.64404700 -3.30236900 -0.14882100

H -1.45392300 -4.44592100 0.14214400

H -3.79736900 -5.28303100 -0.03115900

H -5.66324000 -3.65364600 -0.25882100

N -0.67846400 -2.00391400 0.12355100

C 1.10348500 -0.41035100 0.08322400

C 2.02015200 -1.43028500 -0.21400200

C 1.59454400 0.85974300 0.41368100

C 3.38371300 -1.18551600 -0.19831500

H 1.66719000 -2.42534700 -0.44805000

C 2.96368000 1.11089400 0.42693600

H 0.92566700 1.67140100 0.65932500

C 3.88042800 0.09933000 0.09231000

N -2.71611900 -0.11412100 0.02148300

C -3.45450600 1.04708200 0.02229500

C -2.52420700 2.08415500 -0.05281900

N -4.84212400 1.07994600 0.09495800

H -5.22926100 0.47607900 0.81122700

H -5.17355800 2.03112500 0.21877600

N -1.25257600 1.58941200 -0.05720200

O 3.39696000 2.34464000 0.84480000

O 5.21998600 0.37185000 0.14150700

O 4.25732600 -2.21462900 -0.49023300

C 3.88093400 3.22256500 -0.17299600

H 4.78875300 2.83018100 -0.63943300

H 3.11259800 3.39184000 -0.93536100

H 4.11071600 4.16494900 0.32333700

C 5.96269000 0.19047100 -1.07542900

H 5.93393400 -0.84701700 -1.41014500

H 5.56616800 0.84032800 -1.86284900

H 6.98579400 0.48458500 -0.84383400

C 4.85647700 -2.82199500 0.66266100

H 4.08887900 -3.26332400 1.30684600

H 5.51266300 -3.60770900 0.28856600

H 5.43967300 -2.09351200 1.23374900

C -2.79509400 3.54059400 -0.04137800

N -4.03893900 3.89539100 -0.57617600

H -4.17046400 4.89750900 -0.63489500

H -4.33733800 3.39721900 -1.40539200

O -2.04762800 4.36355900 0.44408400

**
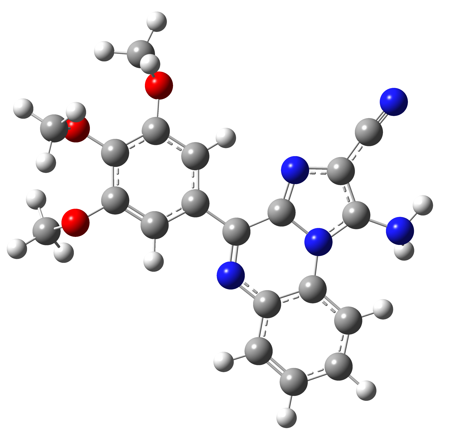
**

**1-amino-4-(3,4,5-trimethoxyphenyl)imidazo[1,2-a]quinoxaline-2-carbonitrile**

**Energy= -1271.789942 Hartree**

0 1

C -1.49207400 0.62122900 -0.02357700

C -3.34305400 -0.99553000 -0.03885500

C -2.33657400 -1.99022800 0.02159000

C -0.55263700 -0.47970800 0.03474700

H -5.45248100 -0.59672800 -0.27967100

C -4.69206300 -1.35276800 -0.15840200

C -2.73357500 -3.34145600 0.00908700

C -4.06762200 -3.69213400 -0.08308900

C -5.04586200 -2.69478900 -0.17757200

H -1.95170200 -4.08881000 0.06437400

H -4.35512300 -4.73678100 -0.09422700

H -6.09064400 -2.96490000 -0.27524900

N -0.99082100 -1.71092200 0.07132100

C 0.91661000 -0.27228800 0.05138300

C 1.74451600 -1.38661300 -0.16307500

C 1.51282300 0.97037300 0.29088100

C 3.12131300 -1.25361900 -0.13859000

H 1.31085700 -2.36130700 -0.33768600

C 2.90102000 1.11331700 0.30758300

H 0.91653900 1.85416000 0.45802000

C 3.72762000 -0.00000400 0.07788600

N -2.87355100 0.32832300 -0.00165900

C -3.52702400 1.53720800 0.01855300

C -2.52056900 2.49688000 -0.03020100

N -4.90305200 1.69634600 0.02002200

H -5.38278500 1.23274600 0.78355000

H -5.16780900 2.67383900 -0.01836100

N -1.28257500 1.91889600 -0.06126700

C -2.74973200 3.89513900 -0.03095700

N -3.01251300 5.02184900 -0.02484100

O 3.37611000 2.35504800 0.62939900

O 5.08897300 0.12894200 0.16426700

O 3.91304500 -2.36612300 -0.34220200

C 4.39278900 2.94792200 -0.18417800

H 5.37733400 2.53968400 0.04599600

H 4.16841100 2.80829200 -1.24710900

H 4.37117400 4.01270800 0.04722100

C 5.83953400 -0.15482600 -1.02981600

H 5.65216200 -1.16965800 -1.38150000

H 5.58619500 0.56380300 -1.81621400

H 6.88807400 -0.03976900 -0.75703000

C 4.39839700 -2.97332200 0.86315900

H 3.56403600 -3.31647600 1.48362800

H 4.99801000 -3.82946100 0.55464900

H 5.01730500 -2.27441900 1.43350800

**Water**


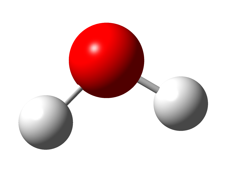


**Energy= -76.454816 Hartree**

0 1

O 0.00000000 -0.00000000 0.11702500

H -0.00000000 0.76350200 -0.46810000

H -0.00000000 -0.76350200 -0.46810000

H -0.00000000 -0.76350200 -0.46810000

**H_2_**

**
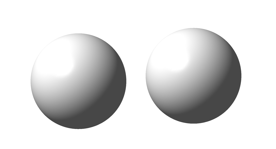
**

**Energy= -1.180993Hartree**

0 1

H 0.00000000 0.00000000 0.37207500

H 0.00000000 0.00000000 -0.37207500
